# A dTDP-L-rhamnose 4-epimerase required for glycopeptidolipid biosynthesis in *Mycobacterium abscessus*

**DOI:** 10.1016/j.jbc.2024.107852

**Published:** 2024-10-01

**Authors:** John Jairo Aguilera-Correa, Fangyu Wei, Louis-David Leclercq, Yara Tasrini, Edukondalu Mullapudi, Wassim Daher, Kazuki Nakajima, Stéphane Canaan, Jean-Louis Herrmann, Matthias Wilmanns, Yann Guérardel, Liuqing Wen, Laurent Kremer

**Affiliations:** 1 Centre National de la Recherche Scientifique UMR 9004, Institut de Recherche en Infectiologie de Montpellier (IRIM), Université de Montpellier, Montpellier, France; 2 Shanghai Institute of Materia Medica, Chinese Academy of Sciences, Shanghai, China; 3 Université de Lille, CNRS, UMR 8576 - UGSF, Unité de Glycobiologie Structurale et Fonctionnelle, Lille, France; 4 Université Paris-Saclay, UVSQ, Inserm, Infection et Inflammation, Montigny-Le-Bretonneux, France; 5 European Molecular Biology Laboratory, Hamburg Unit, Hamburg, Germany; 6 INSERM, IRIM, Montpellier, France; 7 Institute for Glyco-core Research (iGCORE), Gifu University, Gifu, Japan; 8 Aix-Marseille Université, CNRS, LISM, IMM, Marseille, France; 9 University Medical Center Hamburg-Eppendorf, Hamburg, Germany

**Keywords:** *Mycobacterium abscessus*, cell wall, glycopeptidolipid, rhamnose, 6-deoxy-talose, epimerase, morphotype, virulence, zebrafish

## Abstract

*Mycobacterium abscessus* causes severe lung infections in cystic fibrosis patients and exhibits smooth (S) or rough (R) morphotypes. Disruption of glycopeptidolipid (GPL) production results in the S-to-R transition but the underlying molecular mechanisms of this transition remain incompletely understood. Herein, we characterized MAB_4111c in relation to GPL synthesis and investigated the effects of *MAB_4111c* deletion in *M. abscessus* pathogenicity. An enzymatic assay indicated that MAB_4111c, also designated Tle for Talose epimerase, is converting dTDP-L-Rhamnose into dTDP-6-deoxy-L-Talose. A *tle* deletion mutant was constructed in the S variant of *M. abscessus* and relative areas of Rhamnose and 6-deoxy-Talose and their methylated forms expressed as ratios of total monosaccharides, showed an altered GPL profile lacking 6-deoxy-Talose. Thus, Tle provides dTDP-6-deoxy-L-Talose, subsequently used by the glycosyltransferase Gtf1 to transfer 6-deoxy-Talose to the GPL backbone. Strikingly, the *tle* mutant exhibited an R morphotype, showed impaired sliding motility and biofilm formation, and these phenotypes were rescued upon functional complementation. Moreover, deletion of *tle* in *M. abscessus* results in increased pathogenicity and killing in zebrafish embryos. Together, our results underscore the importance of the dTDP-L-Rhamnose 4-epimerase activity in GPL biosynthesis and in influencing *M*. *abscessus* virulence.

*Mycobacterium abscessus* is an emerging opportunistic pathogen causing a variety of human infections that usually involve the skin and subjacent soft tissues, but also severe lung infections in patients with various chronic lung disorders, such as bronchiectasis and chronic obstructive pulmonary disease or cystic fibrosis (1, 2). This mycobacterium is intrinsically resistant to β-lactams, tetracyclines, aminoglycosides, and macrolides, which are routinely used to treat Gram-negative and Gram-positive bacterial infections (3), rendering treatments extremely challenging (4). Like other nontuberculous mycobacteria (NTM), *M. abscessus* has shown the ability to develop biofilms, structured bacterial aggregates enclosed in a self-produced polymeric matrix where multiple and complex socio-microbiological relationships reign, in the environment, water distribution systems (5, 6), hospital equipment (5, 6), human tissues, for example, lungs (7), and medical devices (8, 9). This rapidly-growing mycobacterium can be found as smooth (S) or rough (R) variants and these morphotypes rely mainly on the production of high (in S) or low (in R) levels of glycopeptidolipids (GPL), respectively (10, 11, 12). Genome sequencing identified multiple indels in the *gpl* locus of the R strain as compared to the S strain (13) mainly in *mps1* and *mps2,* encoding nonribosomal peptide synthases responsible for the biosynthesis of the GPL peptide backbone, and in *mmpL4a*/*mmpL4b,* encoding membrane proteins involved in GPL transport (13, 14, 15). Thus, these genetic changes in the *gpl* locus result in the irreversible loss of the GPL and are directly responsible for the R colony morphology (13).

GPL are found in the outer leaflet of numerous NTM (16). These lipids are pivotal for aggregation, biofilm development, motility, interaction with host cells and pathogenesis (16, 17, 18, 19, 20, 21, 22). Both S and R forms are isolated from sputums of patients with chronic lung infections but while the S form is thought to represent the environmental variant of *M. abscessus* (which infects and colonizes the lungs), the R form emerges from the S form within the infected host (18, 23, 24). Several studies support the enhanced pathogenesis of the R form over the S form, the former being associated with more persisting and aggressive infections in addition to the decline of the lung functions (18, 25, 26, 27, 28). In the context of infection with *M. abscessus*, the zebrafish model has deserved considerable interest as it was used to define the interactions between *M. abscessus* and the innate immune response (27, 29). In particular, zebrafish embryos are killed by the *M. abscessus* R infection, as evidenced by intense bacterial proliferation, production of serpentine cords, abscesses, and inflammation (27, 30). These features are in agreement with epidemiological surveys, which support the prominence of the R variant in patients with severe lung infections and chronic airway colonization in cystic fibrosis patients (18, 24). Overall, this highlights the clinical relevance of morphological distinctions between S and R morphotypes and importance to understand the S-to-R transition at a molecular level (13).

The general GPL structure consists of a mixture of 3-hydroxy and 3-methoxy C28 to C30 fatty acids amidated by a tripeptide-amino-alcohol (D-Phe-D-*allo*-Thr-D-Ala-L-alaninol) (10). This lipopeptide core is glycosylated on the *allo*-Thr linked with a 6-deoxy-α-L-Talose (6-d-Tal) and on the alaninol linked with an α-L-Rhamnose (Rha) (10). Some GPL can be di-glycosylated containing a 3,4-di-*O*-acetylated 6-d-Tal and a 3,4-di-*O*-methylated or 2,3,4-tri-*O*-methylated Rha (31, 32, 33, 34). We previously demonstrated that the glycosyltransferases Gtf1, Gtf2, and Gtf3, encoded within the *gpl* locus, transfer the 6-d-Tal, the first Rha and the second Rha to the lipopeptide core, respectively (35). Importantly, in a *gtf1* deletion mutant, the synthesis of GPL derivatives lacking 6-d-Tal leads to an R morphotype, mycobacterial cording and increased virulence and pathogenicity in zebrafish (35). This illustrates the importance of Gtf1 in modeling the GPL structure and in influencing *M*. *abscessus* virulence, although the functional and structural characterization of this enzyme remains to be established. In addition, the enzyme/pathway that provides dTDP-6-d-Tal, the substrate used by Gtf1 to talosylate GPL, remains unknown.

In mycobacteria, L-Rha is synthesized *via* the essential Rml pathway that generates dTDP-L-Rha from Glc-1-phosphate (36). This pathway is composed of four enzymes—RmlA (MAB_4113), RmlB (MAB_3779), RmlC (MAB_3780), and RmlD (MAB_3613c)—encoded by genes that are not in close vicinity in the *M. abscessus* genome, except for *rmlB* and *rmlC*. Besides being a component of GPL, Rha can be found in other glycolipids, such as phenolic glycolipids (37) and is present in all mycobacteria as being part of the linker disaccharide (Rha-N-acetyl-glucosaminyl-phosphate) unit that attaches the cell wall arabinogalactan to the peptidoglycan layer (36). In contrast to Rha, 6-d-Tal is an infrequent deoxyhexose found only in bacteria, essentially in the cell wall and capsules (38). The dTDP-6-d-Tal is the activated form of 6-d-Tal and it is synthesized from Glc-1-phosphate and dTTP partially or entirely *via* the Rml pathway. In *Haemophilus actinomycetemcomitans* NCTC9710 serotype C (partial synthesis *via* the Rml pathway), the first three enzymes, RmlA-C, are identical to those required to synthesize dTDP-L-Rha. The fourth enzyme, a dTDP-6-deoxy-L-talose 4-dehydrogenase (Tll) is also a dTDP-4-keto-6-deoxy-L-mannose reductase such as RmlD, but the stereoselectivity of Tll regulates dTDP-4-keto-6-deoxy-L-mannose, which is reduced to dTDP-6-deoxy-L-Talose (39). In *Burkholderia thailandensis* (complete synthesis *via* the Rml pathway), a dTDP-L-Rha 4-epimerase, WbiB, converts dTDP-L-Rha into dTDP-6-d-Tal (40). Interestingly, in *M. abscessus*, a paralogous *rmlB* gene (*MAB_4111c*) is present in the *gpl* locus (31) and *in silico* predictions suggest that *MAB_4111c* encodes a putative epimerase/dehydratase (41).

Herein, we focused on MAB_4111c to further advance our understanding of the synthesis of the dTDP-6-d-Tal substrate of Gtf1 and the presence of 6-d-Tal in the GPL of *M. abscessus.* We also aimed to study the consequences of *MAB_4111c* deletion on bacteriological features and disease pathology associated with this emerging pathogen in zebrafish. Bioinformatic and genetic studies were also performed to determine the distribution and prevalence of *MAB_4111c* orthologous genes in other NTMs.

## Results

### *MAB_4111c* encodes a preditive dTDP-L-rhamnose 4-epimerase

**Figure 1:**
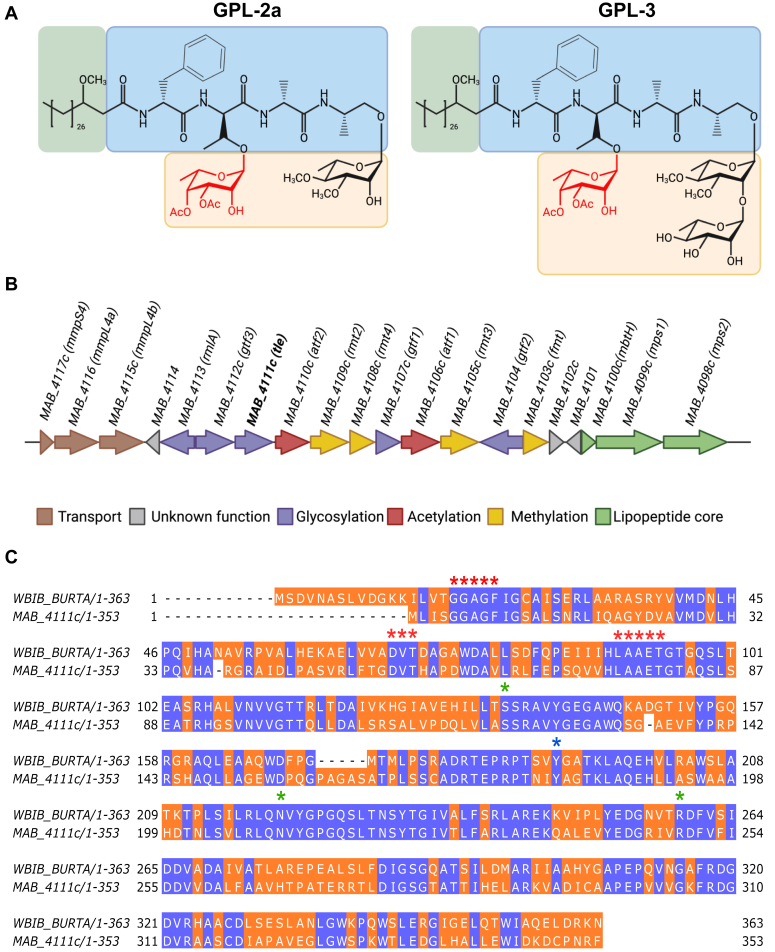
**MAB_4111c belongs to the GPL biosynthetic cluster and is a predicted epimerase.***A*, structure of diglycosylated (GPL-2a) and triglycosylated (GPL-3) glycopeptidolipids. The *green*, *blue*, and *yellow* shadings represent the lipid, peptide, and glycosydic moieties of the GPL. Di-*O*-acetyl 6-d-Tal is drawn in *red*. *B*, *Mycobacterium abscessus gpl* locus encoding enzymes involved in the synthesis, modification, and transport of GPL. *MAB_4111c* is indicated in *bold*. *C*, alignment of the protein sequence of dTDP-L-Rha 4-epimerase from the *Burkholderia thailandensis* (ATCC 700388) (wbiB_BURTA) with that of MAB_4111c from *M. abscessus*. The *orange* and *blue* shadings represent the nonconserved and conserved and amino acids, respectively. The *red*, *green*, and *blue asterisks* indicate residues involved in the putative NADH-binding site, the substrate-binding site, and the catalytic residue reported in *B. thailandensis*, respectively.

The S variant of *M. abscessus* synthesizes both diglycosylated GPL (GPL-2a) and triglycosylated GPL (GPL-3), containing one and two partially *O*-methylated Rha residues, respectively, as well as a di-*O*-acetyl 6-d-Tal residue (Fig. 1*A*). While most proteins encoded by the *gpl* cluster and involved in the biosynthesis and transport of GPL have been characterized to date, the enzyme catalyzing the interconversion between dTDP-Rha and dTDP-6-d-Tal in mycobacteria remains enigmatic. *MAB_4111c* is located between *gtf3*, encoding the rhamnosyltransferase which adds the second Rha to generate GPL-3 (35), and *atf2*, encoding an acetyltransferase which acetylates the 6-d-Tal (34) (Fig. 1*B*). Bioinformatics analysis suggests that *MAB_4111c* may code for an enzyme with epimerase activity, in agreement with a BLAST analysis which shows that MAB_4111c shares 54% identity with WbiB, a dTDP-Rha 4-epimerase in *Burkholderia thailandensis*, the first enzyme reported to catalyze the interconversion between dTDP-Rha and dTDP-6-d-Tal (40). This analysis also shows that the putative catalytic Tyr residue (Y181) as well as other residues participating in the binding of the NADH cofactor and dTDP-Rha substrate are fully conserved in MAB_4111c (Fig. 1*C*).

### MAB_4111c converts dTDP-Rhamnose into dTDP-6-deoxy-talose

**Figure 2:**
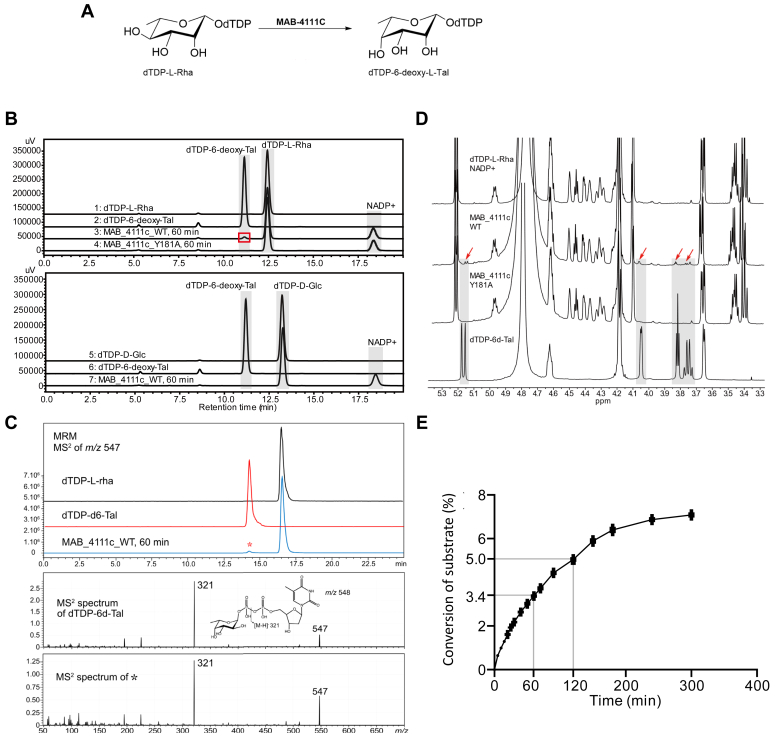
**Enzymatic activity of MAB_4111c.***A*, reaction catalyzed by MAB_4111c. *B*, *upper panel*: HPLC profile of the standards and products formed during the enzymatic reaction in the presence of MAB_4111c or MAB_4111c_Y181A with dTDP-Rha and NADP^+^. The incubation was performed for 60 min. The product of the reaction is labeled with a *red box*. *Lower panel*: HPLC profile of standard dTDP-Glc, standard dTDP-6-d-Tal, and dTDP-Glc, and NADP^+^ incubated with MAB_4111c for 60 min. *C*, *upper panel*: LC/MS profile of the enzymatic reaction mixture in MRM mode using the specific precursor ion [M-H]^−^ at *m/z* 547.1. *Lower panel*: MS^2^ fragmentation spectra of standard dTDP-6-d-Tal and the enzymatic reaction product (∗). *D*, ^1^H NMR spectra of dTDP-L-Rha and NADP^+^; MAB_4111c with dTDP-L-Rha and NADP^+^ after 120 min; MAB_4111c_Y181A with dTDP-L-Rha and NADP^+^ after 120 min; dTDP-6-d-Tal. The characteristic peaks of dTDP-6-d-Tal are indicated with *red arrows*. *E*, kinetic analysis of the reaction catalyzed by MAB_4111c in the presence of dTDP-L-Rha and NADP^+^.

To experimentally validate the function of MAB_4111c as a dTDP-Rha 4-epimerase, a His-tagged version of the protein was produced in *Escherichia coli.* Initial attempts to express and purify soluble MAB_4111c for biochemical and structural analyses yielded insufficient protein quantities. Thus, we generated a MAB_4111c-enriched soluble fraction obtained after partial purification on Ni^2+^-coated beads, which was assayed by incubation with NADP^+^ and dTDP-Rha and monitored for the production of dTDP-6-d-Tal (Fig. 2*A*). The HPLC profile shows a weak signal with a retention time similar to the one of the dTDP-6-d-Tal standard (Fig. 2*B*, *upper panel*). The nature of the observed weak signal was assessed by LC/MS analysis in multiple reaction monitoring (MRM) mode (Fig. 2*C*). As shown in the upper panel, the specific precursor ion [M-H]^−^ at *m/z* 547.1, characteristic of dTDP-dHex, showed two signals with retention times similar to dTDP-Rha and dTDP-6-d-Tal. Fragmentation of the minor signal produced a MS/MS spectrum identical to standard dTDP-6-d-Tal, demonstrating that the minor signal corresponds to dTDP-6-d-Tal (Fig. 2*C*, *lower panel*). Finally, NMR analysis of the total reaction mixture showed signals characteristic of dTDP-6-d-Tal, along with signals attributed to the substrate dTDP-Rha, further demonstrating that MAB_4111c transforms dTDP-Rha into dTDP-6-d-Tal (Fig. 2*D*). Specifically, weak proton signals at 5.17, 4.05, 3.82, and 3.75 ppm are characteristic signals of dTDP-6-d-Tal. According to the NMR spectrum of the dTDP-6-d-Tal standard, proton signals were elucidated as H1 (δ5.17 ppm), H2 (δ4.05 ppm), H3 (δ3.82 ppm), and H5 (δ3.75 ppm) of 6-d-Tal. The signal of H-4 proton (δ3.65 ppm) in 6-d-Tal is overlapped with H-4 proton signal of Rha. Kinetic analysis indicated that MAB_4111c could convert dTDP-Rha into dTDP-6-d-Tal with a 3 to 4% conversion rate within an hour, reaching a 7% conversion rate after 300 min of incubation (Fig. 2*E*). To address the specificity of the reaction, a similar assay was set up by replacing dTDP-Rha with dTDP-Glc in the reaction mixture, where no dTDP-6-d-Tal was detected (Fig. 2*B*, *lower panel*). In addition, we also assayed the reverse reaction using dTDP-6-d-Tal as a substrate and observed the formation of dTDP-Rha after 60 min of reaction (data not shown), thus confirming that MAB_4111c catalyzes both reactions.

Overall, these results suggest that MAB_4111c is an epimerase that specifically converts dTDP-Rha into dTDP-6-d-Tal *in vitro*. Given its activity, we propose to rename the protein Tle for Talose epimerase, based on a previous nomenclature used to designate a similar enzyme in *Acinetobacter baumannii* (42).

### Structural prediction of Tle

**Figure 3:**
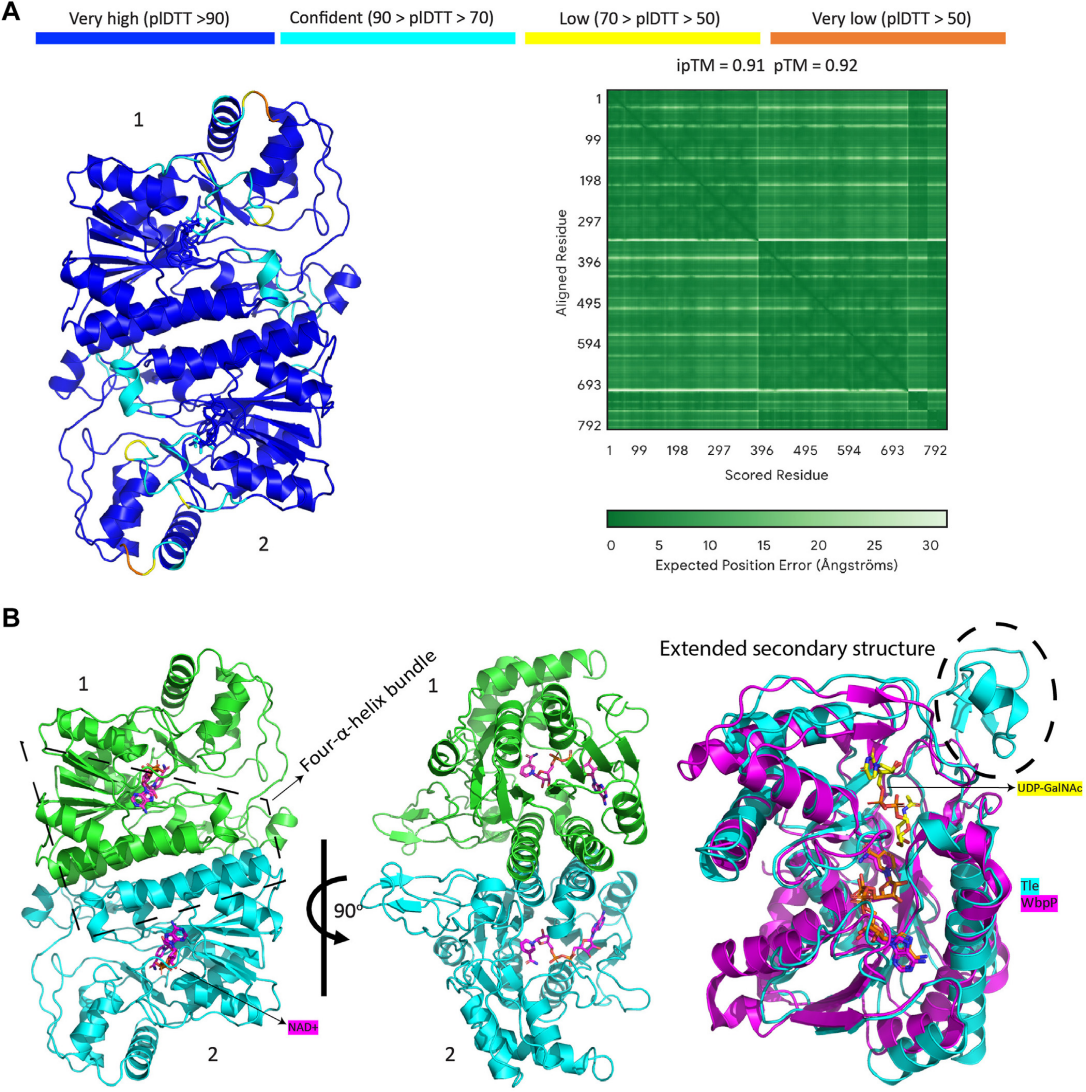
**Structural prediction of Tle.***A*, cartoon representation of the Alphafold 3 predicted dimeric structure of Tle. The structure is colored according to the plDTT score range (*blue* = high confidence, *cyan* = confidence, *yellow* = low confidence, *orange* = very low confidence). *B*, *left panel*: cartoon representation of the predicted Tle homodimer, with each chain colored in *green* and *cyan*. NAD is depicted as a stick model. *Right panel*: alignment of the monomeric structure of Tle with the WbpP crystal structure (Tle in *cyan* and WbpP in *magenta*).

Since we could not produce high yields of Tle for structural analysis, we employed AlphaFold 3 for structural predictions (43). We first screened the Protein Data Bank for experimentally determined epimerase structures, most of which are dimeric. Sequence identity between Tle and available epimerase structures ranged from approximately 23 to 32%. Despite low sequence identity, epimerases typically exhibit high structural homology. Using AlphaFold 3, we generated a dimeric structure of Tle in the presence of NAD^+^. The prediction covered more than 90% of the structure with a very high predicted local distance difference test (pLDDT) score (Fig. 3*A*). The oligomeric state prediction yielded predicted template modeling and interface predicted template modeling values of 0.91 and 0.92, respectively, indicating that the predicted structure can be used with confidence for further analysis. The structure reveals a classic short-chain dehydrogenase/reductase (SDR) fold (44) (Fig. 3*B*). The dimeric interface is formed by two α-helices from each protomer (residues 88–110 and 180–199), creating a four-α-helix bundle (Fig. 3*B*). The overall architecture includes an N-terminal NAD^+^ binding domain, a typical Rossmann fold and a C-terminal substrate-binding domain (Fig. 3*B*). Comparison of the AlphaFold-generated Tle structure with the known *Pseudomonas aeruginosa* UDP-N-acetylglucosamine 4-epimerase WbpP (PDB code: 1SB8) epimerase structure yielded a root mean square deviation of approximately 1.37 Å (Fig. 3*B*), suggesting a high degree of structural similarity. WbpP was used because it had one of the best root mean square deviation scores in our analysis. Notably, Tle includes an extended secondary structure element from residues 128 to 165, characterized by an additional α-helix, long loop, and a pair of antiparallel β-strands. This feature contrasts with WbpP, which has a shorter loop in the corresponding region, highlighting a distinctive structural variation in Tle (Fig. 3*B*).

We next compared the AlphaFold-predicted structure of Tle with the known WbpP structure to identify the key residues involved in catalysis. The binding site for NAD^+^ is highly conserved among SDR family enzymes (44). Structural alignment with WbpP confirmed that majority of the NAD^+^-binding residues are conserved in Tle (Fig. S1*A*). However, some differences were noted: V30, H32, and H36 in Tle correspond to N48, A50, and G52 in WbpP (Fig. S1*B*). The catalytic triad, which is typically represented as SYK in the SDR family (44, 45, 46), is conserved in Tle as S121, Y181, and K185 (Fig. S1*B*). No activity was observed when replacing the WT enzyme by the Tle-Y181A mutant in the assay (Fig. 2, *B* and *D*), thus indicating that introducing a Y181A change leads to a catalytically inactive enzyme. We also assessed the activity of Tle-mutated proteins in which the conserved residues S121, N210, R249 (Figs. 1*C* and S1*B*) were substitued by alanines. As for the His-tagged WT protein, the different mutated variants were enriched by affinity chromatography on Ni^2+^ beads and the amount of each protein estimated by Western blotting using anti-His antibodies (Fig. S2*A*). The relative concentration of each protein was determined by comparing the grayscale of the bands using ImageJ and adjusted so that the same quantity of each protein was added to the assay in the presence of NADP^+^ and dTDP-Rha. Figure S2*B* shows that none of these mutated proteins catalyzed the formation of dTDP-6-d-Tal, confirming the importance of S121, N210, and R249 in the activity of Tle.

Overall, these data validate the AlphaFold model and supports the structural similarity between WbpP and Tle.

### Bacteriological characterization of a *tle* deletion mutant in *M. abscessus*

To investigate the contribution and biological functions of the dTDP-L-Rha 4-epimerase in *M. abscessus*, *tle* was deleted in the GPL-producing S variant of *M. abscessus,* using an unmarked deletion method (47) (Fig. S3*A*). PCR/sequencing performed on the parental and mutant strain (Δ*tle*) using the primers listed in Table S1 confirmed the proper genotype of the mutant (Fig. S3*B*). Genetic complementation of the mutant was done through specific integration at the *attB* chromosomal site (48) of a copy of *tle* fused to a GFP-tag at the 3′-end and placed under the control of the endogenous promoter. Probing the crude lysates from the various strains using anti-GFP antibodies revealed a single band of the expected size, corresponding to the Tle-GFP fusion protein, validating complementation of the mutant (Fig. S3*C*).

**Figure 4:**
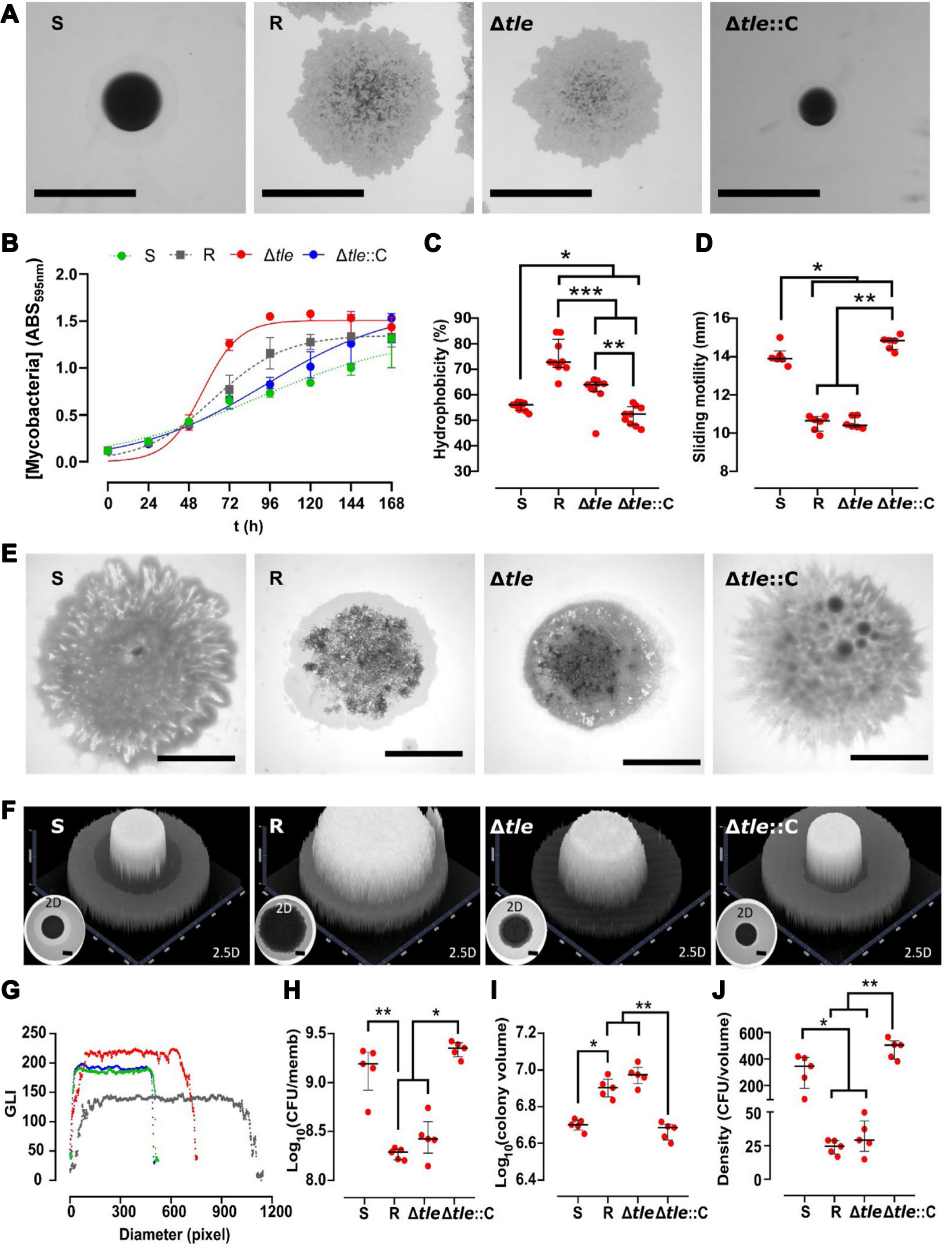
**Deletion of *tle* in *M. abscessus* alters colony morphology, hydrophobicity, sliding motility, and biofilm-colony development.***A*, knock-out of *tle* is associated with a switch from an S to an R-like morphotype. The scale bar represents 1 mm. *B*, the deletion of *tle* is associated with a higher growth rate. The experiment was performed using two biological replicates with six technical replicates per biological replicate. Deletion of *tle* modifies hydrophobicity (*C*) and the sliding motility (*D* and *E*). The hydrophobicity experiment was performed using three biological replicates with three technical replicates per biological replicate. The sliding motility experiment was performed using three biological replicates with two technical replicates per biological replicate. The *black scale bar* indicates 5 mm. *F*, representative 2- and 2.5-dimensional pictures of colony-biofilms on biofilm-supporting membranes after 5 days of incubation. The absence of *tle* alters the biofilm-colony growth (*F*) (the scale bar represents 2 mm) and the biofilm-colony profile (*G*) (*green*, *dark grey*, *red*, and *blue dots* represent the profiles of S, R, Δ*tle* and Δ*tle*::C, respectively). The CFU per membrane are shown in (*H*), the colony volume in (*I*), and the colony density in (*J*). This experiment was performed using five biological replicates. The error bar denotes the interquartile range. ∗: *p* < 0.05, ∗∗: *p* < 0.01, and ∗∗∗: *p* < 0.001. CFU, colony-forming units.

A previous work had shown that deletion of *gtf1* in *M. abscessus* S was associated with R morphotype on agar plates (35). Observation of individual colonies showed that Δ*tle* formed R, corded colonies (Fig. 4*A*). Complementation of Δ*tle* (Δ*tle::*C) rescued the S morphotype (Fig. 4*A*). The growth of all strains in liquid culture describes a logistic growth (R^2^ > 0.9 for all of them) (Fig. 4*B* and Table S4). The growth rate of Δ*tle* was higher than that of the R strain (*p* < 0.0001), while the one of Δ*tle::*C was similar to the growth rate of the parental S strain. This indicates that deletion of *tle* slightly impacts on the replication rate of *M. abscessus* in planktonic culture. However, additional transmission electron microscopy observations failed to show significant changes regarding the size and ultrastructural organization of the cell wall in Δ*tle* (Fig. S4). Partitioning of mycobacterial pellets between hexadecane and aqueous buffer is used as a quantitative marker of cell surface hydrophobicity in mycobacteria to associate low pathogenicity with reduced hydrophobicity (49). To address whether deletion of *tle* affects surface hydrophobicity, aqueous hexadecane-buffer partitioning was applied to the S, R, Δ*tle*, and Δ*tle::*C strains. Δ*tle* appeared more hydrophobic than the parental S strain, in agreement with its R morphotype while the complemented strain displayed a hydrophobicity level similar to the parental S progenitor, correlating with its smoother morphological aspect (Fig. 4*C*). Consistent with the ability of the *M. abscessus* S variants to slide from the center towards the periphery of the plates (11, 28), Δ*tle::*C, but neither R nor Δ*tle,* showed a higher sliding motility (Fig. 4, *D* and *E*). Since sliding is dependent on the bacterial surface properties and interactions with the substrate, this suggests that Δ*tle* is likely defective in surface-associated cell wall components. The Δ*tle* colony biofilm was intermediate between the S and R biofilms (Fig. 4*F*), being broader and higher than the S biofilm (Figs. 4, *F* and *G* and S5). In addition, the number of colony-forming units (CFU) per membrane (Fig. 4*H*), the colony volume (Fig. 4*I*), and density of the biofilm (Fig. 4*J*) were very similar between the R and Δ*tle* strains. Δ*tle::C* showed no difference with the S progenitor.

We next investigated whether *tle* deletion affects drug susceptibility of *M. abscessus* towards a large panel of clinically used antibiotics. Δ*tle* showed only very slight changes in its antibiotype (Table S5), for instance, a slight increase against amikacin (MIC of 16 μg/ml for the WT strain and 32 μg/ml for Δ*tle*), tigecycline (MIC of 0.5 μg/ml for the WT strain to 1 μg/ml for Δ*tle*), and a slight reduction against imipenem (MIC of 64 μg/ml for the WT to 32 μg/ml for Δ*tle*), clarithromycin (MIC of 8 μg/ml for the WT strain and 4 μg/ml for Δ*tle*), or rifabutin (MIC of 64 μg/ml for the WT strain and 32 μg/ml for Δ*tle*).

### Deletion of *tle* leads to the production of GPL lacking 6-deoxy-talose

**Figure 5:**
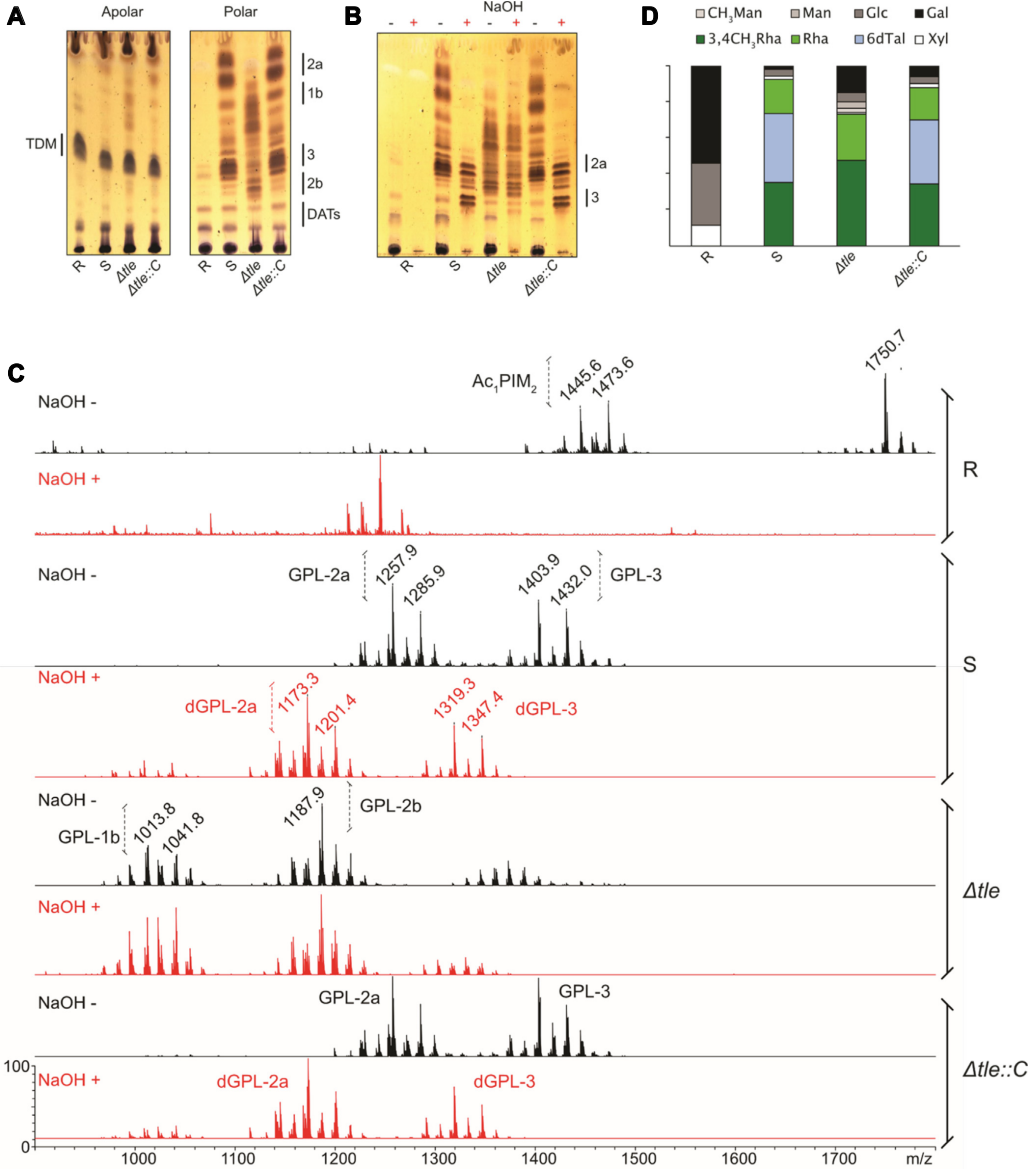
**Structural analysis of the GPL content in Δ*tle.****A*, TLC analysis representative from three independent experiments of native apolar (*left*) and polar (*right*) lipid extracts. Lipids were separated using chloroform/methanol/water (90:10:1, v/v/v) followed by spraying with orcinol and charring. GPL-1b and GPL-2b previously described (35) from Δ*tle* display a lower R_f_ than WT GPL-2a and GPL-3, which are restored in Δ*tle::*C. TDM: trehalose dimycolate; DAT: Di-*O*-acyl trehalose. *B*, TLC analysis of native (NaOH−) and saponified (NaOH+) polar fractions. Lipids were separated using chloroform/methanol/water (90:10:1, v/v/v) followed by spraying with orcinol and charring. The R_f_ of GPL-2a and GPL-3 are identical in S and Δ*tle::*C but not in Δ*tle*. *C*, MALDI-TOF positive MS spectra of native polar lipid fraction (*black*, NaOH−) from the S and Δ*tle::*C strains show ions for GPL-2a at *m/z* 1257.9/1285.9 and for GPL-3 at *m/z* 1403.9/1432.0. MS spectra from saponified polar lipid fraction (*red*, NaOH+) show a 84 u.m.a decrease, to 1173.4/1201.4 and 1319.3/1347.4, corresponding to deacetylated dGPL-2a and dGPL-3, as previously described (34). Conversely, ions for GPL-1b at *m/z* 1013.8/1041.8 and from GPL-2b at 1187.9 were intense in native and saponified polar extracts from Δ*tle. D*, relative proportion of each monosaccharides was determined in the GPL-enriched saponified polar lipid fraction. 6-d-Tal could only be detected in S and Δ*tle::*C.

We next evaluated the impact of *tle* deletion on the glycolipid content extracted from lyophilized mycobacteria. Analysis of the apolar lipid fraction by TLC shows that trehalose 6,6′-dimycolate remains equally produced in all tested strains (Fig. 5*A*, *left panel*). Analysis of the polar lipid fractions indicates that, except in the R strain lacking GPL (33, 35), GPL are produced at high levels in all strains (Fig. 5*A*, *right panel*). However, while the parental S strain and Δ*tle::*C displayed an identical GPL profile, Δ*tle* exhibited lower migrating GPLs, resembling to those produced by Δ*gtf1* (Figs. 5*A right panel* and S6*A*), a previously characterized mutant lacking the Gtf1 glycosyltransferase, which adds 6-d-Tal to the peptide backbone (35). Mild alkaline hydrolysis deacetylated polar lipids from S and Δ*tle::*C, as shown by the lower R_f_ of NaOH-treated compared to untreated ones GPL (Fig. 5*B*). In contrast, saponification did not influence the GPL pattern of Δ*tle* and Δ*gtf1* (Figs. 5*B* and S6*C*), suggesting that they are not acetylated, as previously demonstrated for Δ*gtf1* (35). Mass spectrometry analysis of native polar lipids from S and Δ*tle::*C showed ions at *m/z* 1258/1286 and 1404/1432 (Fig. 5*C*), previously identified as GPL-2a and GPL-3 (Fig. 1*A*) (33, 34). After saponification, M-84 u.m.a ions at *m/z* 1173/1201 and 1319/1347 were detected, corresponding to dGPL-2a and dGPL-3, respectively, in agreement with the loss of the two acetyl groups substituting 6-d-Tal (Figs. 5*C* and S6, *B* and *D*). MS spectra of native and saponified polar lipids isolated from Δ*tle* and Δ*gtf1,* all showed an intense ion at *m/z* 1187 accompanied with 1013/1041 pattern (Figs. 5*C* and S6, *B* and *D*), previously attributed to the unnatural isomers GPL-2b and GPL-1b, which are devoid of 6-d-Tal (35). The absence of the 6-d-Tal was further confirmed by the monosaccharide composition analysis of the polar lipids, which demonstrates the lack of 6-d-Tal in Δ*tle* and Δ*gtf1* and its restoration in Δ*tle::*C (Figs. 5*D* and S6*E*).

### Distribution of *tle* orthologs in NTM and other bacteria

**Figure 6:**
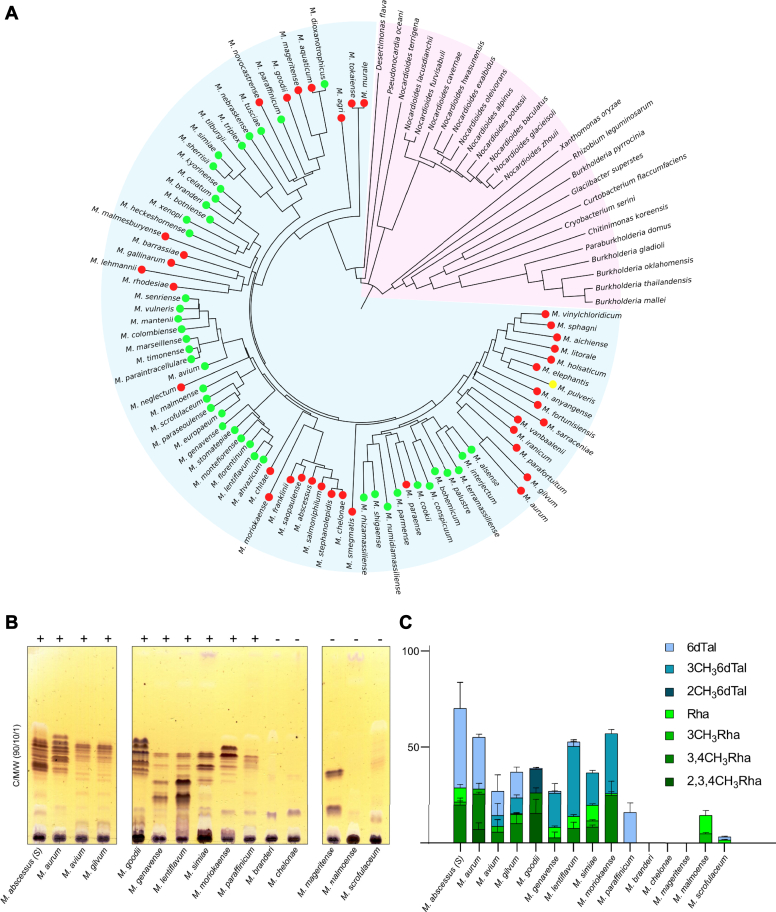
**Distribution of *tle* orthologs in nontuberculous mycobacteria and correlation with the presence of 6-d-Tal.***A*, phylogenetic tree reconstructed using already available *tle* sequences. Nonmycobacterial species and mycobacterial species are represented in *pink* and *blue areas*, respectively. *Green*, *yellow*, and *red dots* label slowly-growing, intermediately-growing, and rapid-growing mycobacteria, respectively. *B*, TLC analysis of the polar lipid fraction of a selected panel of NTM clinical strains, representative from two independent experiments. Lipids were separated using chloroform/methanol/water (90:10:1, v/v/v) followed by spraying with orcinol and charring. +: presence of GPL-like components, confirmed by their detection after saponification (Fig. S8). −: absence of GPL-like components. *C*, Deoxyhexose composition analysis of polar lipid fractions by GC/FID and GC/MS. Relative area of identified monosaccharides (n = 4) show Rha residues in *green* and 6-d-Tal in *blue*. Identification of 6-d-Tal and Rha was confirmed by the retention time and fragmentation pattern of standards while *O-*methylated Rha was determined previously (33, 35). Identification of 2- and 3*-O-*methyl 6-d-Tal was deduced from the retention time and fragmentation pattern. Relative areas of Rha and 6-d-Tal and their methylated forms are expressed as ratios of total monosaccharides.

We identified and analyzed 107 proteins orthologous to Tle considering the inclusion and exclusion criteria described in the Experimental procedures. The neighbour-joining phylogenetic tree showed two clear clades related to mycobacterial genes and other bacterial species. Orthologous were found in at least 26 bacterial species belonging to the phyla Actinomycetota (genera *Nocardioides*, *Pseudonocardia*, *Desertimonas*, *Glaciibater*, *Curtobacterium*, and *Cryobacterium*) and Pseudomonadota (genera *Burkholderia*, *Paraburkholderia*, *Xanthomonas*, *Rhizobium*, and *Chitinimonas*). Seventy-nine mycobacterial species, excluding *M. abscessus*, possess an orthologous gene to *tle* based on amino acid sequence analysis (Fig. 6*A*). These included 42 slowly-growing mycobacteria, one intermediately-growing *mycobacterium*, and 36 rapidly-growing mycobacteria. This phylogenetic analysis indicates that Tle-related proteins can be found in bacteria but are over-represented in mycobacteria. It also suggests that these orthologs may have descended from a common ancestor and, therefore, that they can be viewed as orthologous members of the same protein family.

In mycobacteria, 6-d-Tal has been only described in GPL so far. We next investigated the glycolipid profile, focusing on GPL from a set of slow-growing and rapidly-growing clinical nontuberculous mycobacterial isolates, which are representative strains present in the phylogenetic tree (Fig. 6*A*). TLC analysis of the apolar lipid fraction of all species from a broad panel showed an intense band around R_f_ 0.43 that was attributed to trehalose 6,6′-dimycolate (Fig. S7*A*). TLC analysis of polar lipids in chloroform/methanol/water (65:25:4, v/v/v) also showed phospho-*myo*-inositol mannosides in all species (Fig. S7*B*). Subsequent TLC analysis using chloroform/methanol/water (90:10:1, v/v/v) revealed a wider lipid diversity, including GPL-like compounds that were differentially expressed (Fig. 6*B*). Overall, of the 15 strains tested, ten strains (marked +) showed bands corresponding to GPL, whereas five (marked −) did not. Some strains showed similar TLC patterns such as *Mycobacterium avium* and *M. gilvum* or *M. genavense* and *M. lentiflavum*, suggesting that these pairs of strains have close GPL profiles. The other GPL-positive strains (*M. abscessus*, *M. aurum*, *M. goodii*, *Mycobacterium simiae*, *M. moriokaense*, and *M. paraffinicum*) showed distinct TLC patterns, indicating a large structural variability in their GPL content. As a control, we checked that the identified GPL-related compounds were still detected as lower R_f_ bands following saponification. In contrast, all bands disappeared in *M. branderi*, *M. chelonae*, *M. mageritense*, *M. malmoense*, and *M. scrofulaceum*, further confirming the absence of GPLs in these species (Fig. S8). We further established by GC/FID and GC/MS the monosaccharide composition of the native polar lipid fractions from all the species in order to confirm the species-specific expression of GPLs (Fig. 6*C*). All ten identified GPL-positive species expressed a wide range of deoxyhexose residues, including 6-d-Tal and its methylated-analogs (2*-O-*methyl 6-d-Tal, 3*-O-*methyl 6-d-Tal) and Rha with methylated analogs (3*-O-*methyl Rha, 3,4 di*-O-*methyl Rha, 2,3,4 tri*-O-*methyl Rha) in different ratios. The 6-d-Tal or its methylated forms were detected in all strains, whereas Rha or its methylated forms were detected in all strains except *M. paraffinicum* that exclusively expressed 6-d-Tal. The presence of 3-*O*-methyl 6-d-Tal in *M. genavense*, *M. lentiflavum*, *M. simiae*, and *M. moriokaense* has already been observed in previous studies, in agreement with the present work. In contrast, the polar lipids of three of the five GPL-negative strains (*M. branderi*, *M. chelonae*, *M. mageritense*) did not show any traces of deoxyhexoses. Finally, *M. malmoense* and *M. scrofulaceum* showed low quantities of 6-d-Tal, Rha, and 3*-O-*methyl Rha, suggesting that these two strains may synthetize deoxyhexose independently of GPL. Altogether, these results indicate the exclusive presence of 6-dTal in all GPL-like molecules in mycobacteria, albeit with traces found in *M. scrofulaceum*.

### Deletion of *tle* in *M. abscessus* results in increased pathogenicity in zebrafish

**Figure 7:**
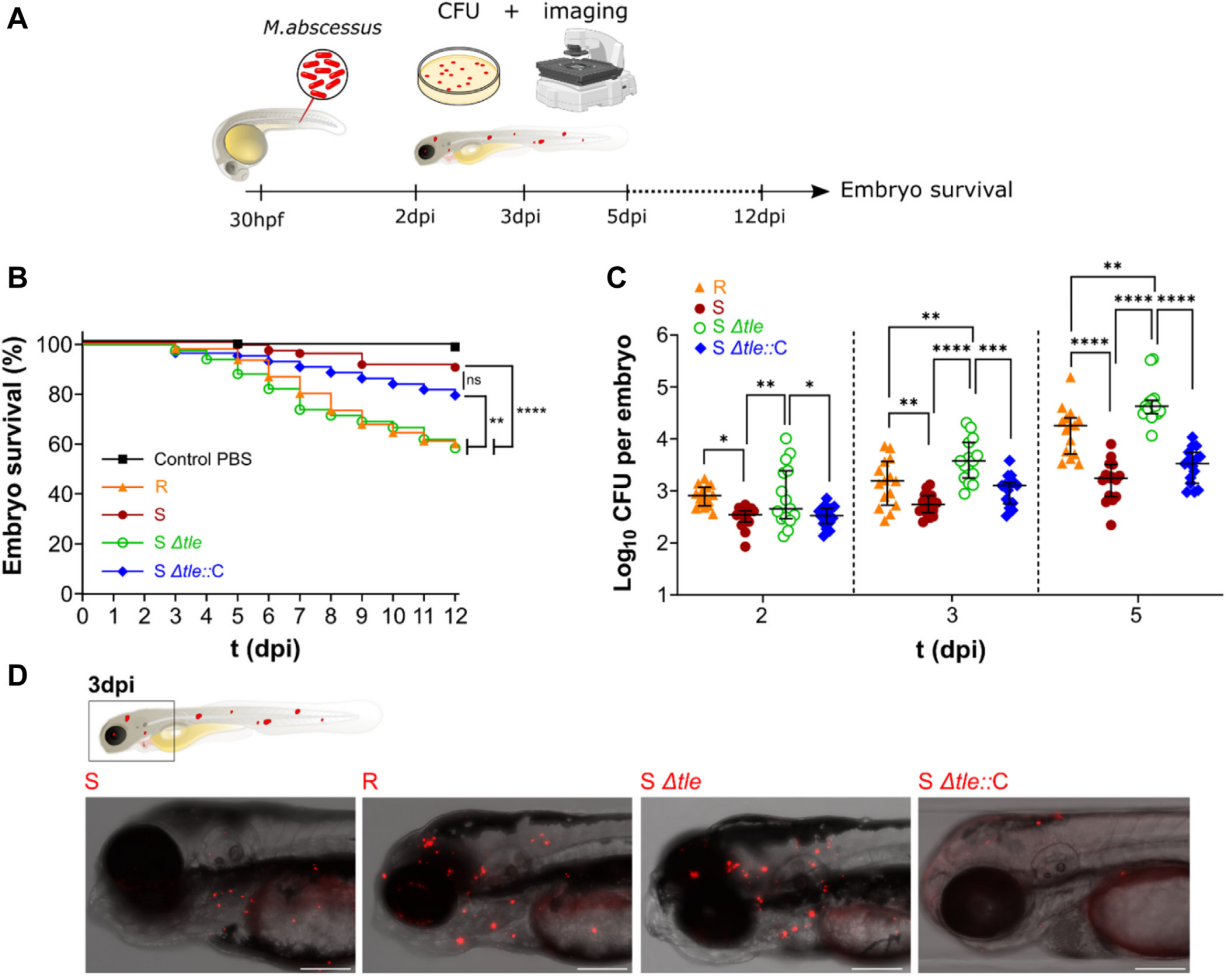
**Increased virulence and pathogenesis of Δ*tle* in a zebrafish embryo model of infection.***A*, schematic overview of *in vivo* experiments. *B*, survival of infected embryos with the R strain (275 ± 27 CFU), the S strain (280 ± 36 CFU), Δ*tle* (264 ± 33 CFU), and Δ*tle::*C (281 ± 47 CFU). The graph shows the result of four pooled independent experiments. Embryos were marked dead in the absence of a heartbeat. Statistical analysis was performed using Log-rank (Mantel-Cox) test. ∗∗∗∗: *p* < 0.0001; ∗∗: *p* < 0.01, ns: non-significant. *C*, bacterial burden from infected embryos at 2, 3, and 5 dpi. The graph shows the result of three pooled independent experiments. n = 5 embryos per time-point per condition. CFU counts were Log_10_ transformed. ∗: *p* < 0.05, ∗∗: *p* < 0.01, ∗∗∗: *p* < 0.001, and ∗∗∗∗: *p* < 0.0001. *D*, imaging of infected embryos at 3 dpi. One zoomed representative image of the head is shown per condition. Scale bars represent 0.2 mm. CFU, colony-forming units.

*M. abscessus* S and R variants have very different infection outcomes when injected in zebrafish embryos (27, 30). While R leads to acute infection and high embryo mortality, S is less virulent and more associated with chronic persistent infection. Therefore, because we observed an R morphoptype upon knocking-out *tle*, we wanted to use the zebrafish model to evaluate the effect of this mutation on the infection outcome. To do so, 30 hpf embryos were infected with approximately 250 CFU of either R, S, Δ*tle*, Δ*tle::*C (all strains expressing a red fluorescent marker), or the PBS control and monitored for embryo survival, bacterial burden, and whole embryo imaging as outlined in Figure 7*A*. As expected, mortality of embryos injected with Δ*tle* was much higher than the S wild-type progenitor (10% and 42%, respectively) and more like the R morphotype (40%) at 12 dpi (Fig. 7*B*). Complementation was only partial, similar to what was observed *in vitro*, with 21% of embryo mortality. In agreement with the enhanced embryo mortality, the bacterial burden was significantly higher in embryos infected with Δ*tle,* to levels close to the R strain, compared to the S progenitor while infection with Δ*tle::*C led to bacterial loads comparable to those found with the S strain (Fig. 7*C*). These results were confirmed by embryo imaging, where we can clearly see very small infection foci in embryos injected with S and the complemented strain at 3 dpi (Fig. 7*D*), while embryos injected with the mutant were similar to the R, with a higher number of infected foci as well as a bigger size.

## Discussion

6-deoxy-Talose (6-d-Tal) is a rare deoxyhexose found in Gram-negative, Gram-positive bacteria, and mycobacteria as a component of the cell wall and capsule structures (10, 35, 38, 42). The activated nucleotide sugar form of 6-d-Tal is dTDP-6-d-Tal, which is synthesized from Glc-1-phosphate and dTTP *via* the Rml pathway. Until now, the origin of the dTDP-6-d-Tal used by Gtf1 for talosylation of GPL remained unknown. Based on the structural predictions with validated epimerases and the development of an enzymatic assay, we demonstrate here that MAB_4111c/Tle catalyzes the epimerization of dTDP-Rha to dTDP-6-d-Tal, an activity lost when the catalytic Y181 or substrate-binding residues are replaced with alanine. Since the epimerization to TDP-6-d-Tal appears only partial *in vitro*, it is possible that *in vivo*, the activity of Tle is coupled with and/or regulated by the cognate glycosyltransferase Gtf1, previously shown to attach the talose residue to the GPL peptidic core (35).

Genomic analyses further showed that *tle* orthologs are widely distributed in actinobacteria and was found in 42% of all mycobacterial species for which a genome sequence is available (50). Even though GPL from various species appear structurally different, our compositional analysis indicates that all contain 6-d-Tal (or 2-*O*-methyl-6-d-Tal or 3-*O*-methyl-6-d-Tal) and that these species all possess a *tle* gene. Conversely, a few species failed to produce GPL despite the presence of *tle*, although it is very likely that these strains (at least some of them) do not produce GPL due to the presence of mutations in the *gpl* biosynthetic and transport gene cluster, as demonstrated previously in *M. abscessus* (13). *M. avium* represents another typical example of an NTM producing GPL which encodes a *tle* gene. Interestingly, the lack of 6-d-Tal attached to the *allo*-threonine in an R variant of *M. avium* MAC 104 has been previously reported (51), and the genetic lesion responsible for the loss of 6-d-Tal in this strain corresponded with the deletion of a genomic region containing *gtfA*, an ortholog of *gtf1* (52). This further supports the hypothesis that the loss of 6-d-Tal conditioning an R morphotype is not specific to *M. abscessus* but can be extrapolated to other NTM.

To validate the structure-function relationship between Tle and 6-d-Tal-containing GPL, we generated a *tle* unmarked deletion in the *M. abscessus* S variant. A thorough lipid analysis clearly emphasized the absence of 6-d-Tal in the GPL of the mutant strain, while the presence of Rha residues remained intact. Previously, we characterized a *gft1* mutant, which, similarly to the *tle* mutant, produced GPL lacking 6-d-Tal. Gtf1 uses dTDP-6-d-Tal produced by Tle, as a substrate to glycosylate the D-*allo*-Thr residue. In addition to the loss of 6-d-Tal, both Δ*gtf1* and Δ*tle* share in common other phenotypes. First, deletion of *tle* produces a virtually R morphotype, although it appeared creamy on plates, while deletion of *gtf1* generated colonies with an R and dry texture. Whereas the vast majority of clinical isolates of the R morphotype have mutations in the biosynthetic genes *mps1*, *mps2*, or transporter genes *mmpL4a/mmpL4b/mmpS4* (15), GPL glycosylation can also lead to colony morphological changes (10). This study shows that enzymes involved in the production of nucleotide-activated sugars can also lead to these morphological changes. However, it remains to be established whether mutations in *tle* are responsible for the acquisition of an R morphotype occuring during infection in the host. Second, while GPL are present in the outermost mycobacterial layer (10), their presence/absence or modifications in their structures can profoundly alter the surface properties of *M. abscessus*. Sliding motility may play an important role in surface colonization by mycobacteria in the environment and in the host (35). The *tle* mutant showed considerably reduced sliding motility. Previous studies between S and R strains underscored the correlation between low GPL production and enhanced hydrophobicity with virulence (2, 10). As expected for an R variant, we found that Δ*tle* was more hydrophobic than the parental S variant, as reported previously for the *gf1* mutant (35). Third, the development of biofilms appears crucial for *M. abscessus* infections (53, 54). The deletion of *tle* altered the biofilm development of *M. abscessus*. Although most of the measured parameters (number of bacteria, colony volume, and density) indicated that the *tle* mutant behaved similarly to the R morphotype, its colony shape was significantly different from that of the S and R morphotypes, as the colony height of the *tle* mutant was greater and colony diameter was less wide than that of the reference R strain. This emphasizes the importance of 6-d-Tal for shaping *M. abscessus* colony-biofilms. Herein, we also took advantage of using the zebrafish model of infection as a reliable and manageable model for revealing and comparing the difference in virulence between the parental S and the Δ*tle* strains (27, 55). Our results are consistent with the previous findings obtained with Δ*gtf1* (35) and indicate that, in the presence of the sole innate immunity, the loss of 6-d-Tal in GPL translates into increased pathogenesis and lethal infection in zebrafish embryos.

**Figure 8:**
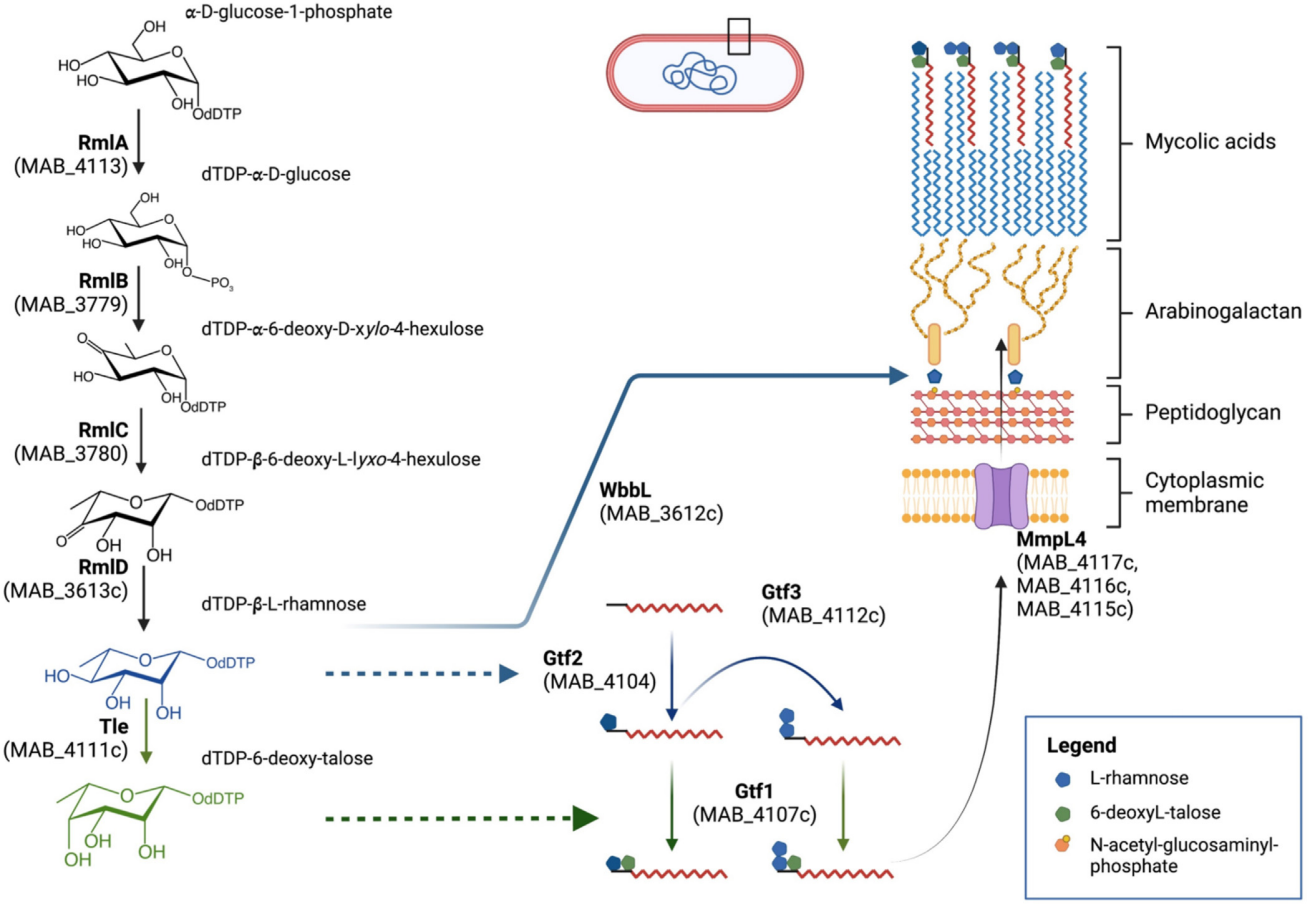
**The biosynthetic interconnections between the arabinogalactan and glycopeptidolipid pathways in *M. abscessus*.** dTDP-L-Rha is generated by the successive action of the Rml pathway enzymes, consisting of RmlA (MAB_4113), RmlB (MAB_3779), RmlC (MAB_3780), and RmlD (MAB_3613c). This dTDP-L-Rha is used by WbbL (MAB_3612c) to produce the Rha-N-acetyl-glucosaminyl-phosphate disaccharide unit that cross-links arabinogalactan to peptidoglycan. In addition, dTDP-L-Rha is also used by Gtf2 (MAB_4104) and Gtf3 (MAB_4112c) for GPL rhamnosylation (*blue arrows*). dTDP-6-d-Tal, produced from dTDP-L-Rha by the dTDP-Rha-4-epimerase (MAB_4111c/Tle) serves as the substrate for the talosyltransferase Gtf1 (*green arrows*), which adds one 6-d-Tal unit to mono- or di-rhamnosylated GPL precursors. The glycosylated GPL (GPL-2a and GPL-3) are then transported across the inner membrane by the MmpL4 complex (MAB_4116c, MAB_4115c, MAB_4117c) and inserted into the outer leaflet of the mycomembrane in the *M. abscessus* S variant.

Overall, this study also emphasizes the biosynthetic interconnection between the arabinogalactan and GPL pathways in *M. abscessus*, as depicted in Figure 8. The mycobacterial cell wall consists of a mycolic acid layer that is bound to peptidoglycan *via* the polysaccharide arabinogalactan. Arabinogalactan is covalently bound to the peptidoglycan *via* the α-L-rhamnopyranosyl-(1→3)-α-D-*N*-acetyglucosaminosyl-1-phosphate unit (56). On the one hand, the donor dTDP-Rha, used by the rhamnosyltransferase WbbL to produce the rhamnosyl-containing linker unit (57), is synthesized by four enzymes (RmlA-D) beginning with dTTP and Glc-1-phosphate. TDP-Rha biosynthesis is essential for the growth of mycobacteria (58), prompting to the development of dTDP-Rha inhibitors for new tuberculosis therapeutics (59). On the other hand, the donor dTDP-Rha is also used by the rhamnosyltransferases Gtf2 and Gtf3, which add the first and second Rha to the GPL biosynthetic intermediates. That dTDP-Rha stands on the crossroad between the arabinogalactan and GPL pathways is further substantiated by its direct use by Tle, which converts dTDP-Rha into dTDP-6-d-Tal, which is itself used by Gtf1 to talosylate the rhamnosylated GPL intermediates. However, while arabinogalactan (and disaccharide linker unit) is fundamental to the structural integrity of the cell wall and required for mycobacterial viability, this is not the case for the GPL, which are lacking in the R strains of *M. abscessus*. However, the loss of GPL has important consequences on *M. abscessus* pathogenesis and infection outcome, as shown in various animal models (26, 27, 30, 35) and in humans (18, 24).

## Experimental procedures

### Mycobacterial strains, growth conditions, and reagents

*M. abscessus* CIP104536^T^ (S) and CIP104536^T^ (R) strains used in this study were previously sequenced and their genomic differences identified and characterized (13, 60). All bacterial strains and plasmids used for this study are listed in Tables S2 and S3, respectively. Mycobacteria were grown in Middlebrook 7H9 broth (BD) supplemented with 0.025% tyloxapol and 10% oleic acid-albumin-dextrose-catalase (OADC) enrichment (7H9^OADC^) or on Middlebrook 7H10 agar (BD) containing 10% OADC (7H10^OADC^) at 37 °C, with antibiotics when required. A Bio-Rad Gene pulser (25 μF, 2500 V, 800 Ω) was used to transform electrocompetent mycobacteria. After transformation, strains carrying the pMV306 (48) derivatives were selected on 250 μg/ml kanamycin.

### Expression of tle in *E. coli*

*MAB_4111c* (*tle*) was PCR-amplified using *M. abscessus* genomic DNA using the specific primers (Table S1, primers 9–10) and Q5 polymerase. The amplicons were cloned into pET30 restricted with KpnI and EcoRI, enabling to introduce the genes in frame with the six-histidine tag. The resulting pET30-*tle* was used for site-directed mutagenesis of specific amino acids involved in the active site (Y181, primers 11–12) or the substrate-binding sites (S121, primers 13–14; N210, primers 15–16; R249, primers 17–18) using the QuikChange II Site-Directed Mutagenesis Kit (Agilent) and based on structural similarity with the *B. thailandensis* dTDP-L-Rha 4-epimerase (UniProt ID: Q2SYH7 WBIB_BURTA). The pET30 constructs containing the native or mutated *tle* genes were used to transform *E. coli* strain BL21 Star DE3 (Life Technologies). Cultures were grown in terrific broth medium containing 50 μg/ml kanamycin at 37 °C under agitation until an optical density at 600 nm (OD600) of 0.7 to 0.9 was achieved. Cultures were placed at 4 °C for 30 min before addition of 1 mM IPTG and an overnight incubation at 16 °C under agitation. Bacteria were collected by centrifugation and pellets were resuspended in lysis buffer (50 mM Tris–HCl pH 8, 400 mM NaCl, 5% glycerol, supplemented with 10 mg/ml of NP-40, 78 μg/ml of DTT, one pill of cOmplete tablets, Mini EDTA-free, EASYpac per 50 ml, 2.175 mg/ml of imidazole, and 1 mg/ml of lysozyme) previously chilled on ice. Bacteria were lysed by sonication and the lysate clarified by centrifugation (12,000 rpm at 4 °C for 30 min), incubated overnight at 4 °C with nickel beads (Ni Sepharose 6 Fast Flow, GE Healthcare), washed using washing buffer I (50 mM Tris–HCl pH 8, 350 mM NaCl, 5% glycerol, 78 μg/ml of DTT, one pill of cOmplete tablets, Mini EDTA-free, EASYpac per 50 ml, and 1.3 mg/ml of imidazole) and in washing buffer II (50 mM Tris–HCl pH 8, 200 mM NaCl, 5% glycerol, 78 μg/ml of DTT, one pill of cOmplete tablets, Mini EDAT-free, EASYpac per 50 ml and 1.3 mg/ml of imidazole). Proteins were eluted in elution buffer (50 mM Tris pH 7, 100 mM NaCl, and 5% glycerol, and 17 mg/ml of imidazole) and then dialyzed overnight at 4 °C in dialysis buffer (50 mM Tris–HCl pH7, 200 mM NaCl, and 5% glycerol).

### Enzymatic activity assay

A 50 μl reaction mixture (in 50 mM of PBS buffer pH 7.5) containing 10 mM of dTDP-L-Rha, 1 mM of NADP^+^, and Nickel-beads eluted fractions enriched with the various MAB_4111c protein variants (0.42 μg/μl MAB_4111c, 0.31 μg/μl MAB_4111c-Y181A, 0.86 μg/μl MAB_4111c-S121A, 0.70 μg/μl MAB_4111c-N210A, 0.75 μg/μl MAB_4111c-R249A, and 0.69 μg/μl MAB_4111c-R249A/N210A/S121A) was incubated at 37 °C for 1 h. The reaction was stopped by diluting the reaction with cooled buffer of acetonitrile/100 mM aqueous ammonium acetate pH 4.5 (60% acetonitrile). As a control reaction, a similar assay was performed by replacing dTDP-L-Rha with 10 mM of dTDP-D-Glc. The diluted solution was analyzed by HPLC equipped with UV detector at 254 nm using ZIC-cHILIC column. The column was eluted at 40 °C with acetonitrile/100 mM aqueous ammonium acetate pH 4.5 (60% acetonitrile) at a flow rate of 0.6 ml/min. The enzyme activity was determined by the conversion of the product (Yield = [dTDP-6-deoxy-L-Tal]/([dTDP-6-deoxy-L-Tal] + [dTDP-L-Rha])). To analyze the activity by NMR, a reaction mixture consisting of 500 μl deuterium oxide (D_2_O) containing 10 mg of dTDP-L-Rha, 5 mg of NADP^+^, 5 mg of disodium hydrogen phosphate (Na_2_HPO_4_), and enriched MAB_4111c protein variants (0.14 μg/μl MAB_4111c, 0.13 μg/μl MAB_4111c-Y181A) was incubated at 37 °C for 5 h.

For the time-curve analysis, a 100 μl reaction mixture (in 50 mM of PBS buffer, pH 7.5) containing 10 mM of dTDP-L-Rha, 1 mM of NADP^+^, and 0.42 μg/μl of MAB_4111c was incubated at 37 °C for different time point. The reaction was stopped by diluting the reaction with cooled buffer of acetonitrile/100 mM aqueous ammonium acetate pH 4.5 (60% acetonitrile). The diluted solution was analyzed by HPLC equipped with UV detector at 254 nm using ZIC-cHILIC column. The column was eluted at 40 °C with acetonitrile/100 mM aqueous ammonium acetate pH 4.5 (60% acetonitrile) at a flow rate of 0.6 ml/min. All dTDP-activated sugar nucleotides were produced enzymatically, as reported previously (61).

LC-MS/MS analysis was performed on a triple quadrupole mass spectrometer LCMS8060XS (Shimadzu) coupled with a Nexera system (Shimadzu). Chromatography was performed on a ZIC-cHILIC column as previously described (2.1 mm i.d. × 150 mm, 1.7 μm; SeQuant) (62, 63). The mobile phases were as follows: (A) 20 mM acetate buffer (pH 4.5) containing 90% acetonitrile and (B) the same buffer. The elution gradient was as follows: 12 to 18% linear gradient of buffer B for 12 min; 18 to 25% linear gradient of buffer B for 5 min; 25 to 55% buffer B for 5 min; and 55% buffer B for 5 min. The flow rate was maintained at 0.2 ml/min. Analysis of dTDP-rhamnose and dTDP-d-Tal was performed in the MRM mode using the specific precursor ion [M-H]^−^ at *m/z* 547.1 and product ion pairs at *m/z* 321.1. Product ion scanning was also conducted by MS/MS analysis of the precursor ion [M-H]^−^ at *m/z* 547.1.

### Structural modeling of tle using AlphaFold 3

The dimeric structure of Tle was predicted using AlphaFold 3 (43). Two identical sequences of Tle, each comprising 353 amino acids, along with two copies of the NAD^+^ ligand, were input into the AlphaFold 3 web server (https://alphafoldserver.com/). The server produced five model outputs. The model with the highest pLDDT score was selected for further analysis. The pLDDT score ranges from 0 to 100, where 0 indicates the lowest confidence and 100 the highest confidence in the structural prediction. Additionally, for multimeric targets, AlphaFold 3 provides predicted template modeling and interface predicted template modeling scores, both ranging from 0 to 1, indicating the confidence in the predicted oligomeric state of the protein.

### Deletion of *tle* and complementation in *M. abscessus*

The deletion mutant was generated in the S morphotype of the reference strain CIP104536^T^ (60) using the suicide vector pUX1-*katG* by double homologous recombination (47). Briefly, the upstream and downstream gene regions were PCR-amplified using the primers listed in Table S1 and ligated into the PacI/NheI-linearized pUX1-*katG*. After transformation, bacteria were selected on 7H10^OADC^ supplemented with 250 μg/ml kanamycin with a visual screening of red fluorescent colonies, which have undergone the first homologous recombination. The second homologous recombination event was induced by isoniazid counter-selection and selected on 7H10^OADC^ with 50 μg/ml isoniazid and screening for nonfluorescent colonies and susceptibility to kanamycin (35, 47). Complementation was performed by introducing a complementation plasmid generated using the integrative pMV306 (Table S3). Genes in fusion with a super folder green fluorescent protein-tagging sequence were amplified by PCR under the control of the endogenous promoter. Proper gene deletion and all plasmids were verified by PCR and DNA sequencing.

### Western blotting

Fifty millilitres of cultures were grown in minimal salt medium (1 g/L KH_2_PO_4_, 500 mg/L NaCl, 2 g/L Na_2_HPO_4_, 1 g/L NH_4_Cl, 200 μM CaCl_2_, 2 mM MgSO_4_, 5% glycerol, and 0.02% tyloxapol). The bacterial pellet was washed twice with PBS supplemented with 0.025% tyloxapol (v/v) and resuspended in PBS supplemented with cOmplete protease inhibitors cocktail (Roche, Sigma Aldrich) and disrupted by bead beating using 1 mm diameter glass beads and a Mixer Mill MM 301 (Retsch) for two pulses of 3 min at 30 Hz. Protein concentration was determined using the BCA Protein Assay Reagent kit (Thermo Fisher Scientific), according to the manufacturer’s instructions. Equal amounts of proteins (20 μg) were separated by 12% SDS-PAGE, transferred onto a nitrocellulose membrane, probed for 1 h with either mouse anti-GFP (dilution 1:1000; Sigma) (antibodies-online GmbH) or mouse anti-Antigen 85 (dilution 1:20; loading control) antibodies. Membranes were washed with PBS supplemented with 0.02% Tween20 (w/v) and incubated for 45 min with goat anti-mouse antibody conjugated to HRP (dilution 1:5000; Abcam). Bands were revealed using a SuperSignal West Femto (Thermo Fisher Scientific) and a ChemiDoc MP system (Bio-Rad laboratories). For detection of the MAB_4111c variants produced in *E. coli*, the membrane was probed with anti-His mouse monoclonal antibodies (dilution 1:1000) and incubated with HRP-labeled goat anti-mouse IgG (H+L) (dilution 1:2000).

### Growth, colony morphology, biofilm formation, and sliding motility

Growth curves were established based on cultures grown for 72 h and diluted in 7H9^OADC^ to reach an initial OD_595 nm_ of 0.05 and dispensed in flat-bottom 96-well microtiter plates (200 μl per well). Plates were statically incubated at 37 °C and measurements were taken on a daily basis using a spectrophotometer multimode microplate reader (Tecan Spark 10M, Tecan Group Ltd).

Colony morphology was assessed from 72 h cultures in 7H9^OADC^ and streaked on 7H10^OADC^. Plates were incubated at 37 °C for 5 days and photographed using a Zeiss microscope equipped with a Zeiss Plan Neo Fluor Z13/0.25 FWD objective and an Axiocam503 monochrome camera (Zeiss). Images were processed using ZEN 2 (Blue Edition).

*M. abscessus* biofilm formation was performed following a modification of the colony-biofilm model previously described (64). Briefly, *M. abscessus* cultures were grown in Middlebrook 7H9^OADC^, 0.2% glycerol, and tyloxapol (0.025%) at 37 °C and 80 rpm for 72 h. Cultures were centrifuged at 3500 rpm for 5 min, washed two times with PBS, and then diluted to an OD_600 nm_ of 0.5 (∼1.5 × 10^8^ CFU/ml). White black, polycarbonate membranes (diameter, 13 mm; pore size 0.2 μm, Whatman, Merck) were placed on 7H10^OADC^ and inoculated with 10 μl of the bacterial suspension. The membrane-supported biofilms were statically incubated for 5 days at 37 °C. Afterwards, the membrane-supported biofilms were imaged and processed using a ZEISS Axio Zoom V16 binocular equipped with a lighting device (Zeiss HXP 200C Zeiss) at 7× zoom, detecting the gray level. On biofilm fluorescence pictures, a straight line was drawn (covering all the diameter of the biofilm-colony) and gray-level intensity (GLI) of each pixel as retrieved to draw GLI spectra using ZEN 2 software (Blue Edition). The colony volume was calculated by using the half volume of an ellipsoid: Colony volume = 2/3 × π × GLIM × (CD/2)^2^, where GLIM is the GLI maximum and CD is the colony diameter in pixels. After taking pictures, each membrane-supported biofilm was processed for quantifying the number of CFU/membrane. Colony-biofilm density was estimated as a coefficient resulting of CFU/membrane and the colony volume.

Sliding motility of each strain was evaluated using a modified methodology previously described (28). For each strain, 10 μl of 24 h cultures (OD_600 nm_ = 0.9–1.0) were dropped in the center of well from a 6-well containing 7H9 broth with 0.3% agar and without adding any carbon source. The sliding distance was evaluated and measured after incubation for 10 days at 37 °C under humidified conditions. This experiment was performed by using three biological replicates and two technical replicates.

### Hydrophobicity assay

Mycobacterial surface hydrophobicity was determined following the methodologies developed previously (49). Each strain was grown in Middlebrook 7H9^OADC^ at 37 °C for 72 h, and then some colonies were resuspended and sonicated in 5 ml of 0.9% NaCl in Pyrex 16 × 100 mm disposable round-bottom threaded culture tubes (Corning). The OD_595 nm_ (A_0_) of 200 μl was measured using spectrophotometer multimode microplate reader (Tecan Spark 10M). Subsequently, 2 ml hexadecane (Sigma Aldrich) were added to the glass tubes, and the mixtures were vortexed for 30 s. After phase separation (∼20 min), the OD_595 nm_ of 200 μl was measured using a multimode microplate reader. Subsequently, the aqueous phase (A_1_) was measured again and compared to the organic phase. The percentage of cell surface hydrophobicity (H) of each strain immersed in the organic hexadecane phase was calculated using the equation H (%) = [(A_0_ − A_1_)/A_0_] × 100. This experiment was performed by using three biological replicates and three technical replicates.

### Transmission electron microscopy

Seventy two hours cultures were washed with PBS, immersed overnight at 4 °C in PHEM buffer containing 2.5% glutaraldehyde (pH 7.4), rinsed in PHEM buffer, and post-fixed in 0.5% osmium acid and 0.8% potassium ferricyanide trihydrate for 2 h at room temperature and in the dark. After washing twice with PHEM buffer, mycobacteria were dehydrated in a graded series of ethanol solutions (30–100%) and embedded in EmBed 812 using an Automated Microwave Tissue Processor for electron microscopy (Leica). Seventy nanometer sections were cut at different levels of each block/stem using a microtome (Leica-Reichert Ultracut E, Leica), counterstained in 70% ethanol with 1.5% uranyl acetate and lead citrate, and observed using a Tecnai F20 TEM at 120 KV.

### Drug susceptibility testing

Antimicrobial susceptibility was tested using broth microdilution following the European Committee on Antimicrobial Susceptibility Testing (EUCAST) (65). For this purpose, 96-well RAPMYCOI Sensititre titration plates (Thermo Fisher Scientific) were used following the manufacturer’s recommendations.

### Phylogenetic analysis of *tle* orthologous genes

Ortholog genes of *tle* in the genus *Mycobacterium* were found using BLASTp (66) against the NCBI nonredundant (nr) database by limiting the search to mycobacteria (taxid: 85,007) with a full bacterial scientific name registered and extending the maximum target sequences up to 1000. In case of having more than one time the same mycobacterial species name, only the one with the highest identity percentage was considered. All the proteins related to the Rml pathway were excluded from the analysis. Orthologs of *tle* in other bacteria (nonmycobacteria) were found in a manner similar to that described above but excluding the search to Mycobacteria (taxid: 85,007) and limiting the maximum target sequences to 100. The Molecular Evolutionary Genetics Analysis (MEGA) version 11 software (67) was used to generate a ClustalW multiple sequence alignment of all the sequence and to construct Neighbour-Joining tree. The obtained tree was redrawn using TreeViewer Version 2.1.0 (68) and colored with Inkscape (https://inkscape.org). To structurally validate the findings from the phylogenetic tree, we analyzed the presence of GPL and their glycosidic composition in 14 clinical NTM isolates identified with Genotype *Mycobacterium* CM or AS (Hain Lifescience) from the Department of Clinical Microbiology of the University Hospital Fundación Jiménez Díaz.

### Lipid extraction and analysis

Rapid-growing and slow-growing mycobacteria were grown on 7H10^OADC^ agar plates for 72 h and in 7H9^OADC^ broth for at least 14 days, respectively. After growing, pellets were collected and lyophilized, then 25 to 50 mg of bacterial pellets were subjected to apolar lipid extractions using petroleum ether (69). GPL were extracted from the polar fraction using chloroform/methanol/0.3% NaCl (9/10/3, v/v/v) and then with chloroform/methanol/0.3% NaCl (5:10:4, v/v/v). The combined solvent extracts were mixed for 5 min with chloroform and 0.3% NaCl (1:1, v/v) and centrifuged at 3000*g* for 5 min to separate the lower organic phase from the aqueous phase. The upper aqueous layer was discarded, and the lower organic phase was evaporated under a stream of nitrogen and resuspended in chloroform/methanol (2:1, v/v) for TLC analysis or itol-acetates derivation.

#### TLC analysis

Between 50 to 150 μg of each extract were spotted along 5 mm lane on Silica gel 60 F254 plates (Merck). GPL were separated at 4 °C using chloroform/methanol/water (90:10:1, v/v/v). More polar lipids were separated using chloroform/methanol/water (65:25:4, v/v/v). Glycolipids were revealed by spraying the plates with orcinol in 20% sulfuric acid and charring.

#### Glycolipids saponification

500 μg of the polar lipid fraction were dried under nitrogen. Then, 1 ml of 0.1 M sodium hydroxide in chloroform/methanol (1:1, v/v) was added and heated overnight at 37 °C. One mL of butanol and 1 ml of water were added and the mixture vortexed for 1 min. After centrifugation for 30 s, the upper butanolic phase was dried under a nitrogen stream and dissolved in 300 μl of chloroform/methanol (2:1, v/v).

#### Itol-acetates derivation

After addition of 1 ml of 4 M TFA, the sample was heated for 4 h at 100 °C, dried, and desiccated overnight. The monosaccharides were reduced for 4 h at room temperature in 500 μl NaBH_4_ 10 mg/ml in 2 M NH_4_ and stopped with concentrated glacial acetic acid. Samples were dried at 55 °C under a nitrogen stream by codistillation with methanol five times, desiccated overnight. Samples were peracetylated in 500 μl anhydride acetic for 4 h at 80 °C. The reaction products were washed five times with chloroform/water (1:1, v/v). The chloroform-rich phase was then filtered, dried, and dissolved in 500 μl chloroform. For GC/FID, 1 μl of itol-acetate derivatives was injected in splitless mode with an automatic sampler on a Solgel 1 MS 30 m × 0.25 mm × 0.25 μm capillary column with the following gradient temperature: 120 to 230 °C at 3 °C/min, then to 270 °C at 10 °C/min. Compounds were detected either with a flame ionization detector or a single quadrupole (Agilent Technologies). Previously determined retention times of differentially methylated Rha and unmodified 6-d-Tal were used for identification (33) while electronic impact mass spectrometry was used for methylated 6-d-Tal determination.

#### MALDI-TOF mass spectrometry

Before spotting 1 μl on the MALDI plate, 10 μl of 20 mg/ml dihydroxybenzoïc acid in chloroform/methanol (1:2, v/v) were mixed with 5 μl of the sample extract in chloroform/methanol (2:1, v/v). MS positive and negative spectra were acquired on an Axima Resonance (Shimadzu) in reflectron mode.

### Zebrafish maintenance

Zebrafish (*Danio rerio*) were kept and handled in compliance with the guidelines of the European Union for handling laboratory animals and approved by the Direction Sanitaire et Vétérinaire de l’Hérault for the ZEFIX-CRBM zebrafish facility (Montpellier) (registration number C-34-172-39). All experiments were approved by Le Ministère de l’Enseignement Supérieur et de la Recherche under the reference APAFIS#24406 to 2020022815234677 V3. Eggs were obtained by natural spawning, quickly bleached, and incubated at 28.5 °C in Petri dishes containing E3 medium (5 mM NaCl, 0.17 mM KCl, 0.33 mM CaCl_2_, 0.33 mM MgSO_4_). The transgenic reporter line Tg(*mpx:eGFP*)^*il14*^ (70) was used in this study.

### Zebrafish infection

At 24 h post-fertilization (hpf), embryos were enzymatically dechorionated using 1 mg/ml of pronase (stock of 5 mg/ml diluted in E3) and placed in 60 mm Petri dishes containing E3 at 28.5 °C. Microinjection was performed as previously described (71). Embryos were injected in the caudal vein with ±250 CFU of either the R, S, SΔ*tle*, carrying the pMV306-mScarlet, SΔ*tle::C* carrying the pTEC27-tdTomato, or the PBS control. Injected embryos were then rinsed twice and transferred individually in 48-well plates containing E3 and randomized for survival assays and CFU counts. Inoculum was checked by microinjecting a PBS drop and plating it on 7H10^OADC^. For survival assay, embryos were monitored every 24 h for 12 days and marked as dead in the absence of a heartbeat. Bacterial burden was determined at 2 dpi, 3 dpi, and 5 dpi as following: embryos were individually manually disrupted with pellet pestles in 50 μl of PBS supplemented with 0.025% of tyloxapol. Lysates were sonicated for 30 s after adding 50 μl of PBS supplemented with 2% of Triton-X-100. Serial dilutions were plated on LB agar plates containing rifampicin (15 μg/ml) and kanamycin (250 μg/ml) and incubated for 4 days at 37 °C.

For zebrafish imaging, embryos were anesthetized with 0.02% buffered MS222 and immobilized with 0.5% low melting agarose in a lateral position. Images were acquired with an EVOS M7000 Imaging System at 3 dpi and 5 dpi with the Olympus 2× Objective PlanApo 0.08 NA and the EVOS 10× Objective 0.3NA using the EVOS Light Cube, GFP 2.0. After imaging, embryos were rinsed and transferred back the 48-well plate with fresh E3. Image processing was done using ZEISS ZEN3.7 software.

### Statistical analyses

All analyses were performed using R commander (https://socialsciences.mcmaster.ca/jfox/Books/RCommander/) (72) and/or GraphPad Prism version 9.0.0 for Windows (GraphPad Software). The normality of data was evaluated using a Shapiro–Wilk test. Descriptive data are cited as median and interquartile range in case of non-normal distribution for each of the variables were calculated. A nonparametric Kruskal–Wallis test was used to compare more than three groups and a Wilcoxon test was used to compare two groups. Zebrafish survival assays are represented in Kaplan-Meier graphs and analyzed with a Log rank test. For determination of bacterial counts, CFU were log_10_ transformed and the significance between multiple selected groups was determined using one-way ANOVA with Šidák’s Multiple Comparisons test after validating the normality of the data.

## Data availability

All data are contained within the manuscript and the Supporting Information. The raw data can be shared upon request.

## Supporting information

This article contains supporting information (47, 48, 73, 74, 75, 76, 77).

## Conflict of interest

The authors declare that they have no conflicts of interests with the contents of this article.
