# Supplementary material for: A dTDP-L-rhamnose 4-epimerase required for glycopeptidolipid biosynthesis in Mycobacterium abscessus
Source: J Biol Chem. 2024 Oct 1;300(11):107852. doi: 10.1016/j.jbc.2024.107852 (PMC11549994; doi:10.1016/j.jbc.2024.107852)
Supplement: Supporting Information [file mmc1.docx]

**SUPPORTING INFORMATION**

**A dTDP-L-Rhamnose 4-epimerase required for glycopeptidolipid biosynthesis**

**in *Mycobacterium abscessus***

John Jairo Aguilera-Correa^1^, Fangyu Wei^3,$^, Louis-David Leclercq^4,5,$^, Yara Tasrini^1,$^, Edukondalu Mullapudi^6^, Wassim Daher^1,2^, Kazuki Nakajima^7^, Stéphane Canaan^8^, Jean-Louis Herrmann^5^, Matthias Wilmanns^6,9^, Yann Guérardel^4,7^, Liuqing Wen*^3^,* and Laurent Kremer^1,2*^

^1^Centre National de la Recherche Scientifique UMR 9004, Institut de Recherche en Infectiologie de Montpellier (IRIM), Université de Montpellier, 1919 route de Mende, 34293, Montpellier, France.

^2^INSERM, IRIM, 34293 Montpellier, France.

^3^Shanghai Institute of Materia Medica, Chinese Academy of Sciences, Chinese Academy of Sciences, 555 Zuchongzhi Rd, 201203, Shangai, China

^4^Univ. Lille, CNRS, UMR 8576 - UGSF - Unité de Glycobiologie Structurale et Fonctionnelle, F-59000 Lille, France.

^5^Université Paris-Saclay, UVSQ, Inserm, Infection et inflammation, 78180, Montigny-Le-Bretonneux, France.

^6^European Molecular Biology Laboratory, Hamburg Unit, Notkestrasse 85, 22607 Hamburg, Germany.

^7^Institute for Glyco-core Research (iGCORE), Gifu University, Gifu, Japan

^8^Aix-Marseille Univ, CNRS, LISM, IMM FR3479, Marseille, France.

^9^University Medical Center Hamburg-Eppendorf, Martinistrasse 52, 20246 Hamburg, Germany.

^$^Equal contribution

*Corresponding author: Laurent Kremer; Tel: (+33) 4 34 35 94 47; E-mail: laurent.kremer@irim.cnrs.fr

**Running title:** Tle epimerase is required for glycopeptidolipid synthesis

**Keywords:** Mycobacterium abscessus, cell wall, glycopeptidolipid, Rhamnose, 6-deoxy-Talose, epimerase, morphotype, virulence, zebrafish.

**
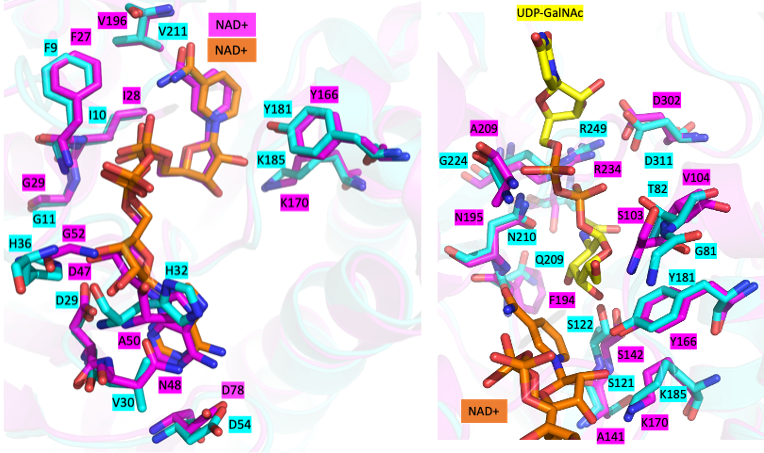

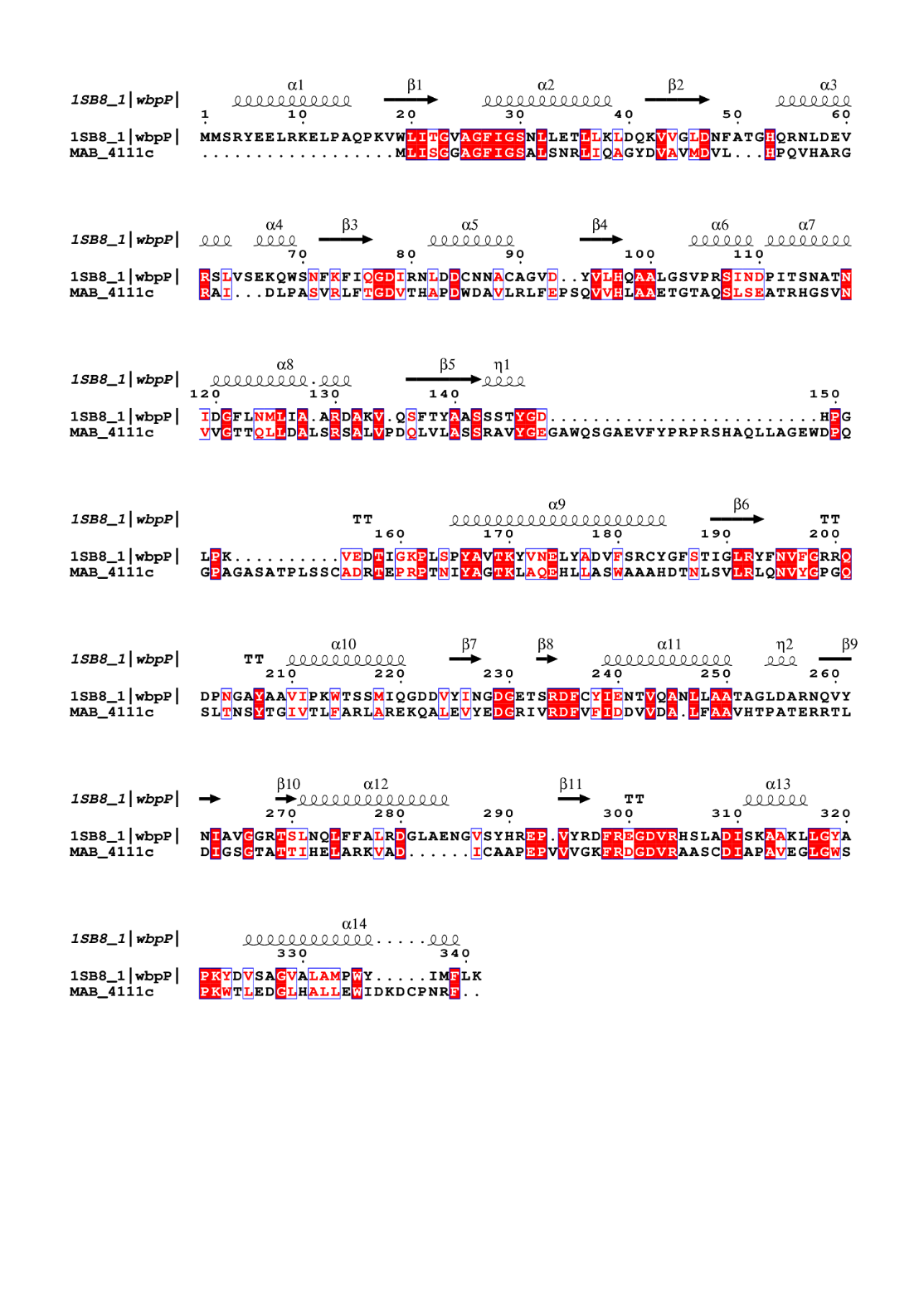

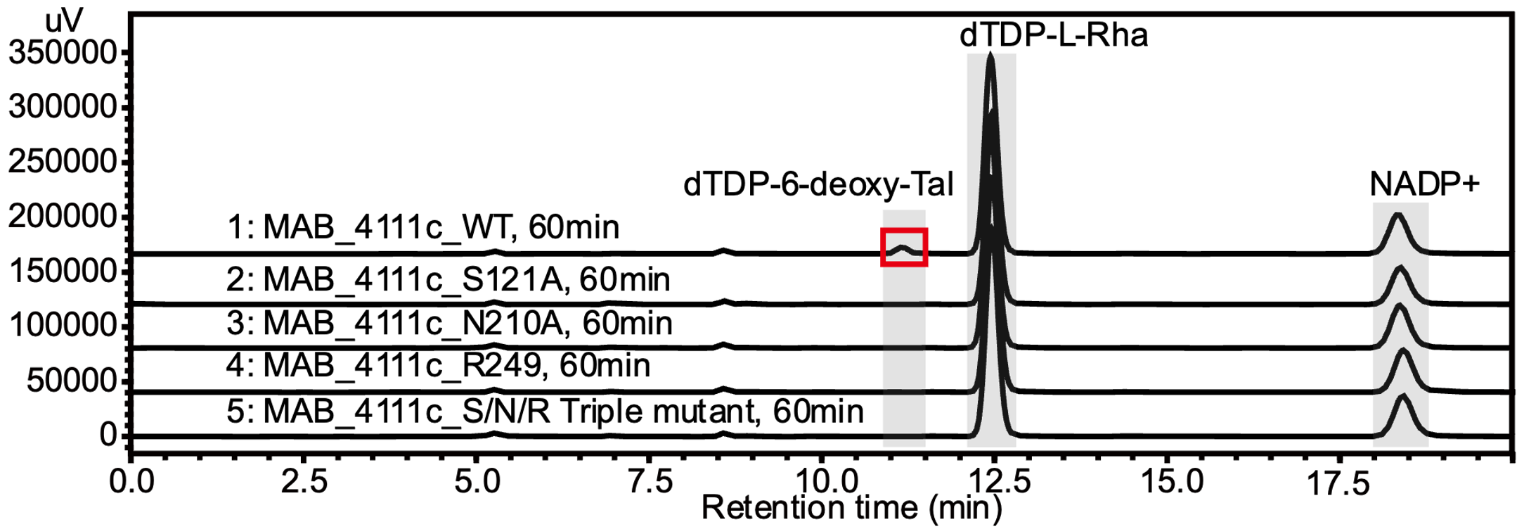
**

**Figure S1. (A)** Structure-based sequence alignment for *Pseudomonas aeruginosa* UDP-*N*-acetylglucosamine 4-epimerase (WbpP) and MAB_4111c (Tle) proteins generated by Muscle a Multiple Sequence Alignment (MSA) webserver and ESPript 3.0. **(B)** Left panel: Overlay of the NAD^+^ binding site from MAB_4111c and WbpP, colored cyan and magenta, respectively. NAD^+^ molecules of Tle and WbpP are shown as orange and magenta stick models, with binding residues highlighted for both proteins. Right panel: Overlay of the substrate-bound WbpP structure with Tle. The substrate UDP-GalNac is colored yellow and shown as a stick representation, with interacting residues highlighted for both structures.

**A**

**B**

kDa


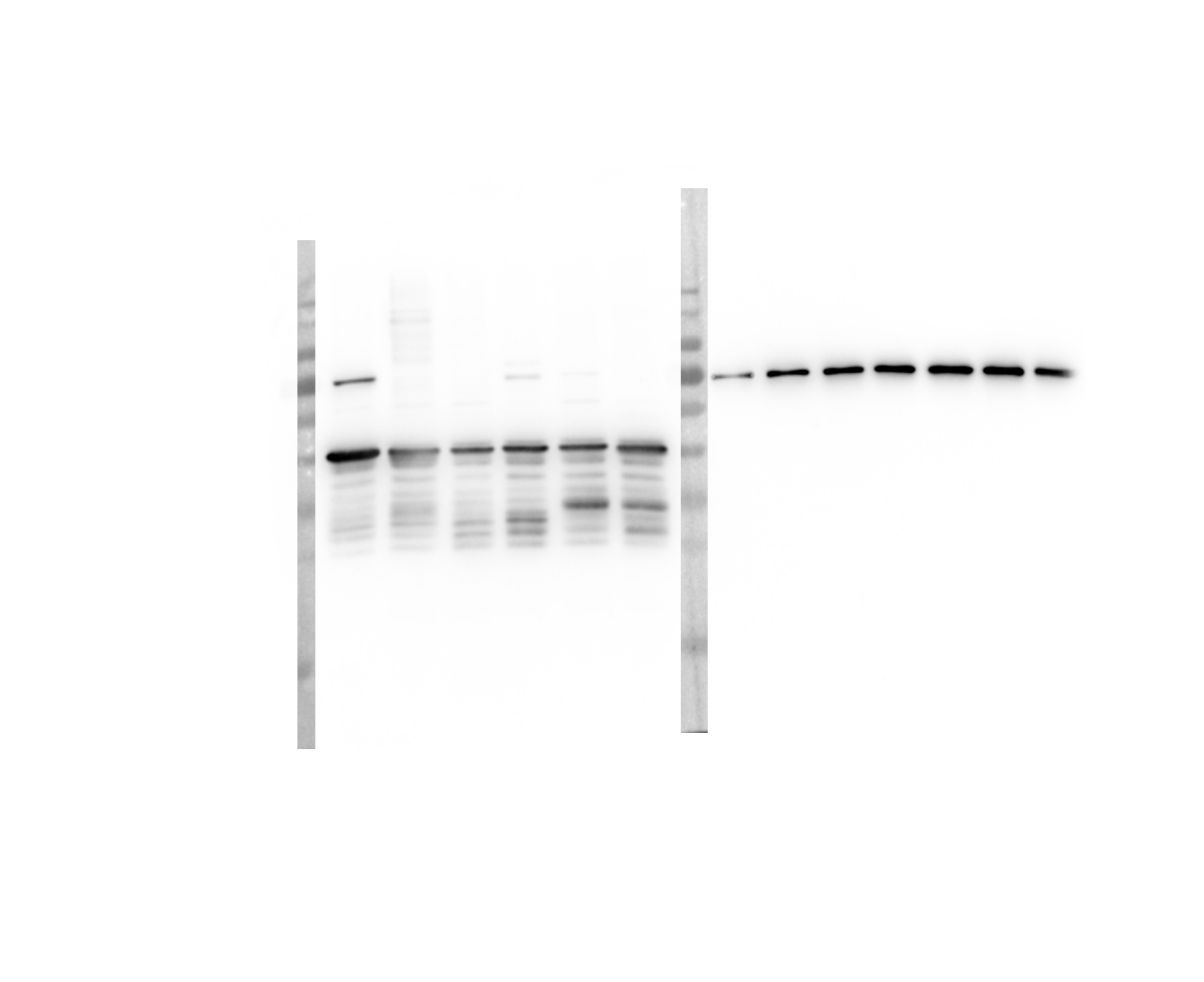


70

55

100

130

180

40

35

MAB_4111c_WT

MAB_4111c_Y181A

MAB_4111c_S121A

MAB_4111c_N210A

MAB_4111c_R249A

MAB_4111c_S/N/R

Triple mutant

MAB_4111c-His

**Figure S2. Loss of activity of MAB_4111c derivatives. (A)** Western blotting showing the presence of the different MAB_4111c variants fused to a His-tag at the C-terminus in eluted fractions after Ni-chromatography of MAB_4111c with point mutations in the predicted catalytic and substrate binding sites (Y181A, S121A, N210A, R249A, and S121A/N210A/R249A triple mutant). Bands were detected using anti-His antibodies. The relative concentration of each protein was determined by comparing the grayscale of the bands using ImageJ and adjusted so that the same quantity of each protein was added to the assay in the presence of NADP^+^ and dTDP-6-d-Tal. **(B)** HPLC analysis of the activity of the MAB-4111c-derived proteins shown in **(A)** and incubated with NADP^+^ and dTDP-Rha. The dTDP-6-d-Tal peak is represented by a red square.

**A**

**B**


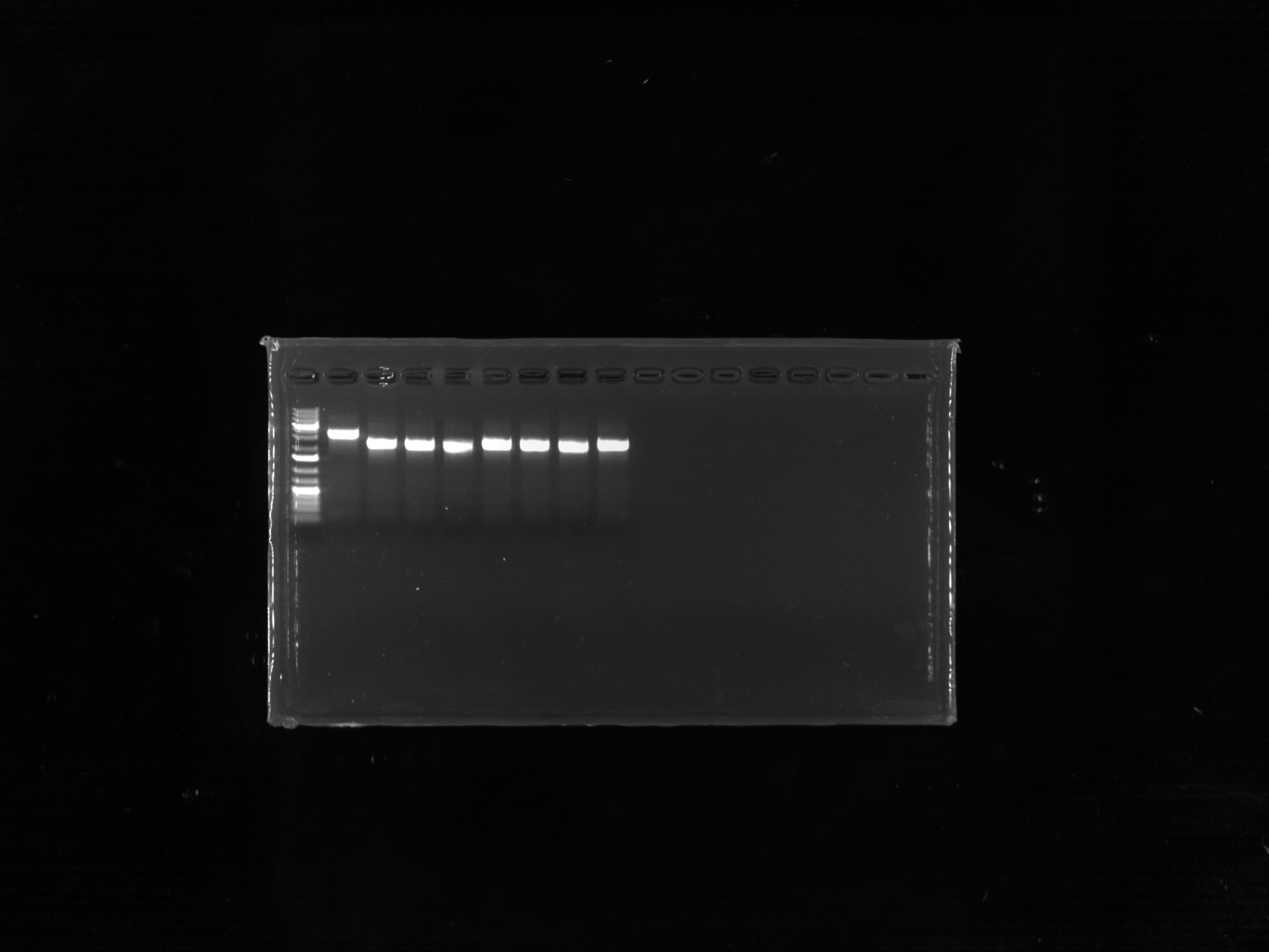


wt

Δ*tle*

5

1.5

0.5

**Size**

(Kbp)

Δ*tle*

kDa

70

55

35

25

Tle-GFP

S

R

Δtle::C


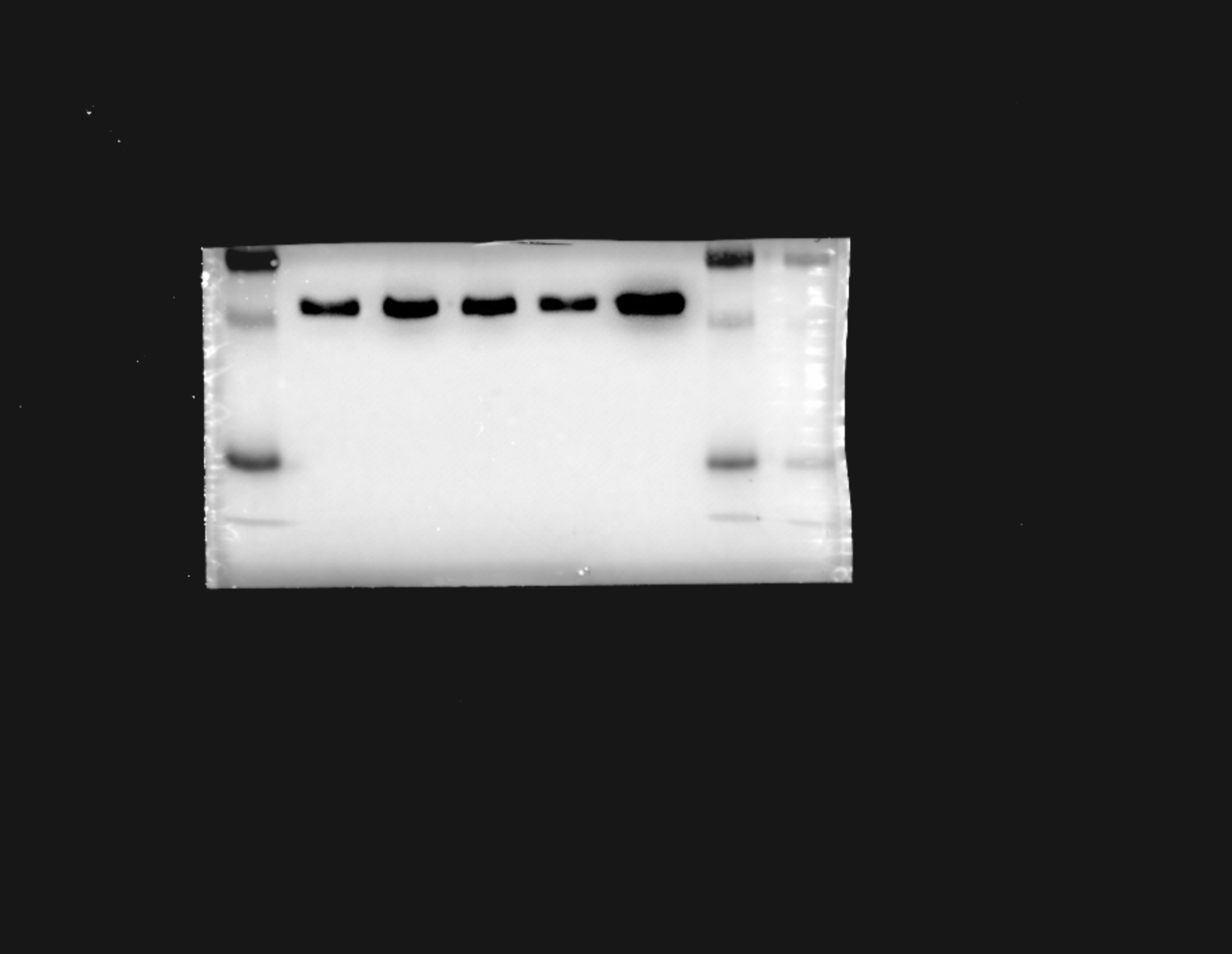


α-Ag85


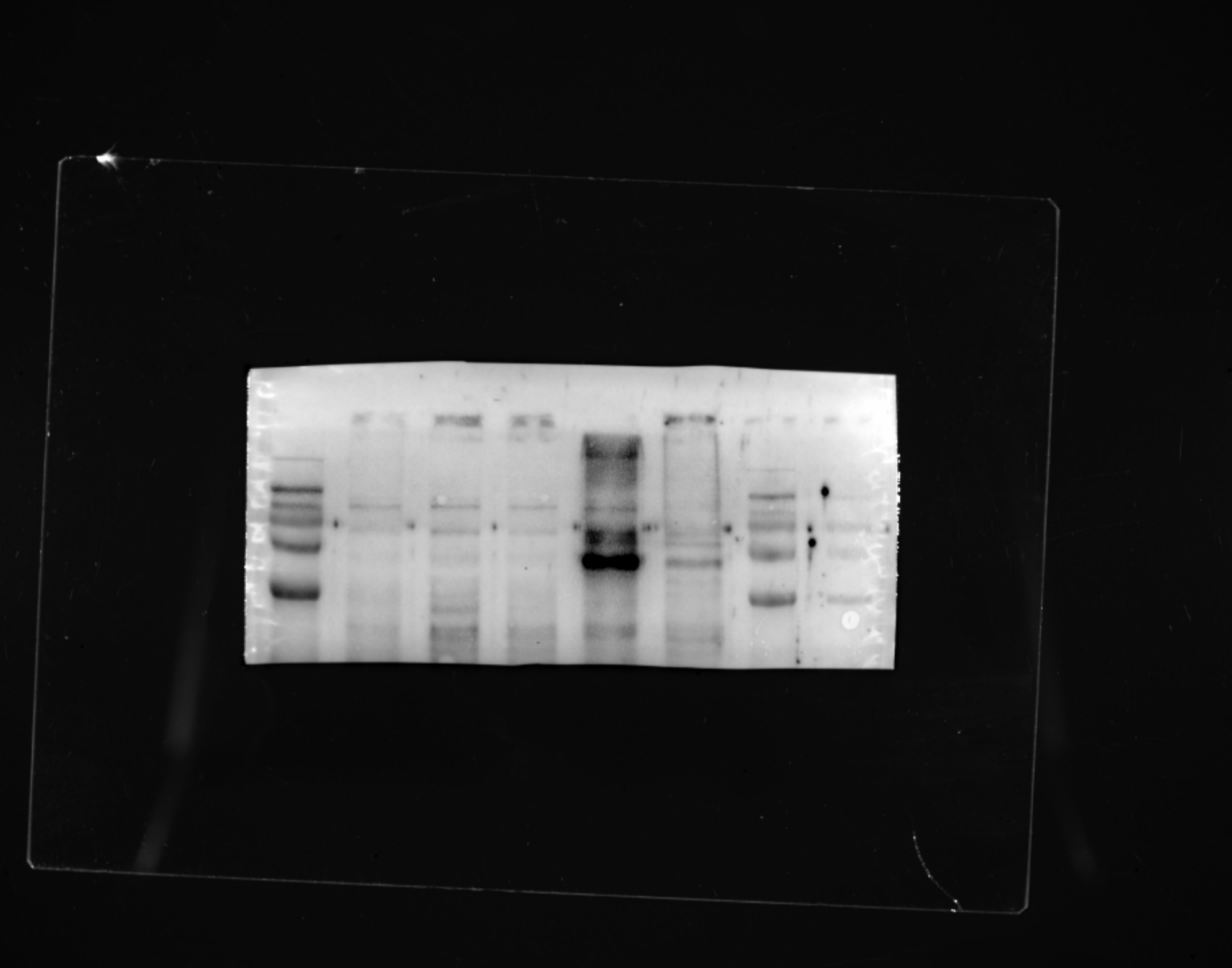


α-GFP


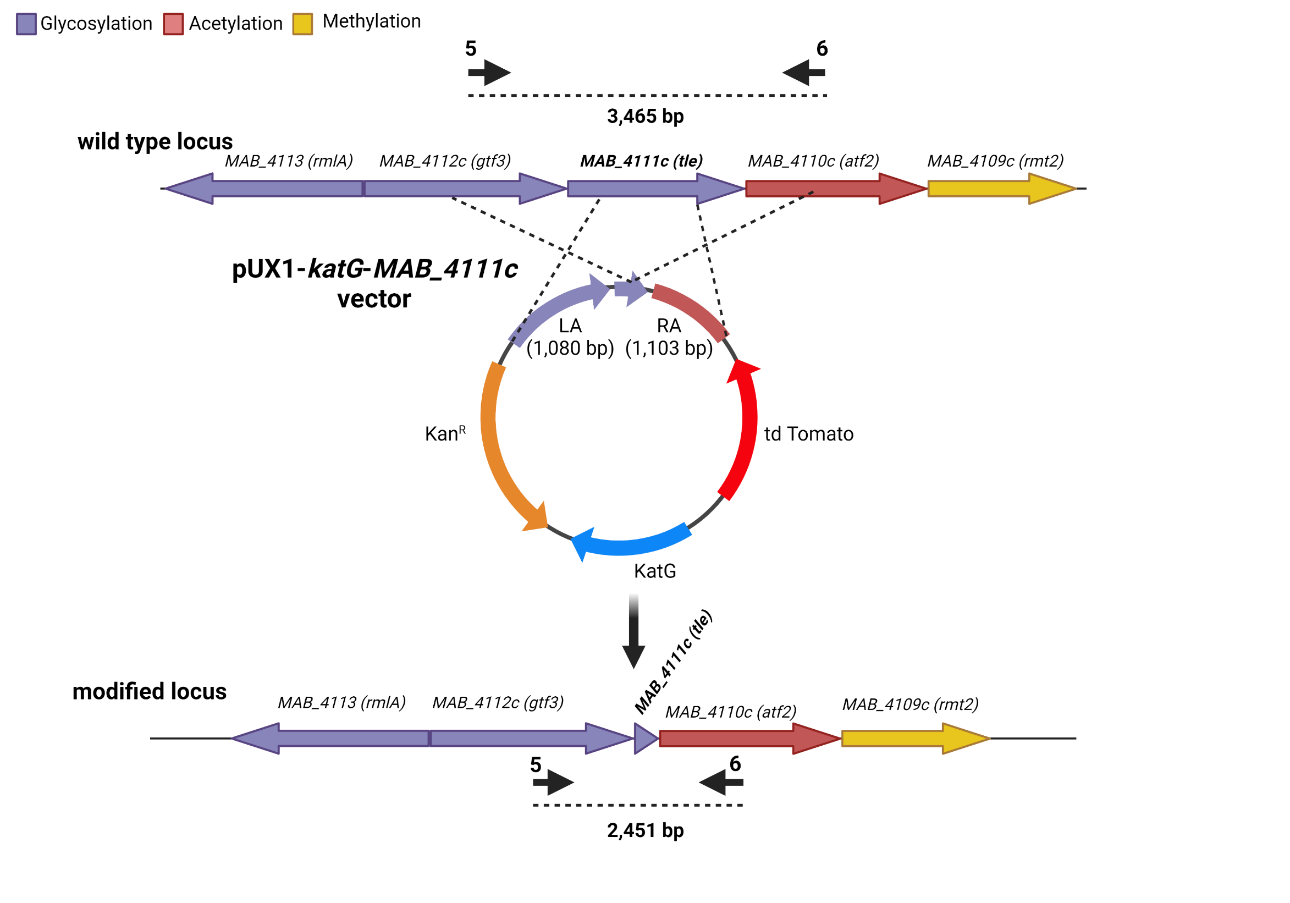


**A**

**C**

Ag85

**Figure S3.** Unlabelled deletion strategy for the deletion of *MAB_4111c (tle)* in *M. abscessus*. **(A)** The *MAB_4111c* gene is located between the *gtf3* and *atf2* genes. The pUX1-katG-*MAB_4111c* plasmid was generated to remove *MAB_4111c* by double homologous recombination. The DNA sequence of the left arm (LA) of *MAB_4111c* (1,080 bp) was amplified by PCR and subcloned into the PacI and MfeI sites of pUX1-katG. The DNA sequence of the right arm (RA) of *MAB_4111c* (1,103 bp) was amplified by PCR and subcloned into the MfeI and NheI sites of pUX1-katG. The resulting suicide plasmid (lacking motifs for episomal replication or mobile elements promoting chromosomal integration) was used to transform *M. abscessus*, replicating only by homologous recombination between the cloned sequences and their chromosomal homologous sequences. The first recombination event was selected in the presence of kanamycin. Single red fluorescent tdTomato-expressing clones were subjected to the second round of recombination selected with isoniazid and analysed for double crossover phenotypes, *i.e.* loss of red fluorescence, sensitivity to kanamycin and resistance to isoniazid. The dashed lines represent the size of the expected PCR products in *M. abscessus* wild-type and Δ*MAB_4111c*. Black arrows represent the primers used for PCR analysis. **(B)** PCR analysis to detect the deletion of *tle* in Δ*MAB_4111c*. Genomic DNA from wild-type bacteria was used to amplify the intact gene. Amplicons were subjected to sequencing to confirm the proper deletion of *tle* in the mutant. **(C)** The Δ*tle* mutant was complemented with the integrative pMV306 carrying the *tle* gene fused to a GFP-tag (Δ*tle::*C). In this complemented strain, the expression of Tle is driven by the endogenous promoter. The Western blot shows the expression of the Tle-GFP protein using anti-GFP antibodies. Antigen 85 (Ag85) antibodies were included as a loading control.

**B**


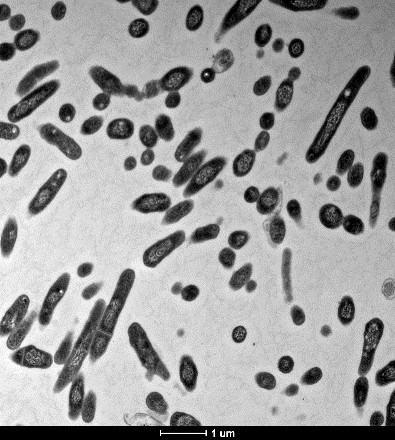

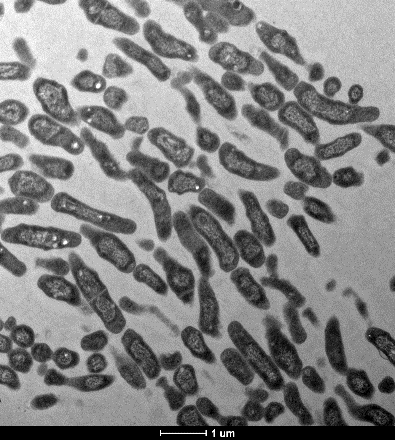

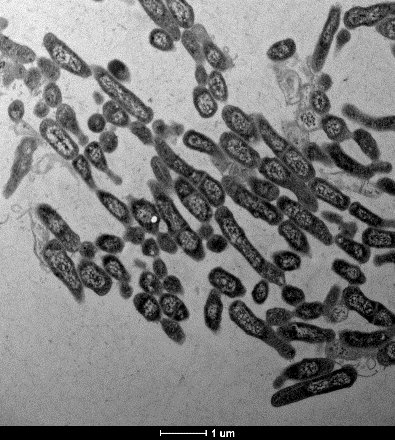

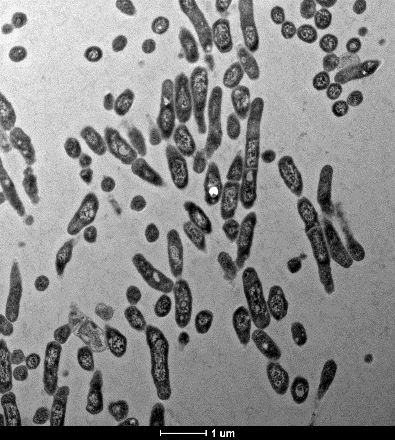


**S**

**R**

**Δ*tle***


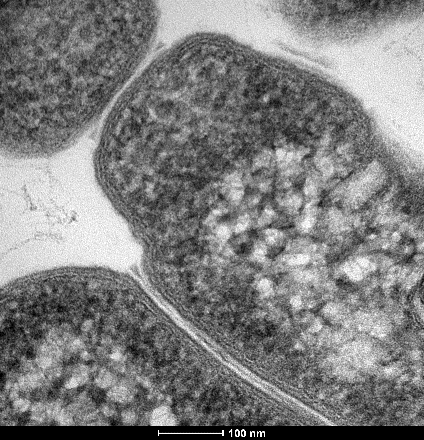

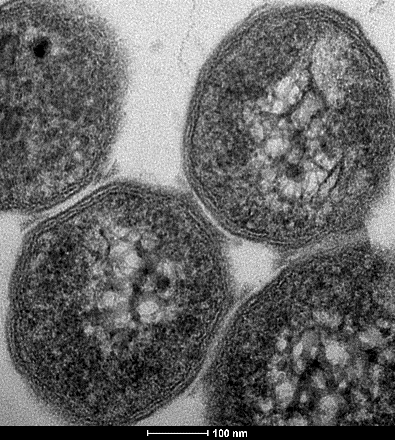

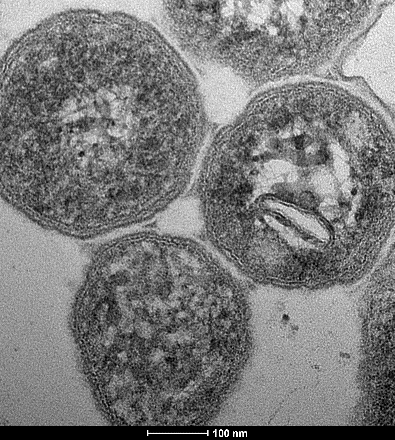

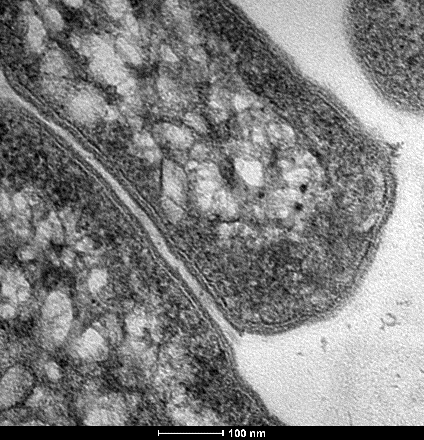


**S**

**R**

**Δ*tle***

**Δ*tle*::C**

**Δ*tle*::C**

**Figure S4.** TEM micrographs of each strain evaluated in this study. Red and blue bars represent 2 µm and 100 nm, respectively.

**
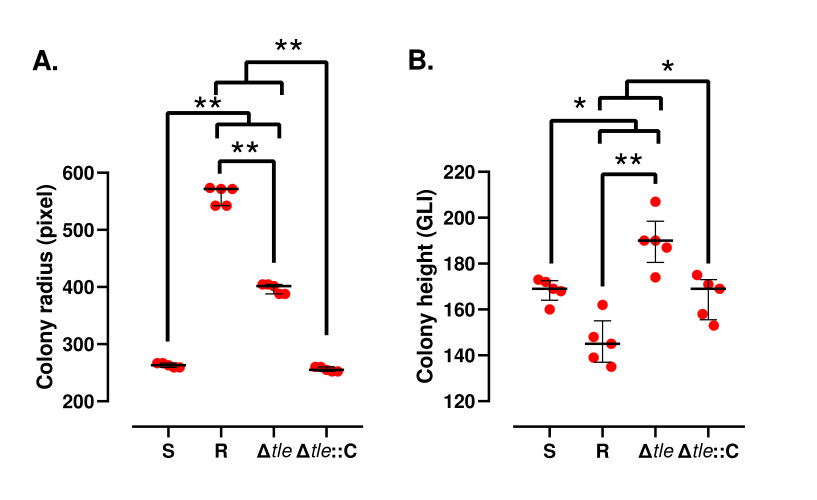
**

**Figure S5.** Colony-biofilm profile radii **(A)** and height **(B)** of each strain. *: p<0.05, and **: p<0.01.

**A B**

**Figure S6. Comparison of the GPL profile in Δ*tle* and Δ*gtf1*. (A)** TLC of the native polar extracts, developed using CHCl_3_:CH_3_OH:H_2_O (90:10:1), followed by orcinol staining and charring. The GPL profiles of Δ*tle* and Δ*gtf1* are similar with a lower R_f_ than wild-type GPL-2a and GPL-3. **(B)** MALDI-TOF positive spectrum of native polar extracts from wild-type S (blue) showing intense ions for GPL-2a at *m/z* 1257.9/1285.9 and GPL-3 at *m/z* 1404/1432 while Δ*tle* (green) and Δ*gtf1* (black) spectra produced intense ions for GPL-1b at *m/z* 1013.8/1041.8 and GPL-2b at 1159.9/1187.9 **(C)** TLC of saponified polar extracts, developed using CHCl_3_:CH_3_OH:H_2_O (90:10:1) and followed by orcinol staining and charring. The GPL profiles of Δ*tle* and Δ*gtf1* are similar with a higher R_f_ than wild-type deacetylated dGPL-2a and dGPL-3. **(D)** MALDI-TOF positive spectrum of native polar extracts from wild-type S (blue) showing intense ions for GPL-2a at 1173.4/1201.4 and GPL-3 at *m/z* 1319.3/1347.4 while Δ*tle* (green) and Δ*gtf1* (black) spectra produced ions for GPL-1b at *m/z* 1013.8/1041.8 and GPL-2b at 1159.9/1187.9 **(E)** GC analysis of itol-acetates derivatives showing 6-d-Tal in *M. abscessus* S (blue) but not in Δ*tle* (green) or in Δ*gtf1* (black). Methylated Rha is detected in all three strains.


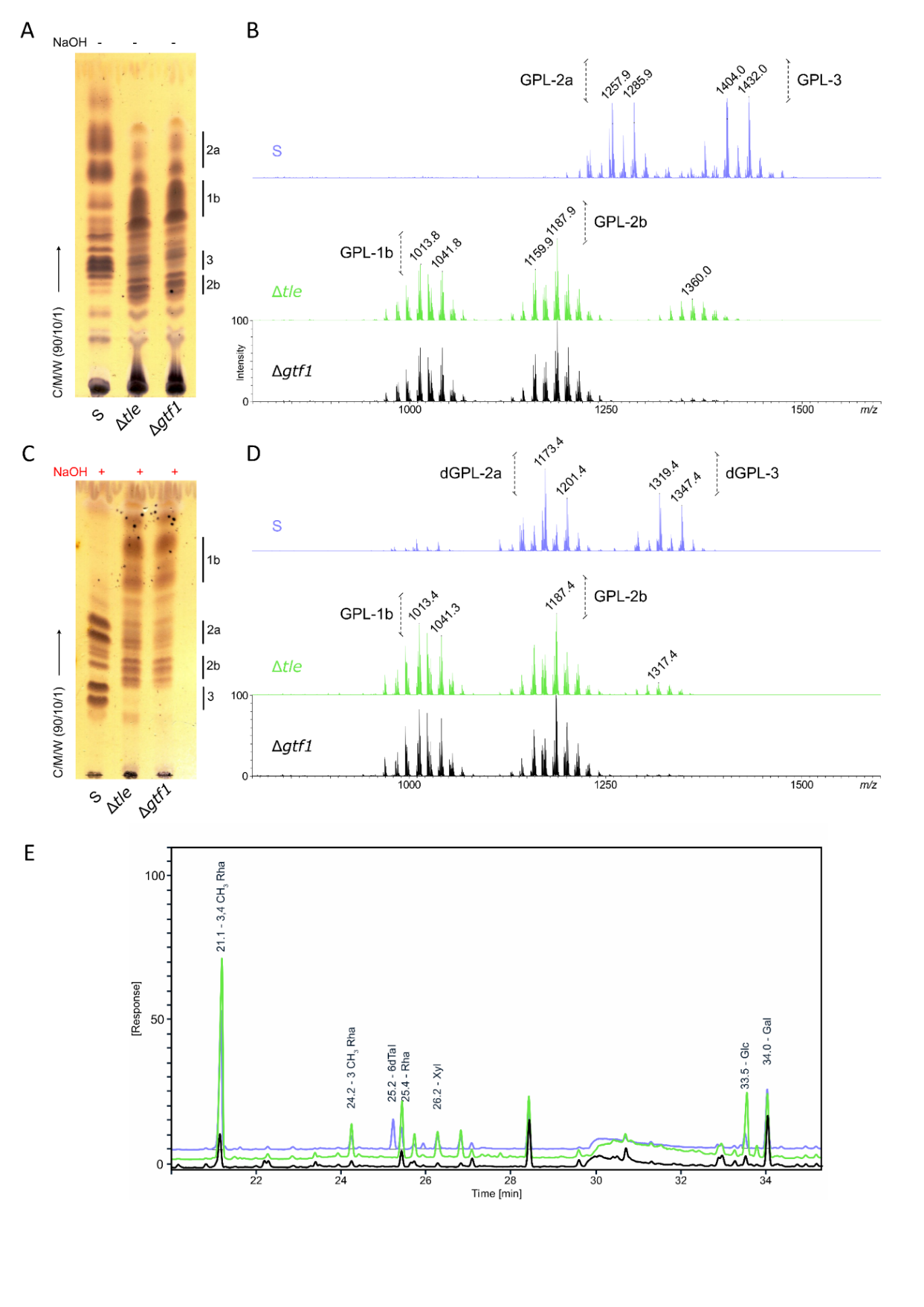


**A B**

**C D**

**E**

**
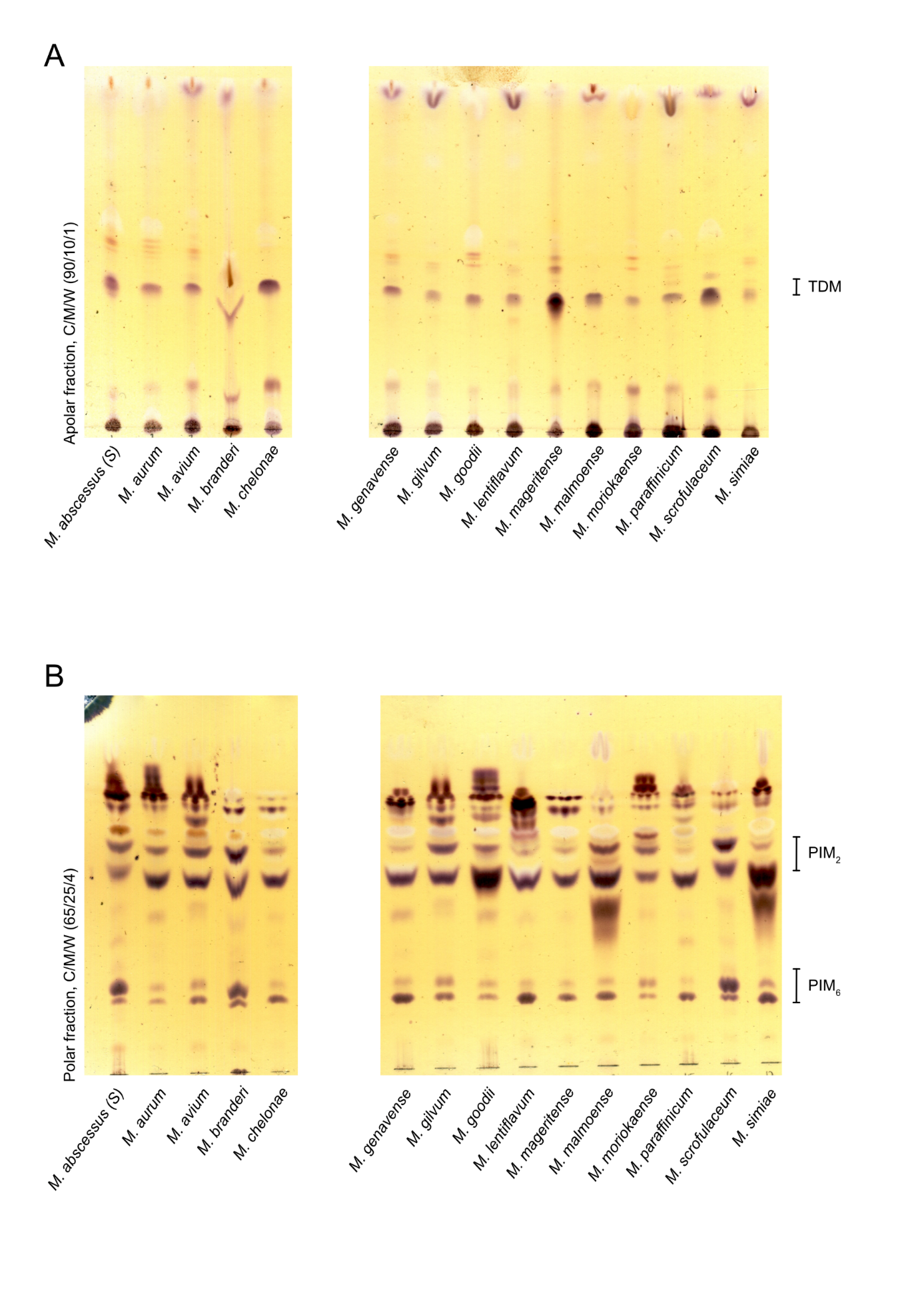
**

**Figure S7. Glycolipid profile in NTM species harbouring a *tle* orthologue.** After sequential extraction, **(A)** apolar lipids were separated using chloroform/methanol/water (90:10:1, v/v/v) followed by spraying with orcinol and charring. Putative R_f_ of trehalose dimycolate (TDM) is indicated in the right margin; **(B)** polar lipids were separated using chloroform/methanol/water (65:25:4, v/v/v) followed by spraying with orcinol and charring. Putative phosphatidylinositol dimannosides (PIM_2_) and hexamannosides (PIM_6_) are indicated in the right margin.

**
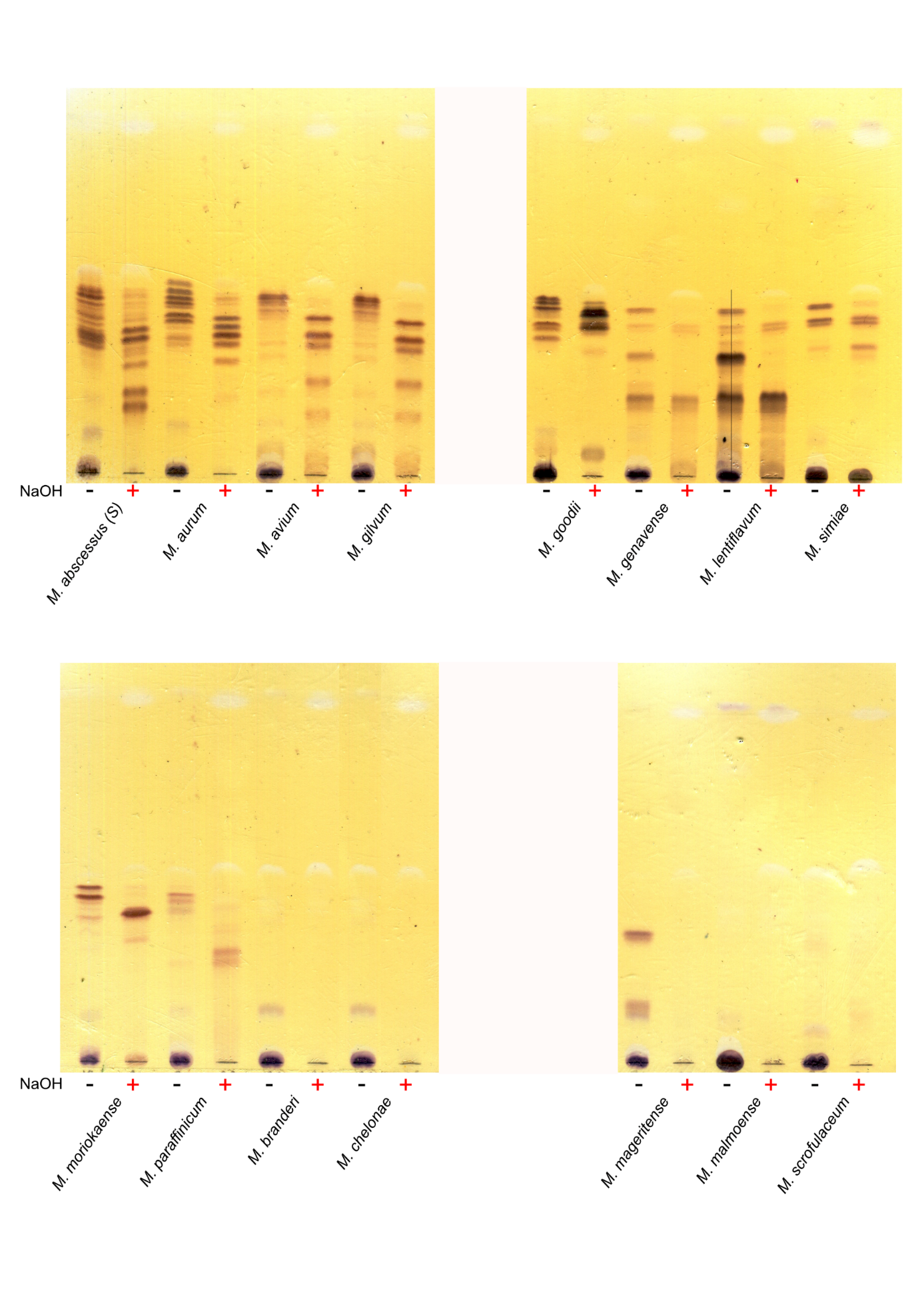
**

**Figure S8. Comparison of native and saponified polar lipid fractions from NTM harboring a *tle* ortholog.** After the extraction, the polar lipids were subjected (+) or not (-) to mild alkaline hydrolysis, and separated using chloroform/methanol/water (90:10:1, v/v/v) followed by spraying with orcinol and charring. As an example, *M. mageritense* produces a lipid with similar R_f_ as compared to GPLs in the native fraction, but upon saponification, all bands disappeared.

**Table S1.** Primers used in this study. F: Forward. R: Reverse.

| **Number** | | | **5’→3’ sequence** | **Restriction site** | | | **Primer** |
| --- | --- | --- | --- | --- | --- | --- | --- |
| **Cloning in pUX1-*katG*** | | | | | | | |
| 1 | | MAB_4111c U (F) | GAGA**TTAATTAA**AATGTTGCTGAGTTCTATGGGATCCCGT | | PacI | F | |
| 2 | | MAB_4111c U (R) | GAGA**CAATTG**TCCCGCGCCACCTGAGATGAG | | MfeI | R | |
| 3 | | MAB_4111c D (F) | GAGA**GAATTC**AAAGACTGCCCGAATCGTTTTTGAGCAAGC | | EcoRI | F | |
| 4 | | MAB_4111c D (R) | GAGA**GCTAGC**ACGGCCAGGAGCTGCTGAGT | | NheI | R | |
| **Primers to verify double homologous recombination** | | | | | | | |
| 5 | MAB_4111c | | TTCTGGCGGCTGCAGGACTTGG | | - | F | |
| 6 | MAB_4111c | | GCTTCCGTTTAAGCCGAGACTTCAAAGC | | - | R | |
| **Cloning in pMV306 under the endogenous promoter** | | | | | | | |
| 7 | MAB_4111c F | | GAGA**GGTACC**GCCTGGATGGCTACCCGC | | KpnI | F | |
| 8 | MAB_4111c R | | GAGA**GAATTC**AAAACGATTCGGGCAGTCTTTGTCGATC | | EcoRI | R | |
| **Cloning in pET30 to produce Tle in *E. coli*** | | | | | | | |
| 9 | pET30_4111c F | | **GGTACC**ATGGAGAACCTGTACTTCCAGGGT | | KpnI | F | |
| 10 | pET30_4111c R | | **GAATTC**CTATCAAAAACGATTCGGGCAGTCTTTGTCG | | EcoRI | R | |
| **Site-directed mutagenesis** | | | | | | | |
| 11 | | pET30_4111c Y181A F | AACCCAGACCGACCAACATCGCGGCGGGTACAAAACTTGCGCA | | - | F | |
| 12 | | pET30_4111c Y181A R | TGCGCAAGTTTTGTACCCGCCGCGATGTTGGTCGGTCTGGGTT | | - | R | |
| 13 | | pET30_4111c S121A F | CGGACCAGTTGGTCCTCGCAGCGTCACGAGCCGTTTATGGCGA | | - | F | |
| 14 | | pET30_4111c S121A R | TCGCCATAAACGGCTCGTGACGCTGCGAGGACCAACTGGTCCG | | - | R | |
| 15 | | pET30_4111c N210A F | TGAGTGTCTTGCGGCTGCAGGCGGTGTATGGTCCCGGGCAATC | | - | F | |
| 16 | | pET30_4111c N210A R | GATTGCCCGGGACCATACACCGCCTGCAGCCGCAAGACACTCA | | - | R | |
| 17 | | pET30_4111c R249A F | ACGAAGACGGGCGGATAGTCGCGGATTTCGTATTCATCGATGA | | - | F | |
| 18 | | pET30_4111c R249A R | TCATCGATGAATACGAAATCCGCGACTATCCGCCCGTCTTCGT | | - | R | |
|  | | | | | | | |

**Table S2.** Mycobacterial strains used in this study.

| **Name** | **Description/genotype** | **Resistance** | **Reference** |
| --- | --- | --- | --- |
| Smooth (S) *M. abscessus* | *M. abscessus* *sensu stricto*, strain CIP104536^T^, S morphotype | - | Laboratoire de Référence des Mycobactéries |
| Rough (R) *M. abscessus* | *M. abscessus* *sensu stricto*, strain CIP104536^T^, R morphotype | - | Laboratoire de Référence des Mycobactéries |
| Δ*tle* | Unmarked deletion of *MAB_4111c* in *M. abscessus* | - | This study |
| Δ*tle*::C  S pMV306-mScarlet  R pMV306-mScarlet  Δ*tle* pMV306-mScarlet  Δ*tle*::C pTEC27 | Δ*MAB_4111c* carrying pMV306-*MAB_4111c-sfGFP*  Strain CIP104536^T^ S carrying the pMV306-mScarlet  Strain CIP104536^T^ R carrying the pMV306-mScarlet  Δ*tle* carrying the pMV306-mScarlet  Δ*tle*::C carrying the pTEC27 | Kan  Kan  Kan  Kan  Kan + Hyg | This study  (1)  (1)  This study  This study |

Kan, kanamycin; Hyg, hygromycin

**Table S3.** Plasmids used in this study.

| Plasmids | | | |
| --- | --- | --- | --- |
| pUX1*-katG* | A pUX1 variant including *katG* gene of *M. tuberculosis* as a marker to counter-select in the presence of isoniazid and allowing to generate unmarked chromosomal alterations. | Kan | (2) |
| pUX1-*katG*-*MAB_4111c* | pUX1-*katG* including the upstream and downstream sequences around *MAB_4111c* | Kan | This study |
| pMV306 | Integrative vector | Kan | (3) |
| pMV306-*sfGFP* | Integrative vector labeling with GFP | Kan | (4) |
| pMV306-*MAB_4111c*-*sfGFP* | pMV306 enabling the expression of MAB_4111c-GFP in mycobacteria | Kan | This study |
| pET30 | pET-30a-c(+) carries an N-terminal His-tag; contains a strong T7lac promoter, an optimized RBS, the coding sequence for the Ek protease cleavage site, and a multiple cloning site that includes restriction enzyme sites found in many other Novagen expression vectors to facilitate insert transfer. An optional C-terminal His-Tag coding sequence is included for compatibility with purification, detection, and quantification. | Kan | Sigma Aldrich |
| pET30-*MAB_4111c* | pET-30 enabling the expression of MAB_4111c-H_6_ in *E. coli* BL21 (DE3) Star | Kan | This study |
| pET30-*MAB_4111c Y181A* | pET-30 enabling the expression of MAB_4111c-H_6_ mutated in the tyrosine 181 in *E. coli* BL21 (DE3) Star | Kan | This study |
| pET30-*MAB_4111c S121A* | pET-30 enabling the expression of MAB_4111c-H_6_ mutated in the serine 121 in *E. coli* BL21 (DE3) Star | Kan | This study |
| pET30-*MAB_4111c N210A* | pET-30 enabling the expression of MAB_4111c-H_6_ mutated at asparagine 210 in *E. coli* BL21 (DE3) Star | Kan | This study |
| pET30-*MAB_4111c R249A* | pET-30 enabling the expression of MAB_4111c-H_6_ mutated at arginine 249 in *E. coli* BL21 (DE3) Star | Kan | This study |
| pET30-*MAB_4111c S121A, N210A, R249A* | pET-30 enabling the expression of MAB_4111c-H_6_ mutated at the serine 121, arginine 249, and asparagine 210 in *E. coli* BL21 in *E. coli* BL21 (DE3) Star | Kan | This study |
| pTEC27 | Replicative vector designed to express tdTomato | Hyg | (5) |
| pMV306-mScarlet | Integrative vector designed to express mScarlett | Kan | (1) |

Kan, kanamycin; Hyg, hygromycin

**Table S4.** Logistic growth parameters estimated for the strains studied here. YM: maximum population (ABS_595nm_). Y0: starting population (ABS_595nm_). k: growth rate constant (ABS_595nm_/hrs). t: time (hours)

$$Y=\frac{YM\times Y0}{(YM-Y0)e^{-k \times t +Y0}}$$

| **Strain** | **S** | | **R** | | **Δ*tle*** | | **Δ*tle*::C** |
| --- | --- | --- | --- | --- | --- | --- | --- |
| **Best-fit values** |  | |  | |  | |  |
| YM | 1.354 | | 1.352 | | 1.507 | | 1.646 |
| Y0 | 0.1683 | | 0.06246 | | 0.008162 | | 0.1334 |
| k | 0.02215 | | 0.04807 | | 0.09264 | | 0.02598 |
| t int | 45.15 | | 20.8 | | 10.79 | | 38.49 |
| **95% CI (profile likelihood)** | |  | |  | |  | |
| YM | 1.217 to 1.583 | | 1.308 to 1.400 | | 1.468 to 1.546 | | 1.479 to 1.916 |
| Y0 | 0.1375 to 0.2010 | | 0.04016 to 0.08981 | | 0.002747 to 0.01962 | | 0.09621 to 0.1755 |
| k | 0.01833 to 0.02636 | | 0.04192 to 0.05536 | | 0.07750 to 0.1119 | | 0.02130 to 0.03126 |
| t int | 37.94 to 54.56 | | 18.06 to 23.86 | | 8.940 to 12.90 | | 31.99 to 46.94 |
| **Goodness of Fit** | |  | |  | |  | |
| Degrees of Freedom | 93 | | 93 | | 93 | | 93 |
| R squared | 0.9336 | | 0.9517 | | 0.955 | | 0.9167 |
| Sum of Squares | 0.8399 | | 1.131 | | 1.549 | | 1.899 |
| Sy.x | 0.09503 | | 0.1103 | | 0.129 | | 0.1429 |
|  |  | |  | |  | |  |
| **Number of points** | |  | |  | |  | |
| # of t values | 96 | | 96 | | 96 | | 96 |
| # Y values analyzed | 96 | | 96 | | 96 | | 96 |

**Table S5.** MIC of drugs against *M. abscessus* strains. S, susceptible; R, resistant; I, intermediate.

| **Antibiotic** | **MIC (µg/mL) (phenotype^a^)** | | | | |
| --- | --- | --- | --- | --- | --- |
|  | **Range (µg/mL)** | **S** | **R** | **Δ*tle*** | **Δ*tle*::C** |
| Trimethoprim/Sulfamethoxazole | 0.25/4.75-8/152 | >8/152(R) | >8/152(R) | >8/152(R) | >8/152(R) |
| Linezolid | 1­-32 | 32 (R) | 32 (R) | 32 (R) | 32 (R) |
| Ciprofloxacin | 0.12-4 | >4 (R) | >4 (R) | >4 (R) | >4 (R) |
| Imipenem | 2­-64 | 64 (R) | 64 (R) | 32 (R) | 64 (R) |
| Moxifloxacin | 0.25-8 | >8 (R) | >8 (R) | >8 (R) | >8 (R) |
| Cefepime | 1-­32 | >32 (R) | >32 (R) | >32 (R) | >32 (R) |
| Cefoxitin | 4-128 | 64 (I) | 128 (R) | 64 (I) | 64 (I) |
| Amoxicillin/Clavulonic acid | 2/1-64/32 | >64/32 (R) | >64/32 (R) | >64/32 (R) | >64/32 (R) |
| Amikacin | 1-­64 | 16 (S) | 32 (I) | 32 (I) | >64 (R) |
| Ceftriaxone | 4­-64 | >64 (R) | >64 (R) | >64 (R) | >64 (R) |
| Doxycycline | 0.12-16 | >16 (R) | >16 (R) | >16 (R) | >16 (R) |
| Minocycline | 1-­8 | >8 (R) | >8 (R) | >8 (R) | >8 (R) |
| Tigecycline | 0.015-4 | 0.5 (S) | 1 (S) | 1 (S) | 1 (S) |
| Tobramycin | 1-­16 | 16 (R) | 16 (R) | 16 (R) | >16 (R) |
| Clarithromycin | 0.06-16 | 8 (R) | 4 (I) | 4 (I) | 4 (I) |
| Clarithromycin (at day 15) | 0.06-16 | >16 (R) | >16 (R) | >16 (R) | >16 (R) |
| Rifabutin^b^ | 0.258-256 | 64 (R) | 16 (R) | 32 (R) | 64 (R) |

^a^The phenotype was attributed according to the breakpoints for rapidly-growing mycobacteria (6).

^b^The phenotype against rifabutin was attributed according to the breakpoint from (7).

**References**

1. Pichler, V., Dalkilic, L., Shoaib, G., Shapira, T., Rankine-Wilson, L., Boudehen, Y.-M., Chao, J. D., Sexton, D., Prieto, M., Quon, B. S., Tocheva, E. I., Kremer, L., Hsiao, W., and Av-Gay, Y. (2024) The diversity of clinical *Mycobacterium abscessus* isolates in morphology, glycopeptidolipids and infection rates in a macrophage model. *J. Med. Microbiol*. 10.1099/jmm.0.001869

2. Richard, M., Gutiérrez, A. V., Viljoen, A., Rodriguez-Rincon, D., Roquet-Baneres, F., Blaise, M., Everall, I., Parkhill, J., Floto, R. A., and Kremer, L. (2019) Mutations in the MAB_2299c TetR regulator confer cross-resistance to clofazimine and bedaquiline in *Mycobacterium abscessus.* *Antimicrob. Agents Chemother.* **63**, e01316-18

3. Stover, C. K., de la Cruz, V. F., Fuerst, T. R., Burlein, J. E., Benson, L. A., Bennett, L. T., Bansal, G. P., Young, J. F., Lee, M. H., and Hatfull, G. F. (1991) New use of BCG for recombinant vaccines. *Nature*. **351**, 456–460

4. Santucci, P., Point, V., Poncin, I., Guy, A., Crauste, C., Serveau-Avesque, C., Galano, J. M., Spilling, C. D., Cavalier, J.-F., and Canaan, S. (2018) LipG a bifunctional phospholipase/thioesterase involved in mycobacterial envelope remodeling. *Biosci. Rep.* **38**, BSR20181953

5. Takaki, K., Davis, J. M., Winglee, K., and Ramakrishnan, L. (2013) Evaluation of the pathogenesis and treatment of *Mycobacterium marinum* infection in zebrafish. *Nat. Protoc*. **8**, 1114–1124

6. Hatakeyama, S., Ohama, Y., Okazaki, M., Nukui, Y., and Moriya, K. (2017) Antimicrobial susceptibility testing of rapidly growing mycobacteria isolated in Japan. *BMC Infect. Dis*. **17**, 197

7. Cheng, A., Tsai, Y.-T., Chang, S.-Y., Sun, H.-Y., Wu, U.-I., Sheng, W.-H., Chen, Y.-C., and Chang, S.-C. (2019) *In vitro* synergism of rifabutin with clarithromycin, imipenem, and tigecycline against the *Mycobacterium abscessus* complex. *Antimicrob. Agents Chemother*. **63**, e02234-18

**Amino acid sequences of *tle* orthologs used in the phylogenetic analysis**

>sp|Q2SYH7|WBIB_BURTA dTDP-L-rhamnose 4-epimerase OS=Burkholderia thailandensis (strain ATCC 700388 / DSM 13276 / CIP 106301 / E264) OX=271848 GN=wbiB PE=1 SV=1

MSDVNASLVDGKKILVTGGAGFIGCAISERLAARASRYVVMDNLHPQIHANAVRPVALHE

KAELVVADVTDAGAWDALLSDFQPEIIIHLAAETGTGQSLTEASRHALVNVVGTTRLTDA

IVKHGIAVEHILLTSSRAVYGEGAWQKADGTIVYPGQRGRAQLEAAQWDFPGMTMLPSRA

DRTEPRPTSVYGATKLAQEHVLRAWSLATKTPLSILRLQNVYGPGQSLTNSYTGIVALFS

RLAREKKVIPLYEDGNVTRDFVSIDDVADAIVATLAREPEALSLFDIGSGQATSILDMAR

IIAAHYGAPEPQVNGAFRDGDVRHAACDLSESLANLGWKPQWSLERGIGELQTWIAQELD

RKN

>WP_005085923.1 NAD-dependent epimerase/dehydratase family protein [Mycobacteroides abscessus]

MLISGGAGFIGSALSNRLIQAGYDVAVMDVLHPQVHARGRAIDLPASVRLFTGDVTHAPD

WDAVLRLFEPSQVVHLAAETGTAQSLSEATRHGSVNVVGTTQLLDALSRSALVPDQLVLA

SSRAVYGEGAWQSGAEVFYPRPRSHAQLLAGEWDPQGPAGASATPLSSCADRTEPRPTNI

YAGTKLAQEHLLASWAAAHDTNLSVLRLQNVYGPGQSLTNSYTGIVTLFARLAREKQALE

VYEDGRIVRDFVFIDDVVDALFAAVHTPATERRTLDIGSGTATTIHELARKVADICAAPE

PVVVGKFRDGDVRAASCDIAPAVEGLGWSPKWTLEDGLHALLEWIDKDCPNRF

>WP_070933355.1 NAD-dependent epimerase/dehydratase family protein [Mycobacteroides chelonae]

MLISGGAGFIGSALSNRLVQAGYDVAVMDVLHPQVHARGRDIDLPPSVRLFTGDVTHAPDWDAVLRLFEPSQVVHLAAETGTAQSLSEATRHGSVNVVGTTQLLDALSRAAFVPDQLVLASSRAVYGEGAWQSGAEVFYPRPRSHAQLLAGQWDPKGPTGEPSEPLASCASRTEPRPTNIYAGTKLAQEHLLASWAAAHDTNLSVLRLQNVYGPGQSLTNSYTGIVTLFARLAREKQSLEVYEDGRIVRDFVFIDDVVDALFAAVHTPAVERRTLDIGSGTATTIHELARKVADICAAPEPTVVGKFRDGDVRAASCDVEPAIEQLAWSPKWTLEDGLHALLEWIDKDCPNRF

>WP_096504157.1 NAD-dependent epimerase/dehydratase family protein [[Mycobacterium] stephanolepidis]

MLISGGAGFIGSALSNRLVQAGYDVAVMDVLHPQVHARGRDIDLPPSVRLFTGDVTHAPDWDAVLRLFEPSQVVHLAAETGTAQSLSEATRHGSVNVVGTTQLLDALSRAAFVPDQLVLASSRAVYGEGAWQSGTEVFYPRPRSHAQLLAGQWDPKGPNGESSEPLASCASRTEPRPTNIYAGTKLAQEHLLASWAAAHDTNLSVLRLQNVYGPGQSLTNSYTGIVTLFARLAREKQSLEVYEDGRIVRDFVFIDDVVDALFAAVHTPAVERRTLDIGSGTATTIHELARKVADICAAPEPTVVGKFRDGDVRAASCDVEPAIEQLAWSPKWALEDGLHALLEWIDKDCPNRF

>TDZ92829.1 dTDP-L-rhamnose 4-epimerase [Mycobacteroides salmoniphilum]

MRVTELLCKEREASALSKSVLISGGAGFIGSALSNRLVQAGYDVAVMDVLHPQVHARGRAIDLPPSVRLFTGDVTHAPDWDAVLRLFEPSQVVHLAAETGTAQSLSEATRHGSVNVVGTTQLLDALSRAGLVPDQLVLASSRAVYGEGAWQSSAEVFYPRPRSHAQLLAGEWDPKGPAGERSEPLPSCAGRTEPRPTNVYAGTKLAQEHLLASWAAAHDTNLSVLRLQNVYGPGQSLTNSYTGIVTLFARLAREKQSLEVYEDGRIVRDFVFIDDVVDALFAAVHVPAVERRTLDIGSGTATTIHELARKVADICAAPEPTVVGKFRDGDVRAASCDIEPAIEQLAWSPKWTLEDGLLALLEWIDKDCPNRF

>WP_070909144.1 NAD-dependent epimerase/dehydratase family protein [Mycobacteroides saopaulense]

MLISGGAGFIGSTLSSRLVQAGYDVAVMDVLHPQVHARGRAIDLPSSVRLFTGDVTHAPDWDAVLRLFEPSQIVHLAAETGTAQSLSEATRHGSVNVVGTTQLLDALSRSGLVPDQLVLASSRAVYGEGAWQSGAQVFYPRPRSHAQLLAGEWDPKGPAGESATSLPSCAGRTEPRPTNVYAGTKLAQEHLLAAWAAAHDTNLSVLRLQNVYGPGQSLTNSYTGIVTLFARLAREKQSLEVYEDGRIVRDFVFIDDVVDALFAAVHTPAVERRTLDIGSGTATTIHDLARKVADICAAPEPVVVGKFRDGDVRAASCDIDPATERLAWSPKWSLEDGLQALLEWIDKDCPNRF

>WP_078282313.1 NAD-dependent epimerase/dehydratase family protein [Mycobacteroides franklinii]

MSRASASKSVLISGGAGFIGSTLSNRLVQAGYDVAVMDVLHPQVHPRGQAVDLPSSVRLFTGDVTHAPDWDAVLRLFEPSQIVHLAAETGTAQSLSEATRHGSVNVVGTTQLLDALSRTGLVPDQLVLASSRAVYGEGAWQAGAEVFYPRPRSHAQLVAGEWDPTGPHGESANPLASSADRTEPRPTNVYAGTKLAQEHLLAAWAAAHDTNLSVLRLQNVYGPGQSLTNSYTGIVTLFARLAREKQSLEVYEDGRIVRDFVFIDDVVDALFAAVHTPAVERRTLDIGSGTATTIHELARKVADICAAPEPVVVGKFRDGDVRAASCDIDPAIEGLAWSPKWALEDGLQALLEWIDKDCPNRF

>GBG38472.1 putative epimerase/dehydratase [Mycobacterium montefiorense]

MHVFVLPTLQTNGRRRPLTQSVLITGGAGFIGSALSRRLIKAGYDVAVLDVLHPQVHATGQAIDLPASVRLFTGDVTHAPDWDAVLRLFRPSQIVHLAAETGTAQSLSEATRHGSVNVVGTTQLLDALSRSGLVPDQLVLASSRAIYGEGAWQSGAQTFYPRPRSHAQLAAGIWDPQGPTGERAVPLASCADRTEPRPTNVYAATKLAQEHTLAAWTAAHDTNVSVLRLQNVYGPGQSLTNSYTGIVALFARLAREQHALEVYEDGRIVRDFVYIDDVVDALFASVQQPAAEQRCLDIGSGTPTTIHELAQTISAVCGAPEPVVVSKFRDGDVRAASCDIEPAKEALDWRPKWTLDDGLRELLEWIGQQYESPSTATDHTLESNRPVAEHAGRN

>WP_096286899.1 GDP-mannose 4,6-dehydratase [Mycobacterium ahvazicum]

MLITGGAGFIGSALSRRLVKAGYEVAVMDVLHPQVHAPGQAIDLPASVRLFTGDVTHAPDWDAVLRLFRPSQIVHLAAETGTAQSLSEATRHGSVNVVGTTQLLDGLSRAGFVPDQLVLASSRAVYGEGAWQSSGHIFYPRPRSHAQLVAGNWDPQGPTGEPAVPLPSCAGRTEPRPTNVYAATKLAQEHTLAAWAAAHDTNVSVLRLQNVYGPGQSLTNSYTGIVALFARLARAQHSLEVYEDGRIVRDFVFIDDVIDALFAAVQQPAPEQRCLDIGSGTPTTIHELAQTISAVCGAPEPVVVSKFRDGDVRAASCDVQPAKEALDWRPKWTLDDGLQKLLEWIGKQRESGSATTDHTLDSNRSVAEHAGRN

>WP_090601611.1 NAD-dependent epimerase/dehydratase family protein [Mycobacterium lentiflavum]

MLITGGAGFIGSALSRRLVKAGYDVAVMDVLHPQVHAPGQAIDLPAAVRLFTGDVTHAPDWDAVLRLFRPSQIVHLAAETGTAQSLSEATRHGSVNVVGTTQLLDGLSRSGFVPDQLVLASSRAVYGEGAWQTGGHIFYPRPRSHAQLVAGIWDPQSPTGEAAVPLPSCAGRTEPRPTNVYAATKLAQEHTLAAWAAAHDTNVSVLRLQNVYGPGQSLTNSYTGIVALFARLARERHSLEVYEDGRIVRDFVYIDDVIDALFAAVQQPAAEPRCLDVGSGTPTTIHELAQTISAVCGAPEPVIVSKFRDGDVRAASCDVGPAKEGLDWRPKWSLDDGLQKLLEWIGQQRESSSAATDHTVDAHRSVAEHAGRN

>WP_016706194.1 NAD-dependent epimerase/dehydratase family protein [Mycobacterium avium]

MLITGGAGFIGSALSRRLVEAGYDVALMDVLHPQVHGGDRPVELAPSVRLFTGDVTHAPDFDAVLRLFRPTQIVHLAAETGTAQSLSEATRHGSVNVVGTTQLLDALSRSGLVPEQLVLASSRAVYGEGAWQSGAEVFYPQPRSHAQLVAGRWDPQGPAGEQAVPLPSRADRTEPRPTNVYAATKLAQEHLLAAWTAAHDTNLSVLRLQNVYGPGQSLTNSYTGIVALFARLARQGQALEVYEDGRIVRDFVYIDDVVDALFAAVQRPASPQRRLDVGSGRATTIHELANTIAAMCAAPEPVVVGKFRDGDVRAASCDIEPATSQLGWHPKWTLEDGLRALLEWIGNRP

>WP_085221840.1 NAD-dependent epimerase/dehydratase family protein [Mycobacterium florentinum]

MLITGGAGFIGSALSRRLVKAGYDVAVLDVLHPQVHAPGQAIDLPASVRLFTGDVTHAPDWDAVLRLFRPSQIVHLAAETGTAQSLSEATRHGSVNVVGTTQLLDALSRSGLVPDQLVLASSRAVYGEGAWQCGTHIFYPRPRSHAQLVAGNWDPQSPTGEPAVPLPSRAGRTEPRPTNVYAATKLAQEHTLAAWTAAHDTNVSVLRLQNVYGPGQSLTNSYTGIVALFARLAREQHSLEVYEDGRIVRDFVYIDDVIDALFASVQQPAAEQRCVDIGSGTPTTIHELAKTISAVCGAPEPVVVSKFRDGDVRAASCDIEPAKEALDWRPRWTLDDGLRKLLEWIEQQRESSSATTDHAPESGRPVAEHAGRN

>WP_067173046.1 NAD-dependent epimerase/dehydratase family protein [Mycobacterium marseillense]

MLITGGAGFIGSALARRLIEAGYDVAVMDVLHPQVHGGGRPIELPPSVRLFTGDVTHAPDFDAVLRLFRPSQVVHLAAETGTAQSLSEATRHGSVNVVGTTQLLDALSRSGLVPEQLVLASSRAVYGEGSWQSGSQIFYPRPRSHAQLVAGRWDPQGPAGEQAVPLPSRADRTEPRPTNVYAATKLAQEHLLGAWTSAHDTNLSVLRLQNVYGPGQSLTNSYTGIVALFARLARQGLALEVYEDGRIVRDFVYIDAVVDALFAAVERPATQPRCLDIGSGHATTIHELANKIADMCDAPEPTVVPKFRDGDVRAASCDIEPAINALGWHPKWTLDDGLRALLDWIGERPEAAAGSDESGRPAVEQAGRR

>WP_163794538.1 NAD-dependent epimerase/dehydratase family protein [Mycobacterium stomatepiae]

MLITGGAGFIGSALSRRLVKAGYDVAVMDVLHPQVHAAGQAIDLPSSVRLFTGDVTHAPDWDAVLRLFRPSQIVHLAAETGTAQSLSEATRHGSVNVVGTTQLLDALSRAGIVPDQLVLASSRAVYGEGAWECGGQVFYPRPRSHAQLVAGNWDPQGPTGEPAVPLPSRAGRTEPRPTNVYAATKLAQEHTLAAWTAAHDTNVSVLRLQNVYGPGQSLTNSYTGIVALFARLAREQHSLEVYEDGRIVRDFVYIDEVVDALFASVQQPAIEQRCLDIGSGTPTTIHQLAQTISAVCRAPEPVVVSKFRDGDVRAASCDIEPAQEVLDWRPKWTLDDGLRKLLEWIERQRESASAATDHGRESNRSIAEHAGRN

>WP_068272774.1 NAD-dependent epimerase/dehydratase family protein [Mycobacterium mantenii]

MLISGGAGFIGSALSHRLVEAGYDVAVMDVLHPQVHGGNRPIELPPAVRLFTGDVTHAPDWDAVLRLFRPSQVVHLAAETGTAQSLSEATRHGSVNVVGTTQLLDALSRSGIVPEQLVLASSRAVYGEGAWQSGAQVFYPKPRSHAQLVAGVWDPQGPAGGQAVPLPSRADRTEPRPTNVYAATKLAQEHLLVAWTAAHDTNLSVLRLQNVYGPGQSLTNSYTGIVALFARLARQGLALEVYEDGRIVRDFVYIDAVVDALFAAIQRPATQPRSLDIGSGNATTIHELANKIAAMCDAPEPVVVPKFRDGDVRAASCDIEPAQTELGWQPKSTLDEGLDALLEWIDGQAEASDPMNESGRPVVEHAGRR

>WP_064950145.1 NAD-dependent epimerase/dehydratase family protein [Mycobacterium colombiense]

MLISGGAGFIGSALSHRLVEAGYDVAVMDVLHPQVHGGGRPIELPPTVRLFTGDVTHAPDWDAVLRLFRPSQVVHLAAETGTAQSLSEATRHGSVNVVGTTQLLDALSRSGIVPEQLVLASSRAVYGEGAWQSGERIFYPKPRSHAQLVAGNWDPQERAGEQAVPLPSRADRTEPRPTNVYAATKLAQEHLLAAWTAAHDTNLSVLRLQNVYGPGQSLTNSYTGIVALFARLARRGLALEVYEDGRIVRDFVYIDDVVDALFAAVQRPATQPRCLDIGSGKATTIHELANNIAAMCSAPEPVVTPKFRDGDVRAASCDVEPATAQLGWHPKWTLEDGLRALLEWIGNRTEASSASSEE

>WP_085289384.1 NAD-dependent epimerase/dehydratase family protein [Mycolicibacterium vulneris]

MLISGGAGFIGSALSHRLVEAGYDVAVMDVLHPQVHGGGRPIELPPTVRLFTGDVTHAPDWDAVLRLFRPSQIIHLAAETGTAQSLSEATRHGSVNVVGTTQLLDALSRSGIVPEQLVLASSRAVYGEGAWQSGAHIFYPKPRSHAQLVAGIWDPQGPAGEQAVPLPSRADRTEPRPTNVYAATKLAQEHLLAAWTAAHDTNLSVLRLQNVYGPGQSLTNSYTGIVALFARLARQGLALEVYEDGRIVRDFVYIDAVVDALFAAVQRPATRPRCLDIGSGEATTIHELANKVAAMCGAPEPVVVAKFRDGDVRAASCDIEPAQTELGWQPKSTLDEGLGALLEWIGRQPDATGISNESGRPVVENVGRR

>WP_025735807.1 NAD-dependent epimerase/dehydratase family protein [Mycobacterium genavense]

MLITGGAGFIGSALARRLVRAGYDVAVMDVLHPQVHSVGQLIDLPASVRLFTGDVTHAPDWDAVLWLFRPSQIVHLAAETGTAQSLSDATRHGSVNVVGTTQLLDALSRSGLVPEQLVLASSRAVYGEGAWRSGAQVFYPRPRSHAQLVAGIWDPQSPTGEPAVPLPSRADRTEPRPTNVYAATKLAQEHTLAAWTAAHDTNVSVLRLQNVYGPGQSLTNSYTGIVALFARLARELHSLEVYEDGQIMRDFVYIDDVVDALFASVQQPAVEHRCLDIGSGTPTTIHELAQTISAVCGAPEPVVVSKFRDGDVRAASCDIE

PATEALAWRPKWTLADGLQKLLEWIAQQRESSSAATDHAREPNHSIAEHAGRQ

>WP_221046628.1 NAD-dependent epimerase/dehydratase family protein [Mycobacterium senriense]

MLISGGAGFIGSALSHRLVDAGYDVAVMDVLHPQVHGGGRPIELPSTVRLFTGDVTHAPDWDAVLRLFRPSQVVHLAAETGTAQSLSEATRHGSVNVVGTTQLLDALSRSGIVPEQLVLASSRAVYGEGAWQSGERIFHPKPRSHAQLLAGVWDPQGPAGEQAQPLPSRADRTEPRPTNVYAATKLAQEHLLAAWTAAHDTNLSVLRLQNVYGPGQSLTNSYTGIVALFARLARQGLALEVYEDGRIVRDFVYIDAVVDALLAAVQQPATQPRCLDIGSGNATTIHELANKIAAMCGAPEPVVVPKFRDGDVRAASCDIE

PAQTELGWQPKATLDEGLGALLDWIGGQPES

>WP_202349933.1 NAD-dependent epimerase/dehydratase family protein [Mycobacterium paraintracellulare]

MLITGGAGFIGSALARRLIEAGYDVAVMDVLHPQVHGGDRPIELPPSVRLFTGDVTHAPDFDAVLRLFRPSQVVHLAAETGTAQSLSEATRHGSVNVVGTTQLLDALSRSGLVPDQLVLASSRAVYGEGSWQSGPHIFYPPPRSHAQLVAGRWDPQGPAGEQAVPLPSRADRTEPRPTNVYAATKLAQEHLLAAWTSAHDTNLSVLRLQNVYGPGQSLTNSYTGIVALFARLARQGLALEVYEDGRIVRDFVYIDAVIDALFAAVERPATQPRCLDIGSGQATTIHELANKIAALCGAPQPTVVPKFRDGDVRAASCDIEPAINELGWHPKWTLDDGLRALLDWIGERPEAAAGSNQSGRPAVEQAGRL

>WP_090423240.1 NAD-dependent epimerase/dehydratase family protein [Mycobacterium europaeum]

MLITGGAGFIGSALSHRLVLDGYDVAVMDVLHPQVHGAGRPIDLPPTVRLFTGDVTHAPDWDAVLRLCRPSQIVHLAAETGTAQSLSEATRHGSVNVVGTTQLLDALSRSGIVPDQLVLASSRAVYGEGAWQAGAHVFYPRPRSHAQLVAGQWDPVGPDGAPAVPLPSCASGTEPRPTNVYAATKLAQEHLLAAWTAAHDTNVSVLRLQNVYGPGQSLTNSYTGIVALFARLAREGQSLEVYEDGRIVRDFVYIDDVVDALYAAVRRPANESRRVDIGSGNGTTIHELANKVAAMCDAPNPTVVAKFRDGDVRAASCDIEQAETELDWHPKWTLDDGLRALLEWIAKRSESSG

>WP_067268773.1 NAD-dependent epimerase/dehydratase family protein [Mycobacterium scrofulaceum]

MLITGGAGFIGSALSHRLVQDGYDVAVMDVLHPQVHGLDRAIDLPSSVRLFPGDVTHAPDWDAVLRLCRPSQIVHLAAETGTAQSLSEATRHGAVNVVGTTQLLDALSRSGLVPDQLVLASSRAVYGEGAWRAGTDVFYPRPRSHAQLVAGQWDPLGPDGQPAVPLPSCASRTEPRPTNVYAATKLAQEHLLAAWTTAHDTNVSVLRLQNVYGPGQSLTNSYTGIVALFARLAREGQSLEVYEDGRIVRDFVYIDDVVDALYAALQRPATESRRLDIGSGNGTTIHELADRVAALYGAPHPTVVSKFRDGDVRAASCDIGPAEAELDWRPKWTLDEGLRALLEWIDKQAESSS

>WP_085180216.1 GDP-mannose 4,6-dehydratase [Mycobacterium bohemicum]

MLITGGAGFIGSALARRLVEAGYDVAVMDVLHPQVHGAGSAIDLPPSVRLCTGDVTHAPDWDAVLRLCRPSQIVHLAAETGTAQSLSEATRHGSVNVVGTTQLLDALSRSTHIPEQFVLASSRAVYGEGSWQYGSEVFYPQPRSHAQLVAGSWDPQGPAGEAAVPLPSCATRTEPRPTNIYAATKLAQEHILAAWAAAHDTRLSVLRLQNVYGPGQSLTNSYTGIVALFARLARGQRALDVYEDGRIVRDFVYIDDVVEALFAAIEAPAAPRRCLDIGSGVPTTIHELARTIAAICDAPEPVVVGKFRDGDVRAARCDIEPATKELDWRPKWALEEGLRALLDWIGEQPDEAASGPTDHTRVTASVK

>WP_163754862.1 NAD-dependent epimerase/dehydratase family protein [Mycobacterium botniense]

MLVTGGAGFIGSALARRLVEAGYDVAVMDVLHPQVHGARPMIDLPPSVRLFTCDVTHGPDWDAALRLFQPSQIVHLAAETGTAQSLTLATRHGSVNVVGTAQLLDALTRSALVPDQFILASSRAVYGEGAWRCGTHVFYPRPRSHAQLLAGIWDPQGPAGEPAVPLPSCAGQTEPRPTNIYAATKLAQEHMLAAWATAHDANLSVLRLQNVYGPGQSLTNSYTGIVALFARLAREQRALEVYEDGRIVRDFVYIDDVVDAMTAAVQNPAAESRCLDIGSGIATTIHELARKIAALCNAPEPVVVPKFRDGDVRAASCTIEPATNELHWRPKWGLEDGLHALLEWIGKQPE

>WP_067316854.1 NAD-dependent epimerase/dehydratase family protein [Mycobacterium alsense]

MLITGGAGFIGSALARRLVKAGYDVAVMDVLHPQVHGEGAAIDLPPSVRLCTGDVTHAPDWDAVLRLCRPSQVVHLAAETGTAQSLSEATRHGSVNVVGTTQLLDALSRSAHVPDQLVVASSRAVYGEGAWRCGSQVFYPRPRSHAQLLAGLWDPQGPTDDPAVPLPSCAGRTEPRPTNIYAATKLAQEHILGAWTAAHDSRLSVLRLQNVYGPGQSLTNSYTGIVALFARLAREQRSLEVYEDGHIVRDFVYIDDVVDALFAALEKPAAQPRCLDIGSGVPTTIHELAQRIADICGAPEPVVVAKFRDGDVRAASCDIGPAKNALDWRPKWALGDGLHALLDWIGTQPESPVKPSDHMPMTALLP

>WP_163716191.1 NAD-dependent epimerase/dehydratase family protein [Mycobacterium timonense]

MLITGGAGFIGSALARRLIEAGYDVAVMDVLHPQVHGGDRPIELPPSVRLFTGDVTHAPDFDAVLRLFRPSQVVHLAAETGTAQSLSEATRHGSVNVVGTTQLLDALSRSGHVPDQLVLASSRAVYGEGSWQSGSHIFYPPPRSHAQLVAGRWDPQGPAGEQAVPLPSRADRTEPRPTNVYAATKLAQEHLLAAWTSAHDTNLSVLRLQNVYGPGQSLTNSYTGIVALFARLARQGLALEVYEDGRIVRDFVYIDAVVDALFAAVQRPATQPRCLDIGSGQATTIHELANKIAAMCGAPEPTVVPKFRDGDVRAASCDIEPAINELGWRPERSLDDGLRALLDWIAERPEAAAGSNESGRPVVEQAGRR

>WP_065483718.1 NAD-dependent epimerase/dehydratase family protein [Mycobacterium malmoense]

MLITGGAGFIGSALSHRLVEAGYDVAMMDVLHPQVHGADRPLDLPSSVRLLTGDVTHAPDWDAVLRLCRPSQIVHLAAETGTAQSLSEATRHGSVNVVGTTQLLDALSRGGLVPDQLVLASSRAVYGEGAWQAGGHIFYPRPRSHAQLLARQWDPQGPDGQPAVPLPSSASTTESRPTNVYAATKLAQEHLLAAWTAAHDTNLSVLRLQNVYGPGQSLTNSYTGIVALFARLAREGQTLEVYEDGRIVRDFVYIDDVVEALFAALERPATESRRLDIGSGKATTIHELANTVADMCGAPEPTVVTKFRDGDVRAASCDIAPAGAELDWRPRWTLGEGLPALLEWISKES

>WP_218894647.1 SDR family NAD(P)-dependent oxidoreductase [Mycolicibacterium vinylchloridicum]

MPNISKTVLITGGAGFIGSTLARRLVEAGYDVAVMDILHPQVHAAGAPIDLPRSVRLFTGDVTHAPDCDAVLRLFKPSQIVHLAAETGTAQSLSEASRHGSVNVVGTTQLLDALSRCGHVPDQLVLASSRAVYGEGAWQSGTDIFYPPPRSHAQLAAGIWDPQGPTGDSAVPLASSAERTEPRPTNIYAATKLAQEHILASWTSAHDTKLSVLRLQNVYGPGQSLTNSYTGIVALFARLSREKQALEVYEDGRIVRDFVYIDDVVEGLFAAIESPATPSRYVDIGSGVPTTIGELAQQLAAICGAPEPVVVGKFRDGDVRAARCDIEPATIQLGWRPKWSLEEGMRALLDWIGK

>WP_048893747.1 NAD-dependent epimerase/dehydratase family protein [Mycobacterium heckeshornense]

MLVTGGAGFIGSALSRRLVKAGYDVAVMDVLHPQVHGGRQAIDLPPSVRFFTGDVTHAPDWDAVLRLFQPSQIVHLAAETGTAQSLTEATRHGLVNVVGTTQLLDALSRSALVPHQIVLASSRAVYGEGAWQAGDEVFYPRPRSHARLLAGIWDPVGPTGEPALPLPSCAGHTEPRPTNIYAATKLAQEHLLAAWTAAHDANLSVLRLQNVYGPGQSLTNPYTGIVALFARLARAQRPPEVYEDGRIVRDFVFIDDVVDAVFAALERPATESRCLDIGSGTATTIHQLARTIAAMCDAPEPTVVAKFRDGDVRAASCSIEAAMNELHWRPKWGLEDGLHALLEWISEEPELPLEPSDHTQAPGRPE

>WP_163807615.1 NAD-dependent epimerase/dehydratase family protein [Mycolicibacterium anyangense]

MLITGGAGFIGSALSRRLVDAGYDVAVLDILHPQVHGEHAAITLPASVRLFTGDVTHAADVDAVLRLFRPAQIVHLAAETGTAQSLSEATRHGSVNVVGTTQLVDALSRAGHVPDQLVLASSRAVYGEGAWQSGSEVFYPAPRSHAQLQAGTWDPQGPTGQPAIPLASSAGRTEPRPTNIYAATKLAQEHILAAWAAAHDTSLSVLRLQNVYGPGQSLTNSYTGIVALFARLSRQQQALEVYEDGRILRDFVYIDDVVDALFAAIATPAKPVRHLDVGSGVPTTIHELARALAGICGAPEPVVVGKFRDGDVRAARCDVEQTVRELDWQPKWTLEDGMRGLLDWIGQQSPRSPE

>WP_083173531.1 NAD-dependent epimerase/dehydratase family protein [Mycobacterium paraseoulense]

MLITGGAGFIGSALAHRLVEDGYDVAVMDVLHPQVHRRDRPIDLPSSVRLFTGDVTHAPDWDAVLRLYRPSQIVHLAAETGTAQSLSEATRHGSVNVVGTTQLLDALSRSGIVPDQLVLASSRAVYGEGAWQAGTYVFYPRPRSHAQLVAGQWDPLGPEGEPAVPLPSCASATEARPTNVYAATKLAQEHLLTAWTAAHDTKVSVLRLQNVYGPGQSLTNSYTGIVALFARLACHGQSLEVYEDGRIVRDFVYIDDVVDALCAALRRPATESRRLDIGSGSGTTIHELANKVAALCDAPDPTVVPKFRDGDVRAASCDIAQAETELDWRPKWTLDDGLLALLEWIGKQSESSG

>WP_094484155.1 NAD-dependent epimerase/dehydratase family protein [Mycolicibacterium sphagni]

MLITGGAGFIGSALARRLVEADYDVAVMDILHPQVHAEGATIDLPSSVRLFTGDVTHAADCDAALRLFKPSQIVHLAAETGTAQSLSEATRHGSVNVVGTTQVLDALSRCGHVPDQLVLASSRAVYGEGAWQSGDDVFYPLPRSHAQLVAGQWDPQGPNGAAAEPLASSAVRTEPRPTNIYAATKLAQEHILAAWTSAHDTKLSVLRLQNVYGPGQSLTNSYTGIVALFARLAREKNALEVYEDGRIMRDFVFIDDVVEGLFAAIESPAAPSRYVDIGSGVPTTIHELAQQLAAICGAPEPVVVGKFRDGDVRAARCDIDLATNELGWRPKWSLEEGMRALLDWIATS

>WP_163777083.1 NAD-dependent epimerase/dehydratase family protein [Mycobacterium cookii]

MLITGGAGFIGSALARRLVNVGYDVAVMDLLHPQVHGDHPAIELPPSVRLFTGDVTHGPDLDAVLRLFQPLQIVHLAAETGTAQSLSEATRHGSVNVVGTTQLLDALSRAGHVPDQLVLASSRAVYGEGAWQCGSQIFYPQPRSHAQLVAGIWDPQGLTEDSAVPLASCAGRTEPRPTNIYAATKLAQEHILAAWTAAHDTNLSVLRLQNVYGPGQSLTNSYTGIVALFARLAGEQHALEVYEDGRIVRDFVYIDDVVEALFAAIERPAAQPRCVDIGSGIPTTIHELAQQIAAICGAPEPIVVGKFRDGDVRAARCDIGPATKELGWHPKWTLEDGLRALLDWIGGRSEIVSSISEKMQFSR

>WP_099038937.1 NAD-dependent epimerase/dehydratase family protein [Mycobacterium neglectum]

MLITGGAGFIGSALSRRLVEAGYDVAVMDVLHPQVHAPGRAVELPPSVRLFTGDVTHAPDCDAVLRLVQPSQIVHLAAETGTAQSLSQATRHGSVNVVGTTQLLDALGRSEIVPDQLVVASSRAVYGEGAWQCGTDIFYPRPRSHAQLAAGIWDPQGPTDKEAVPLPSCASRTEPRPTNVYAATKLAQEHILAAWTTAHDTNLSVLRLQNVYGPGQSLTNSYTGIVALFARLARERQPLQVYEDGRIVRDFVYINDVVGALFATVQQPATGLRCLDVGSGSATTIHELATRIATICDAPEPVVVPKFRDGDVRAASCLIEPAEDELGWRPRHALDEGLLALLEWIGEQQPALPSPQAQ

>WP_085235284.1 NAD-dependent epimerase/dehydratase family protein [Mycobacterium conspicuum]

MLITGGAGFIGSALARRLVAAGHDVAVVDILHPQVHAAGAAIDLPPSVRLFTGDITHAPDCDAVLRLFRPSQIVHLAAETGTAQSLSEATRHGSVNVVGTTQLLDALSRSGQVPEQFVLASSRAVYGEGSWQYGSEVFYPPPRSHAQLVAGRWDPQGPAGEAATPLPSRAGRTEPRPTNIYAATKLAQEHILAAWAASHDTRLSVLRLQNVYGPGQSLTNSYTGIVALFARLAREQRALDVYEDGQIVRDFVYIDDVVEALFAAIQAPAAGPRCLDIGSGTPTTIHDLARTIAAICGAPEPVVVAKFRDGDVRAARCDVEAARQELGWSPRWALQDGLRALLDWIGGQPESPSAPSGHPHATALAN

>SPM43095.1 Nucleoside-diphosphate-sugar epimerase, partial [Mycobacterium numidiamassiliense]

LSGSVLVTGGAGFIGSALARRLVTVGYEVAVMDVLHPQVHTGHQAIDLPPSVRLLTGDVTHAPDWDAVLRLCRPSQIVHLAAETGTAQSLSEATRHASVNVVGTTQLLDSLSRAAHVPDQLVLASSRAVYGEGAWQSGSHIFYPQPRSHAQLVAGIWDPQGPGHDAAAPLSSCAGRTEPRPINIYAATKLAQEHLLAAWATAHDTNLSVLRLQNVYGPGQSLTNSYTGIVALFARLAREQRSLEVYEDGRIVRDFVYIDDVVEALFAAIERPAVQSRCLDIGSGTPTTIHELALQVAGICAAPEPIVVGKFRDGDVRAAMCAIEPAKNQLAWCPRWALEEGLRRLLDWISEQAELRSATAEHAAPMKLGPR

>WP_062538377.1 NAD-dependent epimerase/dehydratase family protein [Mycobacterium celatum]

MLVTGGAGFIGSALSRRLVKAGYDVAVMDVLHPQVHAGRDAIDLPRSVQLFTGDVTHAPDWDAVLRLFRPAQVVHLAAETGTAQSLTEATRHASVNVVGTAQLLDALSRAGHVPDQFVLASSRAVYGEGAWQSGDEVFYPPPRSHAQLLAGIWDPQGPTGEAAVPLPSCACRTEPRPTNIYAATKLAQEHMLTAWTTAHDASLSVLRLQNVYGPGQSLTNSYTGIVALFARLAREQRPLEVYEDGRIVRDFVFIDDVVDALFSTLQRPAVESRCLDIGSGSATTIHELAREIAAMCDAPEPVVVPKFRDGDVRAASCIVEPAQNELDYRPKWALEEGLRTLLEWIGRQPELPSVPSDHAT

>WP_014208782.1 NAD-dependent epimerase/dehydratase family protein [Mycolicibacterium rhodesiae]

MLITGGAGFVGSALSRRLVKDGYDVAVMDVLHPQVHSGSQPIDLPPSVRLFTGDVTHAPDIDAVLRLFRPTQIVHLAAETGTGQSLAEATRHGSVNVVGTTQLLDALTRSGLVPEQLVVASSRAVYGEGAWQCATQAFYPRPRSHAQLSAGIWDPQGPSGESAAPLPSSADRTEPRPTNIYAATKLAQEHMLAAWTAAHDTNLSVLRLQNVFGPGQSLTNSYTGIVALFARLARDHQTLEVYEDGRIIRDFVFIDDVADALLAAVKKPATESRCVDIGSGTGTTIHELARMIAANCGAPEPKVVSKFRDGDVRAASCSID

AAQDDLLWRPNWTLDDGVKALLEWIGTQSVEVR

>WP_096439479.1 NAD-dependent epimerase/dehydratase family protein [Mycobacterium shigaense]

MLVTGGAGFIGSALARRLVTAGYDVAVLDVLHPQVHTGHQAIDLPPSVRLFTGDVTHAPDCDAVLRLFRPSQIVHLAAETGTAQSLSEATRHGSVNVVGTTQLLDALSRCAHVPDQLVLASSRAVYGEGAWQSGSQTFYPQPRSHAQLLAGVWDPAGPTGESAVPLPSCAGRTEPRPTNIYAATKLAQEHLLAAWAAAHDTELSVLRLQNVYGPGQSLTNSYTGIVTLFARLAREQRPLEIYEDGQIVRDFVYIDDVVEALFAAIDRPATQPRLLDVGSGIPTTIHELAHRIASLCDAPEPIVVGKFRDGDVRAAKCDIEPATAELEWHPAWTLEHGLRALLDWIAEQPELPSSSIDHTHTPSRPANQHARIPLG

>WP_045383997.1 NAD-dependent epimerase/dehydratase family protein [Mycobacterium kyorinense]

MLVTGGAGFIGSALSRRLIKAGYDVAVMDVLHPQVHAGHGPIDLLPSVRLFTGDVTHAPDWDAVLRLFRPSQIVHLAAETGTAQSLTEATRHASVNVVGTAQLLDGLSRAGHVPDQFVLASSRAVYGEGAWQSGEEVFYPRPRSHAQLLAGIWDPQGPTGEAAVPLPSCANRTEPRPTNIYAATKLAQEHMLTAWTTAHDASLSVLRLQNVYGPGQSLTNSYTGIVALFARLARVQSPLEVYEDGRIVRDFVFIDDVVDALLTTLQRPAAQPRCLDIGSGSATTIHELARKVAAMCDAPDPVVVPKFRDGDVRAASCSIEPAKKELDWRPKWALEGGLRALLDWIGRQDELPSVPSDPAT

>WP_066910987.1 NAD(P)-dependent oxidoreductase [Mycobacterium interjectum]

MLITGGAGFIGSALARRLVQAGHDVAVMDVLHPQVHGEHVAIDLPPSVRLCTGDVTHAPDWDAVLRLCRPSQVVHLAAETGTAQSLSEATRHGSVNVVGTTQLLDALSRSAHVPDQLVVASSRAVYGEGAWQCGAQIFYPQPRSHAQLLAGIWDPPGPTDDPAVPLPSRAGRTEPRPTNIYAATKLAQEHILSAWTAAHDTHLSVLRLQNVYGPGQSLTNSYTGIVALFARLAREQRTLEVYEDGQIVRDFVYIDDVVEALFAAIEKPAAQPRCLDIGSGVPSTIHELAQQIAAICDAPEPKVVAKFRDGDVRAARCDIEPATKALDWHPKWALEDGLRALLDWIGEQPESVKASDHTPTTGVLK

>WP_163736463.1 NAD(P)-dependent oxidoreductase [Mycobacterium gallinarum]

MLVTGGAGFIGCALTRRLVNAGHEVAIMDVLHPQVHGGRQAIDLPPSVKMFTGDVTHAPDLDAVLRLFRPAQLVHLAAETGTGQSLTHATRHGSVNVVGTAQLLDALSRSALVPDQLVLASSRAVYGEGAWRCGTRNFYPRPRSHAQLAAGVWDPKGPMDEPAVPLPSRADETEPRPTNIYAATKLAQEHMLSAWAAAHDTRLSVLRLQNVYGPGQSLTNSYTGIVALFARLARAQRTLEVYEDGRIVRDFVFIDDVADALFATVEAPATEQRCLDIGSGIATTIHELAREVALICNAPEPIVVPKFRDGDVRAASCVISPALSDLHWRPKWTLQDGLHRLLKWIDEQPETRS

>WP_163769573.1 NAD-dependent epimerase/dehydratase family protein [Mycolicibacterium parafortuitum]

MLITGGAGFIGSALARRLVDAGYDVAVMDVLHPQVHSRNSTVDLPSAVRFFTGDVTHAPDWDAVLRLIQPSQIVHLAAETGTAQSLSEATRHGSVNVVGTTQLLDALSRAEHVPEQFVLASSRAVYGEGAWESDGHIFYPPPRSHAQLLAGIWDPKGPTGKAAEPISSSAGRTEPRPTNVYASTKLAQEHILTAWAAAHDSNVSVLRLQNVYGPGQSLTNSYTGIVALFARMSRRRLPLEVYEDGRILRDFVFIDDVVDALFAAVRRPATLTRRVDIGSGVPTTIHELAAMTAALCGAPDPTVVGKFRDGDVRAARCDIAPATTELDWRPKWFLEDGLRALLNWIDGRPEIADSASLVDS

>AQT79176.1 NAD-dependent dehydratase [Mycolicibacterium litorale]

MSESVLITGGAGFIGSALSRRLAEAGYDVAVMDILHPQVHAENTTIDLPPSVRLYTGDVTHAPDCDAVLRLFKPSQIVHLAAETGTAQSLTEATRHGSVNVVGTTQLLDALSRCGHVPDQVVLASSRAVYGEGAWRAGDEVFYPHPRSHAQLVAGRWDPQGPSGEAAVPLASSAMRTEPRPTNIYAATKLAQEHILAGWTAAHDTRLSVLRLQNVYGPGQSLTNSYTGIVALFARLSRQKQALEVYEDGQILRDFVYIDDVVEALYAAVSGPAEPSRYVDIGSGVPTTIHQLAQQLATSCGAPDPVVVGKFRDGDVRAARCDIEPAVEDLNWRPAWSLEKGTQALLDWIEN

>RDH76130.1 NAD-dependent epimerase/dehydratase family protein [Mycolicibacterium moriokaense]

MSETVLISGGAGFIGSALSYRLVQAGHDVAVMDILHPQVHADRLDLDLPRSVRLFTGDVTHAPDWDAVLRLCRPSQIVHLAAETGTAQSLSEATRHGLVNVVGTTQLLDALGRWNTVPDQLVLASSRAVYGEGAWGCGDRTFYPPPRSHAQLTAGNWDPQGPMGEQAVSVPSRAGRTEPHPTSVYAGTKLAQEHLMAAWAAAHDTRLSVLRLQNVYGPGQSLTNSYTGIVTLFSRLARERRSLEVYEDGRIVRDFVYIDDVVEALFATVQDPHVSRGCLDIGSGVATTIHELACQVADICGAPEPVVVGKFRDGDVRAASCDIDPAKNALAWAPKWTLEEGLRELLDWIANGTPNRLLS

>WP_235881816.1 NAD-dependent epimerase/dehydratase family protein [Mycolicibacterium vanbaalenii]

MLITGGAGFIGSTLARRLAHAGYDVAVMDVLHPQVHGRDAVIDLPPTVRLFTGDVAHAPDCDAVLRLVQPSQIVHLAAETGTAQSLSEATRHSSVNVVGTTQLLDALSRAGHVPDQFVLASSRAVYGEGAWQSDAEIFYPQPRSHAQLQAGIWDPQGPTGDPAVPLPSAASRTEPRPTNIYAATKLAQEHIMAAWTAAHDTDLSVLRLQNVYGPGQSLTNSYTGIVALFARLSREQLPLEVYEDGRILRDFVYVDDVVEALFAAIQRPAAHPRCLDIGSGVPTTIHELARQAATLCDAPEPVVVGKFRDGDVRAARCDIGPATKELDWHPKWTLEDGLAALLDWIDGHPEFSSRGAE

>WP_085177476.1 NAD-dependent epimerase/dehydratase family protein [Mycobacterium paraense]

MSTRVLITGGAGFIGSALTRRLLDAGYDVAVMDVLHPQVHGDRAAIDLPPSARFFTGDVTHAPDCDAVLRLFRPSQIVHLAAETGTAQSLSEATRHGSVNVVGTTQLLDALSRSGHTPDQLVLASSRAVYGEGAWQSGSQIFYPQPRSHAQLLAGIWDPQGPTDDPVVPLASSAARTEPRPTNIYAATKLAQEHILAAWTAAHDTNLSMLRLQNVYGPGQSLTNSYTGIVALFARLARQGQALEVYEDGRILRDFVYIDDIVEALFAAIEKPANGSRCLDIGSGIPTTIHQLADQIAAICGAPQPNVVGKFRDGDVRAARCDIDPAKNELDWRPKWTLEDGLPALLDWIGEQA

>WP_085077790.1 NAD-dependent epimerase/dehydratase family protein [Mycobacterium palustre]

MLITGGAGFIGSALARRLVGAGYDVAVMDVLHPQVHAGHDAIDLPPSVRLFTGDVTHAPDWDAVLRLFRPTQVVHLAAETGTAQSLSEATRHGSVNVVGTTQLLDALGRSAHAPDQLVLASSRAVYGEGAWRCGSVTFYPQPRSHAQLLAGIWDPQAPTGGPAVPLASRADRTEPRPTNIYAATKLAQEQILGAWAAAHDARLSVLRLQNVYGPGQSLTNSYTGIVALFARLARERRALEVYEDGRIVRDFVYIDDVVDALFAAIEAPGSRCLDIGSGVPTTIHELARQIAAICDAPEPTVVGKFRDGDVRAARCDIEAAKLELGWRPKVTLADGLRALLAWIAERAESRVAPSAHLQATAALT

>WP_126336122.1 NAD-dependent epimerase/dehydratase family protein [Mycolicibacterium chitae]

MARSVLITGGAGFIGSALSSLLVASGYDVAVMDVLHPQVHSGSGDISLPQSVRLFTGDVTHAPDWDAVLRLFKPSQIVHLAAETGTAQSLSEATRHGSVNVVGTTQLLDALSRSGMVPEQLVLASSRAVYGEGSWRVGAQTFYPLPRSHAQLVAGSWDPQGPEGGPAIPIPSRASLTEPRPTNIYAATKLAQEHLLAAWAAAHDTNLSVLRLQNVYGPGQSLTNSYTGIVALFARLARAGETLEVYEDGRIVRDFVFIDDVVEALFSAVRSPARGQRCLDIGSGNATTIHELAREVARICSAPEPVVVPKFRDGDVRAASCELEPAISALDWRPKWSLADGLPALLEWIENS

>WP_270924280.1 NAD-dependent epimerase/dehydratase family protein [Mycobacterium xenopi]

MLVTGGAGFIGSALARRLSNAGHDVAVMDVLHPQVHAGNQPPDLPAAVRFFTGDVSHAPDWDAVLRLFQPRQIVHLAAETGTAQSLTEASRHGLVNVVGTAQLLDALTRSALVPDQLVLTSSRAVYGEGAWQSGSEVFYPRPRSHAQLLAGEWDPVGPTGIPAVPLPSCAGRTEPRPTSIYAATKLTQEHMLAAWAAAHDTPLTVLRLQNVYGPGQSLTNPYTGIVALFARSARAQLPLEVYEDGRILRDFVFIDDVVDALVAATRKPANGSRWLDIGSGIATTIHQLARKIATLCDAPEPIVVAKFRDGDVRAASCTIEPAANELDWHPKWTLDDGLRALLEWIGQT

>WP_199267786.1 SDR family NAD(P)-dependent oxidoreductase [Mycolicibacterium smegmatis]

MSNTVLITGGAGFIGSALAQRLTHEGYEVAVMDLLHPQVHARGSKVDLPRSVRLFTGDVTHAPDWDAVLRLFQPAQVVHLAAETGTAQSLSEATRHSSVNVVGTTQLLDALSRADLVPDQVVLASSRAVYGEGEWRTATDSTFYPLPRSHAQLAAGRWDPVGPSGEHAVPLPSCASRTEPRPTNVYAATKLAQEHVLTAWTAAHDTNLSVLRLQNVYGPGQSLTNSYTGIVALFARLAREQQTLEVYEDGNILRDFVFIEDVVEALYAAIRRPADQRRCLDIGSGVGSSIHALAQKVAGICGAPTPKVVGKFRDGDVRAASCDIEPARMELDWRPKWTLDDGLPALLDWIAKFDVSR

>WP_128109332.1 NAD-dependent epimerase/dehydratase family protein [Mycolicibacterium elephantis]

MLITGGAGFIGSALARRLVEAGYDVAVMDILHPQVHAEHTAIDLPSPVRLFTGDVTHAPDCDVVLRLFKPTQIVHLAAETGTAQSLSEASRHGSVNVVGTTQLLDALSRCGHVPDQLVLASSRAVYGEGAWQSGTDVFYPPPRSHAQLAAGVWDPQGPTGEPATALASSAGRTEPRPTNVYAATKLAQEHILAAWTAAHDTRLSVLRLQNVYGPGQSLTNSYTGIVALFARLGRQRQVLEVYEDGRIVRDFVYIDDVVEALFSAIEEPAAQSRCVDIGSGVPTTIHELARQLATLCDAPEPVVVAKFRDGDVRAAKCDVEQAMRELNWRPKWSLGEGTRALLDWIGE

>WP_083133625.1 NAD-dependent epimerase/dehydratase family protein [Mycobacterium branderi]

MLVTGGAGFIGSALSHRLVKDGCDVAVMDVLHPQVHAGHATIDLPPSVRLFTGDVTHAPDWDAVLRLFRPSQIVHLAAETGTAQSLTEATRHASVNVAGTAQLLDALSRAGHVPDQFVLASSRAVYGEGAWQSGKEVFYPRPRTHAQLLAGIWDPQGPTGEAAVPLPSRAGRTEPRPTNIYAATKLAQEHMLTAWTTAHDASLSVLRLQNVYGPGQSLSNSYTGIVALFARLACEQRPLEVYEDGRIVRDFVFIDDVVDALFATLQGPAAQSRCLDIGSGNATTIHELALKLAAICDAPEPVVVAKFRDGDVRAASCSIEPAKTELDWHPNWSLEGGLRALLEWIGGQDELPCVPSDHAM

>WP_036468707.1 GDP-mannose 4,6-dehydratase [Mycobacterium triplex]

MLITGGAGFIGSALSHTLVKAGYDVAVMDVLHPQVHAAGKPLDLPPSVRVFTGDVTHAPDCDAVLRLFRPTQIVHLAAETGTAQSLSEATRHGSVNVVGTTALLDALSRSGHVPDQLVLASSRAVYGEGAWQCGTQIFYPGPRSHAQLVAGQWDAQGPTGETGVPLASCAGRTEPRPSNIYAATKLAQEHILAAWAAARDTNLSVLRLQNVYGPGQSLTNPYTGVVPFFAQVSREKRASEVYEDGRIVRDFVYIEDVVDALFATVQDPATGARCLDIGSGVATTIHQLATKIAANFGAPEPVITAKFRDGDVRAASCDIEPAQRALRWRPQWSLDDGLRVLLEWIGEQLESSSAADDSTLKPDHPVAQRAGR>WP_077098892.1 NAD(P)-dependent oxidoreductase [Mycobacterium terramassiliense]

MLITGGAGFIGSALARRLVGAGYDVAVMDVLHPQVHGEHGAIDLPPSVRLCTGDVTHAPDWDAVLRLCRPSQVVHLAAETGTAQSLSEATRHGSVNVVGTTQLLDALSRAAHVPDQIVLASSRAVYGEGAWQSGSQVFYPQPRSHAQLASGVWDPHGPTDLPAIPLPSSAGRTEPRPTNIYAATKLAQEHILAAWTAAHDSRLSVLRLQNVYGPGQSLTNSYTGIVALFARLARQQRALEVYEDGQIVRDFVYIDDVIAALFAAIERPASPQRCLDIGSGTPTTIHELALKIAAICHAPEPVVVAKFRDGDVRAASCDIEPAKRALDWLPQRALEDGLDALLGWIGEQPESGVGPGDHTPLTALLR

>WP_220691304.1 SDR family NAD(P)-dependent oxidoreductase [Mycolicibacterium holsaticum]

MPASVLITGGAGFIGSALARRVVQAGYDVAVMDILHPQVHAEHAAIDLPSSVRLFTGDVTHAPDCDAVLRLFKPAQIVHLAAETGTAQSLSQASRHGSVNVVGTTQLLDALSRCGHVPEQVVLASSRAVYGEGAWQSDGRVFYPPPRSHAQLAASIWDPQSPSGEPAVPLASNAARTQPKPTNIYAATKLAQEHVLAAWTASHDTGLSVLRLQNVYGPGQSLTNSYTGIVALFARLAREKQTLEVYEDGRIVRDFVYIDDVVEALFSAIAEPAPSSRCLDIGSGVATTIHELARQLATICEAPAPVVVAKFRDGDVRAAKCDIEQATKELRWHPKWSLEDGTRALLDWIGM

>WP_085269525.1 NAD-dependent epimerase/dehydratase family protein [Mycobacterium parmense]

MSASVLITGGAGFIGSALSRRLVTAGYDVAVLDVLHPQVHGDRAVLDLPSSVRFFTGDVTHAPDCDAVLRTFRPSQIVHLAAETGTAQSLSEATRHGSVNVVGTTQLVDALSRSAHVPDQFVLASSRAVYGEGAWQCGSAIFYPQPRSHGQLLAGIWDPRGPTGEPAVPLPSRAGRTEPRPTNIYAATKLAQEHILAAWTAAHDTNLSVLRLQNVYGPGQSLTNSYTGIVALFAQLARDRRPLEVYEDGRIVRDFVFIDDVVEALFAAIARPAPQPRCLDIGSGVATTIHGLATKLAALCDAPEPVVVGKFRDGDVRAARCDVEPAANEIDWHPKWALEDGLRVLLDWIGEQPIIRPV

>WP_276047875.1 SDR family NAD(P)-dependent oxidoreductase [Mycolicibacterium aichiense]

MSTSRSILITGGAGFIGSALARRLVEVGYEVAVMDILHQQVHAEGTVIDLPPSVRFFTGDVTHAPDCDAVLRLFKPSQIVHLAAETGTAQSLSEASRHGTVNVVGTTQLLDALSRCGHVPAQLVLASSRAVYGEGAWQSGNHVFYPASRSHAQLVAGDWDPHGPRGERAVPLASSAVQTEPRPTNIYAATKLAQEHILASWTAAHDTRLSVLRLQNVYGPGQSLTNSYTGIVALFARLSREKQPLEVYEDGQIVRDFVYIDDVVEAIFMAIESPAAPSRCVDIGSGVPTTIHELAQQLATICGAPDPVVVGKFRDGDVRAARCDIEHATNELGWRPKWSLEEGMRALLDWIGK

>WP_064282458.1 NAD-dependent epimerase/dehydratase family protein [Mycolicibacterium iranicum]

MSTSVLLTGGAGFIGSALARRLVHAGYDVAVMDVLHPQVHGMNTAIDLAPAVRLFTGDVTHAADWDAVLRLYRPDQIVHLAAETGTAQSLSEATRHGSVNVVGTTQLLDALSRAGHVPEQLVVASSRAVYGEGAWQAGAQIFYPAPRSHAQLVAGIWDPQGPNGDTALPLPSSAGRTEPRPTNIYAATKLAQEHILAAWTAAHDSNLSVLRLQNVFGPGQSLTNSYTGIVALFARLSLERQALEVYEDGRILRDFVFIEDVVEALFAAIRRPATGSRSVDIGSGVPTTIHQLARMTATICGAPEPVVVGKFRDGDVRAARCEIGCATKELDWQPKWQLEDGLDALVTWIGGQH

>MCV7299508.1 NAD-dependent epimerase/dehydratase family protein [Mycobacterium barrassiae]

MLITGGAGFIGSALSRRLVKAGHEVAVMDVLHPQVHTGYQAIDLPAAVRLFTGDVTHAPDLDAVLRLFRPTQIVHLAAETGTGQSLSEATRHASVNVVGTAQLLDALTRSKLVPDQLVLASSRAVYGEGAWQCGTGTFYPPPRSHAQLVAGIWDPRGPLDEPAVPVPSSADRTEPRPTNIYAATKLAQEHMLAAWVAAHDTNLSVLRLQNVFGPGQSLTNSYTGIVALFARLAREQQQLEVYEDGRIVRDFVFIDDVVDALFAALQKPALGSRCLDIGSGIGTTIHELANKVAAVCQAPEPVVVPKFRDGDVRAASCTIESANTELHWRPKTALDDGLQSLLDWIAEQPHVDPGQT

>WP_097942056.1 NAD-dependent epimerase/dehydratase family protein [Mycolicibacterium agri]

MLLTGGAGFIGCALARRLVQAGYDVAVMDVLHPQVHGTSRAVDLPPRVRLFTGDVTHGPDWDAVLRLFRPDQVVHLAAETGTAQSLAQATRHGSVNVVGTTALLDALSRFDLVPEQIVLTSSRAVYGEGAWRSGGHHFYPQPRSHAQLLAGVWDAQGPNGEPAEPVPSRAGETEPRPTNIYGATKLAQEHIMTAWTAAHDTNLTTLRLQNVYGPGQSLTNSYTGIVALFARLARGHQTLEVYEDGRIVRDFVYIDDVADALFAAIQRRATAMRLLDIGSGSATTIHELARKIAGLCDAPEPIVVPKFRDGDVRAASCSVQPASEVLGWCPQWTLDDGLNSLLKWIDSQDEATPSLND

>MBU9763064.1 NAD-dependent epimerase/dehydratase family protein [[Mycobacterium] fortunisiensis]

MLITGGAGFIGSALAHRLVEAGYEVAVVDVLHPQVHGDHATLELPSAVRFFTGDVTHAPDLDAVLRLFRPDQIVHLAAETGTAQSLSEATRHGSVNVVGTTQLLDALSRAGHVPDQLVLASSRAVYGEGAWQAGTEIFYPRPRSHAQLAAGSWDPTGADCAPAVPLPSSTAETQPRPTNIYAATKLAQEHLLAAWTAAHDTNLSVLRLQNVYGPGQSLTNSYTGIVALFGRLALAKQALEVYEDGRILRDFVYIDDVVEALYSAIYEPAATLRCLDIGSGVPTTIHELAQKMAILCDAPEPVVVGKFRDGDVRAARCDVEPTIKELGWRPKWGLEDGLRALLDWIGGQGATG

>WP_085167405.1 NAD-dependent epimerase/dehydratase family protein [Mycobacterium sherrisii]

MLITGGAGFIGSALSHRLVEAGYDVAAMDVLHPQVHAAGQAPDLPSAVRLFTGDVTHGPDCDAVLRLFRPDQVVHLAAETGTAQSLSQATRHGSVNVVGTTQLVDALSRAGLVPEQLVLASSRAVYGEGAWQAGSQVFYPGPRSHAQLAAGVWDPPGPDGEPAVALASCAARTEPRPSNIYAATKLAQEHILASWSAAHDTNLSVLRLQNVYGPGQSLTNPYTGVVPFFAQVSREQRPSDVYEDGRIVRDFVYIEDVVDALFAAVENPATGSRRLDIGSGIATTIHELARKIAATFGAPEPIVTAQFRDGDVRSASCDIGPAQSELHWRPKWSLDDGLRVLLDWIGERLESPSTASFPT

>WP_077089687.1 SDR family NAD(P)-dependent oxidoreductase [Mycobacterium rhizamassiliense]

MLVTGGAGFIGSALAHRLVTAGYDVAVMDVLHPQVHTGHQAIDLPSSARLFTGDVTHAPDCDAVLRLFRPSQIVHLAAETGTAQSLSHATRHGSVNVVGTTQLLDALSRATHVPDQLVLASSRAVYGEGAWQSGAQIFYPRPRSHAQLQAGAWDPVGPTGDAAVPLPSCAGRTEPRPTNIYAATKLAQEHLLAAWAAAHDTSLSVLRLQNVYGPGQSLTNSYTGIVTLFARLAREQRPLEVYEDGQIVRDFVYIDDVVEALFATIDRPAVEPRLLDIGSGVPTTIHELAHNIATLCEAPEPTVVAKFRDGDVRAAKCDIEAAKAELEWRPTWALEKGLLALLDWIGEQHELPSGATDHGPR

>WP_061559315.1 NAD-dependent epimerase/dehydratase family protein [Mycobacterium simiae]

MLITGGAGFIGSALSHRLVKAGHDVAVMDVLHPQVHAAGQAPDLPSAVRLFTGDVTHGPDCDAVLRLFRPDQIVHLAAETGTAQSLSHATRHGSVNVVGTTQLVDALSRAGLVPAQLVLASSRAVYGEGAWQCGSEVFYPGARSHAQLVAGVWDPPGPGGEPAVSLPSCAARTEPRPSNIYAATKLAQEHILASWSAAHDTNLSVLRLQNVYGPGQSLSNPYTGVVPFFAQVSREQRPSDVYEDGRIVRDFVYIEDVVDALFAVVENPATGSRRLDIGSGIATTIHELARKIAAHFGAPQPVITGRFRDGDVRAASCDIGPAQSELGWRPKWSLDDGLRVLLDWIGERLESASTASFPSK

>WP_163899515.1 SDR family NAD(P)-dependent oxidoreductase [Mycolicibacterium pulveris]

MPKSVLITGGAGFIGSALARRLVEDGYDVGVLDVLHPQVHGAHATLDLPASVRFFSGDVTHAPDCDVVLRLFKPAQVVHLAAETGTAQSLSEASRHGSVNVVGTTQLLDALSRCGYVPDQLVLASSRAVYGEGAWQAGSHIFYPPPRSHAQLAAGSWDPPSPSGEPAAPLASSAGRTEPRPTNIYAATKLAQEHIMAAWAAAHDTRLSVLRLQNVYGPGQSLTNSYTGIVALFARLARQQQILEVYEDGRIVRDFVYIDDVVEALFSAIEEPAGQARCVDIGSGVPTTIHELARQLASICDAPEPVVVPKFRDGDVRAARCDTEQAMKELNWRPKWSLDEGTRALLDWIGTLVPNGHR

>WP_115327552.1 NAD-dependent epimerase/dehydratase family protein [Mycolicibacterium gilvum]

MTTSVLITGGAGFIGSALARRLVDAGHDVAVMDVLHPQVHRKPCVVELPREVRLFTGDVTHAPDWDAVLRLFEPTQIVHLAAETGTAQSLSEATRHGSVNVVGTTQLVDALGRADFVPEHLVLASSRAVYGEGAWDTGAGVVYPPPRTHAQLAAGNWDPVGPDGVALLPLASAADRTEPRPTNIYASTKLAQEHILASWAAAHDTRLSVLRLQNVFGPGQSLTNSYTGIVALFARLSRARQPLEVYEDGRILRDFVYIDDVVDALLAALQRPRAKARCLDIGSGASTTIHELATMAARLCDAPDPVIVGKFRDGDVRAAKCDVSAAEDDLGWRPKWRLEDGLLALLDWVDDAGERTLRSGDLGVLTR

>WP_090342508.1 NAD(P)-dependent oxidoreductase [Mycolicibacterium malmesburyense]

MLVTGGAGFIGSALARRLVQGGHDVAVMDVLHPQVHGGSCEIDLPPSVRLFTGDVTHAPDWDAVLRLFRPSQIVHLAAETGTAQSLSHATRHGSVNVVGTAQLLDALSRWSLTPDQLVLASSRAVYGEGAWRCGAEVFYPPPRSHAQLLSGDWDPVRPSAERSVPLPSSAADTEPRPTNIYAATKLAQEHIMRAWASAHDVNISVLRLQNVYGPGQSLTNSYTGIVTLFARLAREHHALEVYEDGRIVRDFVYIADIIDAMYAAVEKPAAGLRLVDIGSGVATTIHDLARKVAAICGAPEPTVVGKFRDGDVRHASCTIELAEAELDWRPRWSLEDGLHALLATTGLQPD

>WP_006245484.1 NAD-dependent epimerase/dehydratase family protein [Mycolicibacterium tusciae]

MLITGGAGFIGSLLSRRLVDAGYDVAVMDVLLPQVHGARQAVDLPPSVRFFTGDVTHAPDWDAVLRLFRPSQVVHLAAETGTGQSLSHATRHGLVNVVGTTQLLDALTRAALVPDQLVVASSRAVYGEGAWQSGPHMFYPRARSHAQLAAGIWDPSGPEGEPAVPLPNRAGHTEPRPTNIYGATKLAQEHLLTAWTAAHDTSLSVLRLQNAYGPGQSLTNPYTGVVPFFARLSREGRPSEVYEDGRIVRDMVYIDDVVDAVFATIEKPATEPRCLDIGSGVGTTIHDLARKIAAVFDAPEPVVVPKFRDGDIRAASCDIEPAKNELHWQPTWTLDNGLHALLEWIAKQPEQPSHTSDHP

>WP_144208697.1 NAD-dependent epimerase/dehydratase family protein [Mycobacterium tilburgii]

MLITGGAGFIGSALSHRLVKAGYDVAVMDVLHPQVHAQGQAPDLPSAVRLFTSDVTHGPDCDAVLRLFRPIQIVHLAAETGTAQSLSQATRHGSVNVVGTTQLVDALSRAEWVPQQLVLASSRAVYGEGAWQSGTQVFYPGPRSHAQLVAGVWDPPGSGGEPAVSLPSCAARTEPRPSNIYAATKLAQEHILASWCAAHGSNLSVLRLQNVYGPGQSLSNPYTGVVPFFARVSREQRPSNVYEDGRIVRDFVYIEDVVDALFAAVQNPAPGSRRLDIGSGVTTTIHELARKIAANFGAPEPVVTAQFRDGDVRAASCDIGPAQSELHWRPKWSLDDGLRVLFDWIVRQLESPSTATFPP

>WP_082695000.1 NAD-dependent epimerase/dehydratase family protein [Mycobacterium lehmannii]

MLITGGAGFIGSALARRLVRSGYEVGVLDVLHPQVHSGSQPLDLPESARLFTGDVTHGPDLDAVLRLFRPTQVVHLAAETGTGQSLAEATRHGSVNVLGTTQLLDALTRSNYVPAQFVLASSRAVYGEGAWQSASGTFYPPPRSHAQLQAAVWDPRGPAGEDARPLPSCAGQTEPRPTNIYAATKLAQEHILSAWTAAHDTNLSVLRLQNVYGPGQSLANSYTGIVTLFARLAGEGQPLEVYEDGNIIRDFVFIDDVADALFAAVQAPANQQRCVDIGSGIGTTILELAQKIAAICDSPEPTVVSKFRDGDVRAASCTVEAARVELNWSPTWSLDQGLHALLEWIASKHLFPDQGRSVVNSTIAPSQSGYWDRAR

>WP_046183087.1 NAD-dependent epimerase/dehydratase family protein [Mycobacterium nebraskense]

MLITGGAGFIGSALAHRLVKVGCDVAVMDVLLPQVHDGRAIDLPPSVRFFTGDVTHAPDWDAVLRLFRPTQVVHLAAETGTAQSLTEATRHGAVNVVGTTQMLDALGRKGLVPEQFVLASSRAVYGEGAWLSGGEVFYPPPRSHAQLVAGIWDPPGPAGEPAVPLPSSAGRTEPRPTNVYGATKLAQEHLLAAWAAAHDTNLSVLRLQNAFGPGQPLSNPYTGVVPFFARLSREGRASEVYEDGRIVRDLVFIDDVVDAVFAAVQQPAKGSRCLDIGSGIGTTIHELARKIAAAFDAPEPVLVPKFRDGDVRAASCDIQPAMDELGWRPKWTLDDGLRVLLEWIGSLPESGPAASDSVGGRPVEHAGHR

>WP_083164476.1 GDP-mannose 4,6-dehydratase [Mycobacterium aquaticum]

MSKSVLISGGAGFIGTALSRRLVEAGYDVAVMDVLLAQVHGDGKALDLHPSVRFFTGDVTHAPDWDAVLRLCRPSQVIHLAAETGTAQSLSQATRHGLVNVVGTTQLLDALSRAALVPEQIVVASSRAVYGEGAWQSGDEIFYPRPRSHAQLQAGIWDPEGPTGEPAVPLPSRAGVTETRPTNVYGATKLAQENLLTAWTSAHDTGLSILRLQNAYGPGQSLTNPYTGVVPFFARLSRQLRASEVYEDGRIVRDLVYIDDVIDALFTSVVNPAAQSRCLDIGSGHGITIHELAKKIADVFGAPEPVVVGKFRDGDVRAASCDIEPTLSDLQWRPKWTLDDGLRSLLDWIGTQAEAGAGDQAEYLDRVGVERAR

>MCV7309389.1 NAD-dependent epimerase/dehydratase family protein [Mycobacterium paraffinicum]

MLITGGAGFIGTLLSRRLVEAGYDVALMDVLLPQVHGERPTLKLARSVRLFTGDVTHAPDWDAVLRLFRPSQIVHLAAETGTAQSLSQATRHGSVNVVGTTQLLDALSRVDLVPDQLVLASSRAVYGEGAWRSGSHVFYPRPRSHAQLLAGVWDPVGPTGEPAVPIPHRAGETEPRPTNIYGATKLAQEHLLAAWAAAHDTKLSVLRLQNAYGPGQSLTNPYTGVVPFFARLSREKLPSEVYEDGRIVRDLVYIDDVVDALFAAVQQPAAESRCLDIGSGNGITIHQLARKIAAIFDAPEPVVVPKFRDGDIRAASCDVGPASGELQWRPKWSLDDGLRSLLDWIDEQSDVPSAAGDRAPSSGSSRR

>WP_163702120.1 NAD-dependent epimerase/dehydratase family protein [Mycolicibacterium sarraceniae]

MLITGGAGFIGSALARRLVQAGYDVAVLDVLHPQVHGEHATLDLPQSVRMFIGDITHAPDMDATLRLFKPAQIVHLAAETGTAQSLSEATRHGSVNVVGTTQLLDALTRAGHVPEQLVLASSRAVYGEGAWRSGTHTFHPSPRSHADLMAGLWDPQGPTGDSAVPLPSRAGSTDPRPTNIYAATKLAQEHILTAWTAAHDTTCSVLRLQNVYGPGQSLTNAYTGIVALFARLALQRQVLDVYEDGRILRDFVYIDDVVEALFAAVEKPAAPARAVDIGSGVPTTIHELANTLAAICGAPDPVVGGKFRDGDVRAARCDIESAIDDLDWRPTWALKDGLRALLVWIADQPGLAPD

>WP_036430385.1 GDP-mannose 4,6-dehydratase [Mycolicibacterium mageritense]

MSRSVLISGGAGFIGAALSRRLVEAGYDVAVMDVFLPQVHAGGQMFKLSPAVRLFTGDVTHAPDWDAVLGLFRPDQVIHLAAETGTAQSLSQATRHGAVNVVGTTQLLDALSRAGLVPEQIVVASSRAVYGEGAWQSGSQTFYPRPRSHAQLVAGIWDPQGPTGDPAVPLPSRADQTEARPTNVYGATKLAQENLLTAWTSAHDTGLSILRLQNAYGPGQSLSNPYTGVVPFFARLSRELRASEVYEDGRIVRDLVYIDDVIDALFAAVVKPAAESRCVDIGSGHGITVHELAQKIAAVFDAPEPVVVGKFRDGDVRAASCDIGLAQRELDWRPKWNLDDGLRSLLDWIGSQSESGPVDHAAQLDQVGVGRAR

>WP_087072652.1 GDP-mannose 4,6-dehydratase [Mycobacterium dioxanotrophicus]

MSKSVLISGGAGFIGTALSRRLVEAGYDVAVMDVLLAQVHGDGKALDLHPSVRFFTGDVTHAPDWDAVLRLCRPSQVIHLAAETGTAQSLSQATRHGLVNVVGTTQLLDALSRADLVPEQIVVASSRAVYGEGAWQAGDEVFYPRPRSHAQLQAGVWDPQGPTGEPGVPLPSRAGVTETRPTNVYGATKLAQENLLTAWTSAHDTGLSILRLQNAYGPGQSLTNPYTGVVPFFARLSRQLRASEVYEDGRIVRDLVYIDDVIDALFTSVVNPAVQSRCLDIGSGHGITIHELAKKIAAVFGAPEPVVVGKFRDGDVRAASCDIEPTLSDLQWRPKWTLDDGLRSLLDWIGTQTEAGAEDGAEYLDRVGVERAR

>WP_048630983.1 NAD-dependent epimerase/dehydratase family protein [Mycolicibacterium aurum]

MTASVLITGGAGFIGSALARRLVDSGCDVAVMDVLHPQVHPRHTVVPIPSEARLFTGDVTHAPDWDAVLRLFQPSQIVHLAAETGTAQSLSAATRHGSVNVVGTTQLVDALSRAGYIPEHLILASSRAVYGEGAWGFGADVFYPPPRTHAQLSAGHWDPKGPDGVSAAPLASAASRTEPRPTNIYASTKLAQEHILAAWTAAHGAGLSILRLQNVFGPGQSLTNSYTGIVALFARLSRARQSLEVYEDGRILRDFVYIDDVVDALFAAVRQPSTDARWFDVGSGVSTTIHELAAMTARLCGAPDPRVVGKFRDGDVRAAKCDIRAAVDELGWRPEWALEDGLLALLDWIDNQAERTDVPGDLSVLTS

>MBU8814602.1 GDP-mannose 4,6-dehydratase [Mycolicibacterium goodii]

MLITGGAGFIGSLLSRRLVEAGYEVAVTDVFLPQVHGDRRKTELPSSVRLFTSDVTHAPDWDAVLRLFRPDQVVHLAAETGTAQSLSQATRHGAVNVVGTTQLLDALSRADLVPEQIVVASSRAVYGEGAWQSGSEVFYPKPRSHAQLAAGIWDPVGPTDAPAVHLPSRAGETQPRPTNIYGATKLAQEHLLAAWTSAHDTALSVLRLQNAYGPGQSLTNPYTGVVPFFARVSREGRASEVYEDGLIIRDLVYIDDVIEALFASVDRPAAESRYLDIGSGIGVTIHDLARKIAAAFDAPEPVVVGKFRDGDIRAASADIEPAQRELDWTPKWGLDEGLGALLDWINEQHERSSRDN

>WP_064412240.1 GDP-mannose 4,6-dehydratase [Mycolicibacterium novocastrense]

MSASVLITGGAGFIGSALSRRLVEVGYEVAVMDMLLPQVHGDRPTINLANSVRFFTGDVTHAPDLDAVLRLFRPTQVVHLAAETGTGQSLAHATRHGSVNVVGTAELLDAMSRLAIVPEHLVLTSSRAVYGEGAWQSGGETFYPPPRSHAQLEAGVWDPPGPTGEPALPLPHCAGQTEPRPTNIYGATKLAQEHMLTAWAAAHDTNLSVLRLQNAFGPGQSLTNPYTGVVPFFARLSREQRPSEVYEDGQIIRDLVYIDDVIDAVFATVQNPATKARCLDIGSGTRTTILELAQKIAAVFNAPEPIVVGKFRDGDIRAASSDIDAAKNELQWQPKWSLDDGLRALLDWISRQPQLS

>WP_115281115.1 NAD-dependent epimerase/dehydratase family protein [Mycolicibacterium tokaiense]

MPISVLITGGAGFIGSALAHRLVQAGYDVTVLDVLHPQVHGAELALTLPPSARLLTGDVTHAPDLDAILRLVKPDQVVHLAAETGTAQSLSQASRHGAVNVVGTTQLLDALGRTAHVPRHLVLASSRAVYGEGTWIADGEVFYPRPRTHAQLVAGQWDPVSPNGRPAHPRASRAGHTEPRPTSIYAATKLAQEHIMAAWAAAHDTAFSILRLQNVYGPGQSLTNSYTGIIALFARLAREQHTVEVYEDGCIVRDFVYIEDVADALFAAVQRPPTGQDRCVDIGSGTPTTIDQVARKIAGLCAAPEPVVVGRFRDGDVRAARCDIGSAMDQLDWSPKWTLDDGLAALLRWIDERPAPQRT

>WP_193489973.1 NAD-dependent epimerase/dehydratase family protein [Mycolicibacterium murale]

MPISVLITGGAGFIGSALAHRLVQAGYDVTVLDVLHPQVHGAELALTLPPSARLLTGDVTHAPDLDAILRLVKPDQVVHLAAETGTAQSLSQASRHGAVNVVGTTQLLDALGRNAHVPRHLVLASSRAVYGEGTWTADGEVFYPRPRTHAQLVAGQWDPVSPNGRPAHPRASRAGHTEPRPTSIYAATKLAQEHIMAAWAAAHDTAFSILRLQNVYGPGQSLTNSYTGIIALFARLAREQRTVEVYEDGCVVRDFVYIEDVADALFAAVQRPPTGQDRCVDIGSGTPTTIDQVARKIAGLCAAPEPVVVGRFRDGDVRAARCDIGSAMDQLDWSPKWTLDDGLAALLRWIDERPVPQRT

>WP_231250540.1 NAD-dependent epimerase/dehydratase family protein [Nocardioides furvisabuli]

MPSSTQAGGRVLVTGGAGFIGTTLARELADSAEQWVVLDNLHPQVHPGSQPPADLPDSVDLRIGDVTSAEDLDAVVADLRPDTVVHLAAETGTAQSLSESTRHGMVNVVGTTQLLDALTRAGHVPGHVVLTSSRAVYGEGVWRNADGSTFQPGLRTHAQLEAGTWDHGDGAAHVPNSVAGTHPNPTNVYGATKLAQEQILAAWTGSHDTRLSVLRLQNVYGPRQSLSNPYTGIVSLFSRLAREGTSIPLYEDGEITRDFVYIDDVVSALVAAISRRPADHVRTVDVGSGVRTTIGDLAREVARYHSAPEPHVTGQYRDGDVRHASCTVEDTVRRLDWEPRWSLRDGVAGLQEWIATQLD

>WP_224276127.1 NAD-dependent epimerase/dehydratase family protein [Nocardioides lacusdianchii]

MSSSTQAGGRVLVTGGAGFIGTTLARELADSAEQWVVLDNLHPQVHPGSQPPADLPDSVDLRIGDVTSAEDLDAVVADLRPDTVVHLAAETGTAQSLSESTRHGMVNVVGTTQLLDALTRAGHVPGHFVLTSSRAVYGEGVWRNADGSTFQPGLRTHAQLEAGTWDHGDGAAHVPNSVAGTHPNPINVYGATKLAQEQILAAWTGSHDTRLSVLRLQNVYGPRQSLSNPYTGIVSLFSRLAREGKSIPLYEDGEITRDFVYIDDVVSALVAAISRRPADHMRTVDVGSGVRTTIGDLAREVARYHSAPEPHVTGQYRDGDVRHASCTVEDTVRRLDWEPRWSLRDGVAGLQEWIGTQLD

>WP_236397146.1 NAD(P)-dependent oxidoreductase [Nocardioides potassii]

MSSSQQAGGRVLVTGGAGFIGTTLARQLADAAEQWVVLDNLHPQVHPGSQPPADLPDSVDLRVGDVTSADDLDAVVADLRPDTVVHLAAETGTAQSLSESTRHGMVNVVGTTQLLDAFTRAGHVPGHFVLTSSRAVYGEGVWRNPDGTTFQPGLRTHAQLESGKWDHGDGAAHVPNTVAGTHPNPINVYGATKLAQEQILAAWTGSHDTRLSVLRLQNVYGPRQSLSNPYTGIVSLFSRLAREGQSIPLYEDGEITRDFVYIDDVVSALVAAIAHKPADHMRTVDVGSGVRTTIGDLAREVARYHSAPEPHVTGQYRDGDVRHASCTVEDTIRSLDWQPRVSLRDGVAGLQEWIATQLD

>WP_091195081.1 NAD(P)-dependent oxidoreductase [Nocardioides alpinus]

MPSPSQAGGRVLVTGGAGFIGTTLARQLADSAEQWVVLDNLHPQVHPGSEPPADLPGSVDLRVGDVTSADDLDAVVADLRPDTVVHLAAETGTAQSLSESTRHGMVNVVGTTQLLDSLTRAGHVPSHFVLSSSRAVYGEGVWRNPDGTTFQPGLRTHAQLEAGTWDHGDGAAHVPNSVAGTHPNPINVYGATKLAQEHILAAWTGSHDTRLSVLRLQNVYGPRQSLSNPYTGIVSLFSRLAREGQSIPLYEDGDITRDFVHIDDVVSALVAAIAHKPADHMRTVDIGSGVRTTIGDLAREVARYHSAPEPHITGQYRDGDVRHASCAVEDTVRRLDWEPRVSLRDGVAGLQEWIATQLD

>WP_191193581.1 NAD-dependent epimerase/dehydratase family protein [Nocardioides cavernae]

MSSQQAGGRVLVTGGAGFIGTTLAREIADSAEQWVVVDNLHPQVHPGSEPPADLPDAVDLRVGDVTSAEDLDAVVADLRPDTVVHLAAETGTAQSLSESTRHGMVNVVGTTQLLDALTRAGHVPSHVVLTSSRAVYGEGLWRNPDGSTFQPGLRTHAQLSAGRWDHGDRAAHVPNSVTGTHPSPINVYGATKLAQEQILAAWTGSHDTRLSVLRLQNVYGPRQSLSNPYTGIVSLFSRLAREGQSIPLYEDGDITRDFVYIDDVVSALVAVIARKPADHMRTVDVGSGVRTTIADLAREIARYHSAPEPHVTGQYRDGDVRHASCTVEDTVRQLGWEPRWSLRDGVAELQEWIATQLD

>WP_107767859.1 NAD-dependent epimerase/dehydratase family protein [Nocardioides terrigena]

MTSDPTLRASPRPGSVLVTGGAGFIGCALAERLAPLADRWVVLDNLHPQVHAVSERPAALHDAAQLVVGDVTDPAAWDSLLTGFRPDVVVHLAAETGTAQSLSESSRHGRVNVVGTTELLDGLTRAGVVPAQLVLTSSRAVYGEGTWRRADGSRFQPGARTHAQLAAGLWDFPDAEHLPNSAAETVPAPSSVYGSTKLAQEHILSSWAGSHDTSLSVLRLQNVYGVGQSLTNPYTGIVSLFSQMARGGRSIPLYEDGEITRDFVFIDDVADALVAAIASPPERFRLVDVGTGTRTTIRDLARTVADFHGAPAPHVTGQFRDGDVRHAACDITETLGALDWSPRWGLADGIAALQGWIAQQAAAEAPTSG

>WP_129477608.1 NAD(P)-dependent oxidoreductase [Nocardioides glacieisoli]

MSSSRLNQAGGRVLVTGGAGFIGTTLARQLADSAEQWVVLDNLHPQVHPGSQPPADLPDSVDLRIGDVTSADDLDAVVADLRPDTVVHLAAETGTAQSLSESTRHGMVNVVGTTQLLDSLTRAGHVPGHFVLTSSRAVYGEGVWRNADGSTFQPGLRTHAQLEAGQWDHGDGAAHVPNTVAGTHPNPINVYGATKLAQEQILSAWTGSHDTRLSVLRLQNVYGPRQSLSNPYTGIVSLFSRLAREGESIPLYEDGDITRDFVYIDDVVSALVAAIAHQPADHMRTVDVGSGVRTTIGDLAREIARYHSAPEPHVTGQYRDGDVRHASCTVEDTLRSLDWQPRVSLRDGVAGLQEWIATQLD

>WP_129426565.1 NAD(P)-dependent oxidoreductase [Nocardioides zhouii]

MSTSHQAGGRVLVTGGAGFIGTTLARELADSAEQWVVLDNLHPQVHPGSQPPDDLPASVDLRVGDVTSADDLDAVVADLRPDTVVHLAAETGTAQSLSESTRHGMVNVVGTTQLLDALTRAGHVPEHFVLTSSRAVYGEGVWRNADGSTFQPGLRTHAQLEAGKWDHGDGAAHVPNTVAGTHPNPINVYGATKLAQEQILSAWTGSHDTRLSVLRLQNVYGPRQSLSNPYTGIVSLFSRLAREGESIPLYEDGDITRDFIYIDDVVSALVAAIAHQPADHMRTVDVGSGVRTTIGDLAREIARYHSAPAPHVTGQYRDGDVRHASCTVETTLRHLDWQPRVSLRDGVAGLQEWIATQLD

>WP_224749279.1 NAD(P)-dependent oxidoreductase [Nocardioides hwasunensis]

MSSSQRAGGRVLVTGGAGFIGTTLARELADSAEQWVVLDNLHPQVHPGSQAPADLPDSVDLRVGDVTSADDLDATVADLRPDTVVHLAAETGTAQSLSESTRHGMVNVVGTTQLLDSLTRAGHVPSHFVLTSSRAVYGEGVWRNVDGSTFQPGLRTHAQLEAGTWDHGDGAAHVPNTVDGTHPSPINVYGATKLAQEQILSAWTGSHDTRLSILRLQNVYGPRQSLSNPYTGIVSLFSRLARDGQSIPLYEDGEITRDFVHIDDVVSALVAAIAHKPADHVRTVDVGSGVRTTIGDLAREIAAYHSAPDPHVTGQYRDGDVRHASCDVEVTLRTLDWKPRVSLREGVAGLQEWIATQL

>WP_066041785.1 SDR family NAD(P)-dependent oxidoreductase [Herbiconiux solani]

MPDLSSHRVVVTGGAGFIGCALSQRLAGQVAKWVVIDSLHPQVHPVQERPADLHEAAELVVGDVTDPAVWDAVLADIQPTIVIHLAAETGTAQSLDEATRHSHVNVVGTTQMTDALGRHGIVPERFLLSSSRAVYGEGAWQKSDGEVYYPGQRSHAQFEAGQWDFPDATPLPSIASRTVPAPTSVYGATKLAQEHVLAAWVNARDTSLTALRLQNVYGPGQSLSNPYTGIVSLFSQLAMKGQSIPIYEDGKITRDFVYIDDVADAFVAALGKPAEPGVLVRDVGSGVGTTILQLAESIARFHGAPEPHITGKYRDGDVRHAECSIEDTLATFDWTPEWDVDRGVAGLQEWIAAQQA

>MBD3916651.1 NAD(P)-dependent oxidoreductase [Nocardioides hwasunensis]

MSLMSSSQRAGGRVLVTGGAGFIGTTLARELADSAEQWVVLDNLHPQVHPGSQAPADLPDSVDLRVGDVTSADDLDATVADLRPDTVVHLAAETGTAQSLSESTRHGMVNVVGTTQLLDSLTRAGHVPSHFVLTSSRAVYGEGVWRNVDGSTFQPGLRTHAQLEAGTWDHGDGAAHVPNTVDGTHPSPINVYGATKLAQEQILSAWTGSHDTRLSILRLQNVYGPRQSLSNPYTGIVSLFSRLARDGQSIPLYEDGEITRDFVHIDDVVSALVAAIAHKPADHVRTVDVGSGVRTTIGDLAREIAAYHSAPDPHVTGQYRDGDVRHASCDVEVTLRTLDWKPRVSLREGVAGLQEWIATQL

>WP_129399646.1 NAD(P)-dependent oxidoreductase [Nocardioides oleivorans]

MAAPGTAGGRVLVTGGAGFIGTTLARELADSAEQWVVLDNLHPQVHPGSEPPADLPASVDLRVGDVTSADDLDAVVADLRPDTVVHLAAETGTAQSLSESTRHGMVNVVGTTQLLDALTRAGHVPSHFVLTSSRAVYGEGVWRNVDGSTFQPGLRTHAQLESGAWDHGDGAAHVPNTVLGTHPSPINVYGATKLAQEQILSAWTGSHDTRLSVLRLQNVYGPRQSLSNPYTGIVSLFSRLAREGRSIPLYEDGEITRDFVHIDDVVSALVAAIAHKPADHVRTVDVGSGVRTTIGDLAREVARYHDAPEPHVTGQYRDGDVRHASCDVEVTLRQLDWQPRVSLRDGVAGLQEWIATQLD

>WP_090968298.1 NAD(P)-dependent oxidoreductase [Nocardioides exalbidus]

MSSSQQAGGRVLVTGGAGFIGTTLARELADSAEQWVVLDNLHPQVHPGSEPPADLPGSVDLRVGDVTSADDLDAVVAELRPDTVVHLAAETGTAQSLSESTRHGMVNVVGTTQLLDSLTRAGHVPSHFVLTSSRAVYGEGVWRNVDGSTFQPGLRTHAQLESGKWDHGDGAAHVPNTVLGTHPNPINVYGATKLAQEQILSAWTGSHDTRLSVLRLQNVYGPRQSLSNPYTGIVSLFSRLAREGQSIPLYEDGEITRDFVHIDDVVSALVAAIAHKPADHMRTVDVGSGIRTTIGDLAREVARYHSAPEPHVTGQYRDGDVRHASCDVEVTLRQLDWRPEVGLREGVAGLQEWIAGELGR

>WP_028445727.1 NAD-dependent epimerase/dehydratase family protein [Chitinimonas koreensis]

MNSNNTQAFVEGKTVLVTGGAGFIGCALAKRLAAHASRYVVVDNLHPQVHASSERPSDLHQAAELIVGDVTLASTWDSLLTHCSPDIVIHLAAETGTGQSLTEGTRHSMVNVVGTSQMTDALGRHGHTPSHILLTSSRAVYGEGAWKSATGDVFYPGQRDRSQLEAAQWDYPDGTSLPARADVTIPAPSSIYGATKLTQEHILSAWANAQKVALTILRLQNVYGPGQSLTNSYTGIVALFSRLARAGQSIPLYEDGNVTRDFVFIDDVADAIVAALATATSAKRVLDVGSGTAATIRQMAELVACYYSAPSPHVCGKFRDGDVRHAACDIDRTISQLPWQPKWNLEAGVAALQQYIASINA

>WP_179619190.1 NAD(P)-dependent oxidoreductase [Nocardioides cavernae]

MASSQQAGGRVLVTGGAGFIGTTLARELAEQAEQWVVLDNLHPQVHPGSQPPADLPEAVDLRIGDVTDAGDLDAVVADLRPDTVVHLAAETGTAQSLSESTRHGMVNVVGTTQLLDALTRAGHVPAHVVLTSSRAVYGEGVWRNPDGSTFQPGLRTHAQLEAGRWDHGDGAAHVPNTVAGTHPHPINVYGATKLAQEQILSAWTGSHDTRLSVLRLQNVYGPRQSLSNPYTGIVSLFSRIAREGGSIPLYEDGDITRDFVHIDDVVSALVAAIAHKPADHLRTVDVGSGVRTTIGDLAREIAAYHSAPEPHVTGQYRDGDVRHASCDVEMTLRALDWQPRVSLRDGVAGLQEWIATQLD

>WP_205528710.1 SDR family NAD(P)-dependent oxidoreductase [Desertimonas flava]

MATILITGGAGFIGCRLSVALLAAGDDVAVLDNLHPQVHPTRTVPRNLPDEVRFVPGDVAAASSWAAVLATVRPDAIVHLAAETGTGQSLTEPTRHANVNVLGTAAMLEALEHSPHRPGHIVLASSRAVYGDGAWITNEGTVFYPGQRSAAQLDAGRWDHSAPDGSPARPIPSVAGITVPEPTNIYAATKLAQEHLLRSWTQARQVDLSILRLQNVYGPGQSVTNSYTGVLTFFARTALEGRVIDVYEDGEIVRDFVFVDDVASALAASVRRPANRLVDIGSGRPTTIGAVARIVAEACAAPPPVVSGRYRHGDVRAASCNIDTAAGSLGYEPTKPLTDGIAAVLDWMPTAAEFRAR

>WP_201138989.1 dTDP-L-rhamnose 4-epimerase [Paraburkholderia domus]

MMDAELSLVDGKNVLVTGGAGFIGCAISKRLAPRAGRCVVVDNLHPQIHAQAVRPAALDARAELVVADVTEAATWDALLSDFKPQIIVHLAAETGTGQSLTEASRHALVNVVGTTRLTDALTKHGVVVEHILLTGSRAVYGEGAWQSDDGQIVYPGQRGRTQLEAAQWDFPGMTMLPSRADRTEPRPTSVYGATKLAQEQVLRAWALATKIPLSILRLQNVYGPGQSLTNSYTGIVALFSRLAREKKVIPLYEDGRVTRDFVSIDDVADAIVAMLERQPQPLSILDIGSGAAASILDMARIVAAHYGAPEPTVTGAFRDG

DVRHAACDLSVTLEQLDWKPQWSLERGVAELQNWIAQELDRKN

>WP_236665542.1 NAD(P)-dependent oxidoreductase [Nocardioides baculatus]

MSSSQQAGGRVLVTGGAGFIGTTLARQLADSAEQWVVLDNLHPQVHPGSQPPADLPDSVDLRVGDVTSAHDLDAVVADLRPDTVIHLAAETGTAQSLAESTRHGMVNVVGTTQLLDALTRAGHVPGHFVLTSSRAVYGEGVWRNPDGSTFQPGLRTHAQLEAGKWDHGDGAVHVPNTVAGTHPNPINVYGATKLAQEQILSAWTGSHDTRLSVLRLQNVYGPRQSLSNPYTGIVSLFSRLAREGESIPLYEDGDITRDFVHIDDVVSALVAAIAAPPVDHMRTVDVGSGVRTTIGDLAREIARYHSAPEPHVTGQYRDGDVRHASCTVEDTVRTLDWEPRVSLRDGVAGLQDWIATQLD

>TFD86362.1 SDR family NAD(P)-dependent oxidoreductase [Cryobacterium serini]

MSSTVLVTGGAGFIGCALSQKLAELYDRWVVLDSLHPQVHATGERPADLHDSAELVVGDVTDAAVWDHVLESVRPSVIIHLAAETGTAQSLHEASRHAQVNVVGTTQMLDALGRANQRPAHIVLSSSRAVYGEGRWENAHGDSFQPGQRSHAQFEAGLWDFVGAHGLPSVAAETVPAPTSVYGATKIAQENILAAWGGSHDVRVSILRLQNVYGPGQSLINSYTGIVTLFSQWARDGKTIPLYEDGQIVRDFVFIDDVADAFVAVLKRESTVPMPVLDVGSGVATTIAQLAQAIADYYGAPTPVVNGAYRDGDVRYAACTIRDTLRELDWAPQWSVERGLAALQDWIGSELKSGAGISRG

>WP_218590154.1 NAD-dependent epimerase/dehydratase family protein [Pseudonocardia oceani]

MSARSVLVTGGAGFIGSRLAARLHEAGDRVSVVDSLHPQVHATGEWPALLPAGVERVLGDVTDPALWDALLPRTRPDVVVHLAAETGTGQSLTEASRHGMVNVVGATRLLDGLTRHRRVPRALVLPSSRAVYGEGAWADAVGRTCYPPARDAGALEAGRWDPELQEVPARPLAHSASTTEPRPTNIYAATKLAQEHLLGAWAAAHGCTLSVLRLQNVYGPGQSLTNPYTGIVTLFARLGLHGEGIEVFEDGRILRDFVYVDDVVSALVAASALDDGARTTVDVGSGEPVTLLDLAERIAGLCGAPAPRVSGRYRAGDVRAAHADLAAAAERLGYHPTVTLDSGLRRLLTWIAAETGVRPPAEVLVDGRS

>WP_022884968.1 NAD-dependent epimerase/dehydratase family protein [Glaciibacter superstes]

MSAVGTVLVTGGAGFIGCALSQKLAGSAERWVVLDSMHPQVHGDTVRPTELYDDAEMIVGDVTDAAAWDTLLETVRPDVVIHLAAETGTAQSLHEASRHSRVNVVGTTEMLDAFGRAGIVPGLILLSSSRAVYGEGQWRSAAGELYVPGQRSHAQFENAQWDFPDSEPVASSAARTWPTPTSVYGATKLAQEHILSAWVEAHDSALVILRLQNVYGPGQSLINSYTGIVSLFSQLAADGKSIPIYEDGKITRDFVYIEDVADAFVALLKHAPKQGLVRYDVGSGVGTTIQELAQHIADFHHAPAPHVTGAYRDGDVRYASCDIEPTTAALGWQPQWSVERGVAGLQDWIAAQA

>WP_186204236.1 dTDP-L-rhamnose 4-epimerase [Burkholderia gladioli]

MSEINTSWVDGKRILVTGGGGFIGCAISKRLAARASRYVVMDNLHPQIHAQAVRPDALHDKAELVIGDVTEAEAWDKVLAEFRPDIVIHLAAETGTGQSLTEASRHALVNVVGTTRMTDALVRHGITVERILLTSSRAIYGEGAWRKDDGSVVYPGQRGRAQLEAAQWDFPGMTMLPSRADRTEPRPTSVYGATKLTQEHVLSAWTLATKTPLSILRLQNVYGPGQSLTNSYTGIVALFSRLAREKKVIPLYEDGQVTRDFVSIDDVADAIVAALARDPAPYAVFDIGSGQATSILQMARVVAGYYGAPEPQVNGAFRDGDVRHAACDLSESLANLAWTPQYSLERGISELQTWIAEELNRKN

>ABM51687.1 NAD-dependent epimerase/dehydratase family protein [Burkholderia mallei SAVP1]

MMSDVNASLVDGKKILVTGGAGFIGCAISERLAARASRYVVMDNLHPQIHASAVRPGALHEKAELVVADVTDAGAWDALLSDFQPEIIIHLAAETGTGQSLTEASRHALVNVVGTTRLTDALVKHGIVVEHILLTSSRAVYGEGAWQKDDGTIVYPGQRGRAQLEAAQWDFPGMTMLPSRADRTEPRPTSVYGATKLAQEHVLRAWSLATKTPLSILRLQNVYGPGQSLTNSYTGIVALFSRLAREKKVIPLYEDGNVTRDFVSIDDVADAIVATLVRTPEALSLFDIGSGQATSILDMARIIAAHYGAPEPQINGAFRDGDVRHAACDLSESLANLGWKPQWSLKRGIGELQTWIAQELDRKN

>WP_024744692.1 SDR family oxidoreductase [Xanthomonas oryzae]

MQKAVIVTGGAGFIGCALSGQLKAFGLPVVAIDNLHPQIHAESKRPEALDEAAHLHIGDVTEENTWGQVLENWQPTVVVHLAAETGTGQSLTEATRHAHVNVVGTTAMLDAFSARKLVPEHVLLASSRAVYGEGAWLDANGTTFYPPPRSHEVLARSQWNPLSPSGGGAASPLSHRADTVFPNPTSVYGATKLAQEHILAAWCGAMQVPLSVFRLQNVYGPGQSPFNSYTGIITLFHRMARKAQTLEIYEDGEIGRDFVFIDDVVVALMAGLRQPPAGLRTLDVGSGVVTTIAEAAKSIAAMHGAPDPQISGKFRDGDVRWAVADGAPLEQSLGVQARINFQEGANRVGEWLIARGYA

>WP_301796085.1 dTDP-L-rhamnose 4-epimerase [Burkholderia oklahomensis]

MSDVNASLVAGKKVLVTGGAGFIGCAISKRLAPRASRYVVMDNLHPQIHERAVRPDALDEKAELVVADVTDADAWDALLSDFQPDIVVHLAAETGTGQSLTEASRHAHVNVVGTTRLTDALVKHRVTVEHILLTSSRAIYGEGAWQKDDGTIVYPGQRGRAQLEAAQWDFPGMKMLPSRADRTEPRPTSVYGATKLAQEHVLRAWTLATKTPLSILRLQNVYGPGQSLTNSYTGIVALFSRLAREKKVIPLYEDGNVTRDFVSIEDVADAIAAALMRRPEPLSVFDIGSGHATSILEMARVVAEHYGAPEPQVNGAFRDGDVRHAACDLSESLANLAWKPQWSLARGIGELQTWIAQELDRKN

>WP_301626321.1 NAD-dependent epimerase/dehydratase family protein [Curtobacterium flaccumfaciens]

MNTTKTVLITGGAGFIGCALSQRLASSFTRWVVVDSLHPQVHPERVRPDGLHDSAELVVGDITKSATWDQVLSDIRPDIVIHLAAETGTAQSLDEASRHAEVNVVGTTRMLDGFGRHGIVPERILLSSSRAVYGEGRWISERGDVRYPGQRSHGQLERGAWDFDGLAPLPARAGDNVPHPTSVYGSTKLSQEQILAAWVGARDTALTVLRLQNVFGPGQSLTNSYTGIVSLFSQLARAGQSIPIYEDGEITRDFVFIDDIADAFVAALGTARSVGTTTFDVGSGVPSTINDLARTIARHHGAPAPHITGAFRDGDVRFAACDVAPTVAALDWQPQWNLDAGVVRLQDWIDGQLGTPVEALMAG

>WP_130658431.1 NAD-dependent epimerase/dehydratase family protein [Rhizobium leguminosarum]

MIEAKRSTCIVTGGAGFIGCALSGELVRRFDRVIVIDSMHPQVHTTPERPKALAASAELVVGDVAEPAVWELVLDSALPTCIIHLAAETGTGQSLTQSTRHVRANLMGTSVMLDALSRRDIVPDQFVLTSSRAVYGEGAWRTAEGALVYPGQRDAEQLEAGEWDFPGLSFEEFSCGKTVPTPTNIYAATKLAQEHLLSAWVQAYGSSLDIARLQNVYGPGQALQNSYTGIVCLFARLAQSKERIELYEDGEMLRDFVYIEDVTSALIAAIDAPHEAVRTFDVGSGRRGTISEAAHILARHYDAPEPRVSGAYRQGDVRHAACDISPTLQMLQWAPRWTLEKGLHALCDWMEKA

>PXX39323.1 dTDP-L-rhamnose 4-epimerase [Burkholderia pyrrocinia]

MSERNCLVTGGAGFIGSALSQQLVSRFDKVVAVDCLHPQIHAKQTRPAALHPGVELVVGDVCDATVWDNVLASFRPEVVVHLAAETGTGQSLTEATRHAHTNVVGTTAMLDAFMRHDAKPKRIVLSSSRAVYGEGAWQGNEGGSLFYPGQRSVSQLAAGKWNFDGFPVAMEADRVHPAPVSVYGATKLAQEHVISSWANAVGTEYVILRLQNVFGPGQSLINSYTGIVSLFCQLARKKQSIPLYEDGMVMRDFILIDDIAAALFAASTVTGISGRVFDIGSGVATTLLQLAEKIAALYGAPAPEVVGKYRFGDVRHAFTSAEGAKGLGWAPKHDLDYGLKVLAQWIEGELQHV
